# Population diversity and antibody selective pressure to *Plasmodium falciparum *MSP1 block2 locus in an African malaria-endemic setting

**DOI:** 10.1186/1471-2180-9-219

**Published:** 2009-10-15

**Authors:** Nitchakarn Noranate, Franck Prugnolle, Hélène Jouin, Adama Tall, Laurence Marrama, Cheikh Sokhna, Marie-Thérèse Ekala, Micheline Guillotte, Emmanuel Bischoff, Christiane Bouchier, Jintana Patarapotikul, Jun Ohashi, Jean-François Trape, Christophe Rogier, Odile Mercereau-Puijalon

**Affiliations:** 1 Institut Pasteur, Unité d'Immunologie Moléculaire des Parasites, CNRS URA 2581, 28 rue du Dr ROUX, 75724 Paris Cedex 15, France; 2 Laboratoire Génétique et Evolution des Maladies Infectieuses, UMR 2724 CNRS-IRD-UMI, Institut de Recherche pour le Développement, 911 Av. Agropolis, BP 64501, 34394 Montpellier Cedex 5, France; 3 Institut Pasteur de Dakar, Unité d'Epidémiologie, BP220, Dakar, Sénégal; 4 Laboratoire de Paludologie/Zoologie Médicale, Institut de Recherche pour le Développement, BP 1386, CP 18524, Dakar, Sénégal; 5 Institut Pasteur, Pasteur Génopole-Ile de France, 28 rue du Dr ROUX, 75724 Paris Cedex 15, France; 6 Faculty of Tropical Medicine, Mahidol University, 420/6 Rajvithi Road, Bangkok 10400, Thailand; 7 Department of Human Genetics, University of Tokyo, 7-3-1 Hongo, Bunkyo-ku, Tokyo113-0033, Japan; 8 Institut de Médecine Tropicale du Service de Santé des Armées, Boulevard Charles Livon, BP46, 13998 Marseille Armées, France

## Abstract

**Background:**

Genetic evidence for diversifying selection identified the Merozoite Surface Protein1 block2 (PfMSP1 block2) as a putative target of protective immunity against *Plasmodium falciparum*. The locus displays three family types and one recombinant type, each with multiple allelic forms differing by single nucleotide polymorphism as well as sequence, copy number and arrangement variation of three amino acid repeats. The family-specific antibody responses observed in endemic settings support immune selection operating at the family level. However, the factors contributing to the large intra-family allelic diversity remain unclear. To address this question, population allelic polymorphism and sequence variant-specific antibody responses were studied in a single Senegalese rural community where malaria transmission is intense and perennial.

**Results:**

Family distribution showed no significant temporal fluctuation over the 10 y period surveyed. Sequencing of 358 PCR fragments identified 126 distinct alleles, including numerous novel alleles in each family and multiple novel alleles of recombinant types. The parasite population consisted in a large number of low frequency alleles, alongside one high-frequency and three intermediate frequency alleles. Population diversity tests supported positive selection at the family level, but showed no significant departure from neutrality when considering intra-family allelic sequence diversity and all families combined. Seroprevalence, analysed using biotinylated peptides displaying numerous sequence variants, was moderate and increased with age. Reactivity profiles were individual-specific, mapped to the family-specific flanking regions and to repeat sequences shared by numerous allelic forms within a family type. Seroreactivity to K1-, Mad20- and R033 families correlated with the relative family genotype distribution within the village. Antibody specificity remained unchanged with cumulated exposure to an increasingly large number of alleles.

**Conclusion:**

The *Pfmsp1 *block2 locus presents a very large population sequence diversity. The lack of stable acquisition of novel antibody specificities despite exposure to novel allelic forms is reminiscent of clonal imprinting. The locus appears under antibody-mediated diversifying selection in a variable environment that maintains a balance between the various family types without selecting for sequence variant allelic forms. There is no evidence of positive selection for intra-family sequence diversity, consistent with the observed characteristics of the antibody response.

## Background

Around 40% of the world's population is at risk from malaria. Current widespread parasite drug resistance and insect pesticide resistance call for urgent development of new control tools, including malaria vaccines. Rationale vaccine development is challenged by the complexity of the life cycle and the large number of potential vaccine targets [1,2]. The search for genetic evidence of diversifying selection has been proposed as a strategy to identify major targets of protective immunity [3]. Several antigens under putative immune selection have been uncovered this way [4-7], including the N-terminal polymorphic domain of the merozoite surface protein-1 (MSP1), called MSP1 block2 [3].

MSP1-block2 shows extensive allelic polymorphism, with over 120 variants identified worldwide, grouped into three families or types and one recombinant type [8-21]. In parasite populations from Africa and Southeast Asia, *Pfmsp1 *block2 showed a low inter-population variance, with a very low *F*_ST _value, suggesting strong balancing selection to maintain family types within each population [3]. In agreement with this, *in vitro *inhibition of *P. falciparum *cultures by monoclonal antibodies reacting with MSP1 block2 was family-specific [22]. Studies in humans exposed to malaria showed that antibodies to MSP1 block2 were family-specific (also called type-specific by some authors) [3,23-33]. The same was observed in mice immunised with recombinant proteins derived from reference alleles from each family [27,34]. Importantly, presence of antibodies to recombinant proteins of the K1- and MAD20 types was negatively associated with clinical malaria in prospective studies in Gambian [3,23] and Ghanaian children [24]. In contrast, levels of anti-MSP1 block2 IgG were positively associated with an increased risk of subsequent reinfection and/or a lower ability to control parasitaemia in older individuals in Mali [35]. Thus, the involvement of antibodies to MSP1 block2 in parasite control and protection is still unclear.

The K1 and MAD20 MSP1 block 2 families are characterised by the presence of central three amino acids repeats. The various K1- and MAD20-type block2 alleles differ in the number, sequence and relative arrangement of tripeptide repeats and in point mutation polymorphism of the flanking regions. The non-repetitive RO33 alleles only differ by point mutations [8]. The fourth family type called MR, which has been identified recently, results from recombination between the Mad20 and RO33 families [11,16]. Within each MSP1 block2 family, multiple sequence variants have been described.

Analysis of antibody responses in humans living in endemic areas using up to four full length recombinant proteins per family alongside recombinant sub-domains such as repeats only or flanking regions expressed in *Escherichia coli *[3,23-25,28,30-33,36] showed family-specific responses, with no inter-family cross-reactivity. Antibodies to specific sub-types within each family were observed as well [23,25,28,31], and their prevalence varied with malaria transmission conditions [23,24,28]. Monitoring of the antigenic consequences of sequence variation at the single epitope level was done using arrays of synthetic peptides [15,26,27,29]. Interestingly, this showed that sera from mice immunised with a full length recombinant protein reacted with peptides derived from the immunising allele but not with any of its sequence variants [23,27]. Sequence-dependent specificity of individual epitopes was similarly outlined using monoclonal antibodies [15,22,37]. In African populations exposed to *P. falciparum*, the response to MSP1 block2, assessed using synthetic sequence variants displayed a restricted specificity [15,26,27].

The antibody response to MSP1-block2 correlated with PCR typing of the parasites present at the time of plasma collection in some settings [25], weakly in some others [3,31] and not in others [27,33]. In Senegal, fine specificity of the antibodies to MSP1 block2 did not match with the infecting type and moreover was fixed over time, with no novel antibody specificity acquired upon cumulated exposure to multiple infections [27]. Interpretation of these studies has been limited insofar as molecular sequence data and sequence-specific serological responses were not gathered from the same population/setting [15], or sequence data were generated without exploring the immune response [9-14,16,17] or alternatively, immunological responses were studied without detailed knowledge of the actual sequence polymorphism of the local population [23-28,30,33]. Thus, whether the acquired antibodies to MSP1 block2 select for parasites presenting novel sequence variants and exert a significant diversifying selection at the epitope level remains to be studied.

We set out to address this question and analysed *Pfmsp1 *block2 sequence polymorphism and sequence-specific antibody responses using archived samples collected in Dielmo, a Senegalese rural setting. We have analysed sequence polymorphism of the locus over a 10 year period to gain a view of its overall polymorphism and possible temporal evolution. We have explored the humoral response of the villagers to MSP1 block2 using synthetic peptides displaying numerous sequence variants. Serological studies have included a cross-sectional study to measure point prevalence at the village level before a rainy season, a prospective study to explore the relationship between the presence of antibodies to MSP1 block2 at enrolment and protection from clinical malaria episodes during the following five months of intense transmission, and longitudinal follow up of individuals to study temporal antibody variation. This showed evidence for family-specific responses possibly exerting a balancing selection, but gave no support to the notion of antibody selection for variant sequence alleles.

## Results

### *Pfmsp1 *block2 PCR genotyping: distribution of allelic families

**Figure 1:**
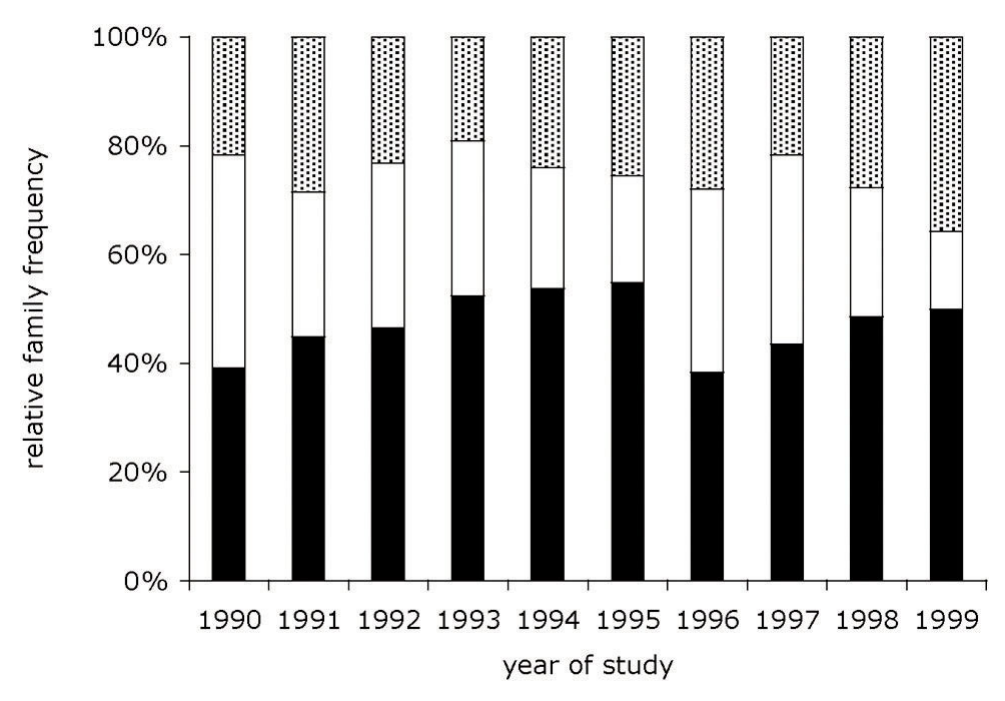
**Temporal distribution of the relative proportion of the three allelic families in Dielmo during 1990-99**. Alleles were assigned to one of three allelic families by nested PCR. Distribution is shown by calendar year. The number of samples typed each year is shown in Table 1. Colour symbols: black: K1-types, white: Mad20-types, grey RO33 types. Note that hybrid alleles were not distinguished from the Mad20-types and are included in the Mad20 group.

A total of 306 samples were successfully genotyped by semi-nested PCR. Overall 524 PCR fragments were generated (Table 1). There were 247, 145 and 132 fragments assigned to the K1, Mad20 and RO33 allelic families, respectively. Based on fragment size polymorphism, 32 and 23 K1-type and Mad20-type alleles could be identified [see Additional file 1]. All RO33 fragments were of the same size. The family frequencies were 47%, 28% and 25% for K1, Mad20 and RO33, respectively. The relative proportion of the three allelic families (Figure 1) did not show significant temporal fluctuations (Pearson test, Chi2 = 14.99; p = 0.663), was not influenced by age (Fisher's exact test, p = 0.813), gender (*idem*, p = 0.45), β-globin type (*idem*, p = 0.678 for AA vs. AS; p = 0.923 AA vs. AS vs. other β-globin variants), ABO blood group (*idem *p = 0.688) or Rhesus blood group (*idem *p = 0. 390).

**Table 1:** Number of isolates studied by calendar year of survey and successfully genotyped for the *Pfmsp1 *block2 locus by nested PCR and gene sequencing

		PCR genotyping			Sequencing
		
year of survey	No samples studied	No samples typed	No alleles detected	Mean No alleles detected/sample	No PCR fragments sequenced
1990	23	23	46	2,00	27
1991	30	29	49	1,69	32
1992	30	29	43	1,48	33
1993	37	36	63	1,75	45
1994	35	34	54	1,59	37
1995	38	33	51	1,55	40
1996	46	38	68	1,79	48
1997	26	25	46	1,84	29
1998	52	44	76	1,73	51
1999	19	15	28	1,87	16

Many samples contained more than one *Pfmsp1 *block2 type. The average multiplicity of infection estimated from the number of fragments detected (estimated moi - see Methods) was 1.73 *Pfmsp1 *block2 fragments/sample. This figure does most probably not reflect the actual number of distinct clones present in the patient, as distinct *Pfmsp1 *block2 alleles yet of similar size are not taken into account and as parasites with identical *Pfmsp1 *block2 alleles may differ in multiple other loci across their genome.

**Figure 2:**
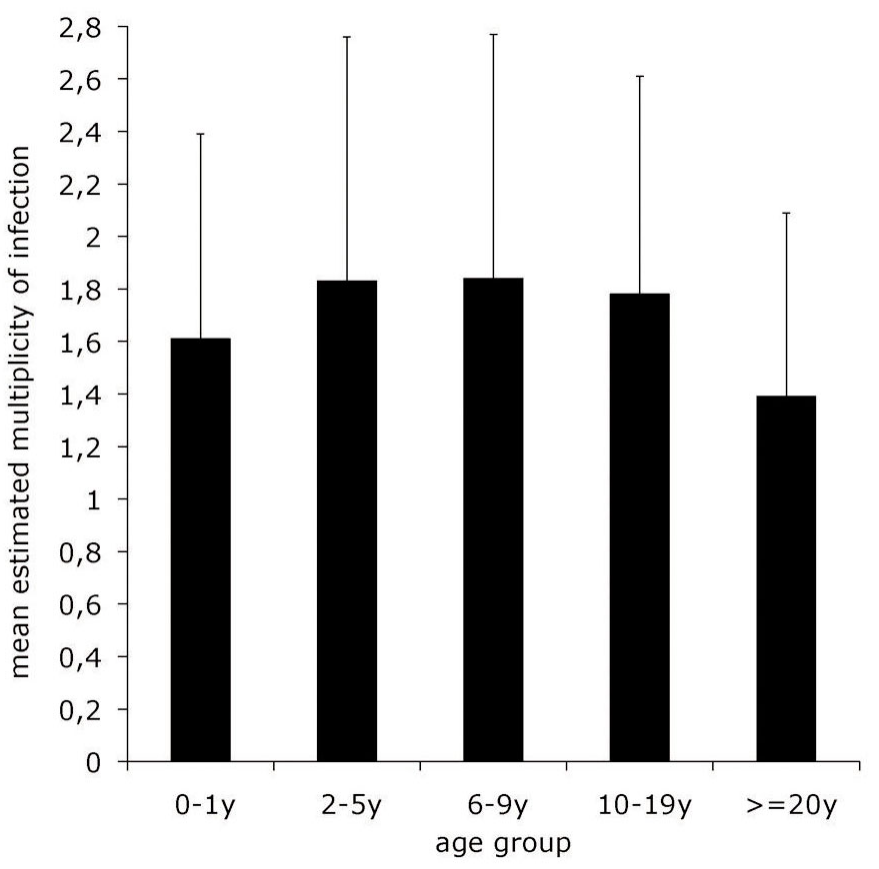
**Estimated multiplicity of infection by age group**. Estimated multiplicity of infection (i.e. the mean number of *Pfmsp1 *block 2-alleles detected per sample) was calculated from PCR fragments generated in the nested PCR reaction. There were 51, 83, 61, 60 and 51 samples in the 0-1 y, 2-5 y, 6-9 y, 10-19 y and ≥20 y age groups, respectively. The figures shown are the mean and SD.

The number of *Pfmsp1 *block2 fragments detected was influenced by age (Kruskal Wallis test, p = 0.0192) (Figure 2); it was highest in the 2-5 y and 6-9 y old children and lowest in the ≥ 20 y old. It was not associated with gender (Kruskal Wallis test, p = 0.670), β-globin type (idem, p = 0.482), ABO or Rhesus blood group (idem, p = 0.234 and p = 0.839, respectively) or with year of study (idem, p = 0.508).

### Analysis of infection rates by individual allelic families

**Figure 3:**
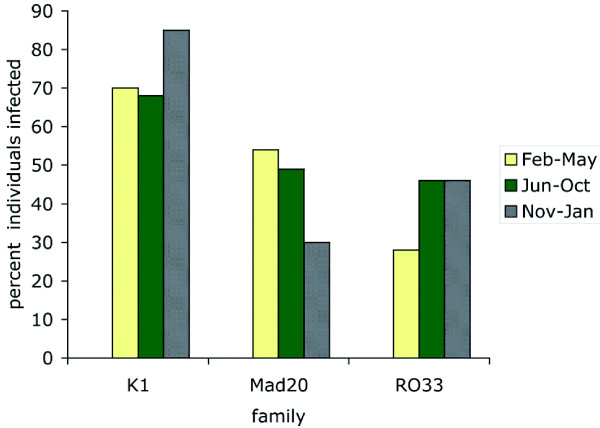
**Influence of seasonality on *Pfmsp1 *block 2 family infection rates**. Data from individual years were pooled. Three seasons were defined as February-May (yellow), June-October (green) and November-January (hatched grey).

One or more K1-type and Mad-type 20 alleles were detected in 73% and 44% of the samples, respectively, while the RO33 family was observed in 43% of the patients. For each of the three families, the infection rate was not associated with gender (Fisher's exact test p = 0.164, 0.260, 0.289 for K1, Mad20 and RO33, respectively), β-globin type (Fisher's exact test p = 0.498, 0.704 and 0.384 for K1, Mad20 and RO33 respectively), ABO blood group (Fisher's exact test p = 0.195, 0.721 and 0.467 for K1, Mad20 and RO33, respectively) and Rhesus blood groups (Fisher's exact test p = 1.000, 0.268 and 0.370 for K1, Mad20 and RO33, respectively). Seasonality did, however, have an influence (Figure 3). The infection rates of K1-types were higher and those of Mad20-types lower in the November-January period (mean no. infected bites/month ± SD = 15.42 ± 10.07) than in February-May (*idem *= 10.78 ± 8.54) or June-October (*idem *= 31.53 ± 18.14) (Fishers' exact test p = 0.011 and p = 0.005, respectively). The RO33-type infection rates tended to be lower in February-May compared to the two other periods (Fishers' exact test, p = 0.061).

### *Pfmsp1 *sequences

Direct sequencing generated high quality sequences on both strands for 358 fragments. The 358 sequences obtained accounted for 58% (144 of 247), 62% (90 of 145), and 94% (124 of 132) of the amplified K1, MAD20 and RO33 fragments, respectively, with a fair temporal distribution of sequenced fragments [see Additional file 2].

There was a large nucleotide sequence diversity, with a total of 126 alleles. In addition to the K1, Mad 20 and RO33 families, the fourth MR hybrid family identified recently [11,16], was observed (Table 2). The six RO33-type alleles (RD0-5) differed only by point mutations. In addition to the previously reported G97D polymorphism [9] [see Additional file 3] four novel positions were dimorphic (Q72E, K90N, G91D and D104N). The K1 family presented the highest diversity, with 77 distinct K1 alleles (DK1-77). Sequencing of 90 fragments assessed by nested PCR to the Mad20 resulted in identifying only 34 distinct Mad20-type alleles (DM1-34) and nine hybrid types (DMR1-8, and DMRK).

**Table 2:** Nucleotide sequence diversity of the 126 *Pfmsp1 *block2 alleles detected in Dielmo, Senegal.

	code	AA repeat	nucleotide sequence
K1 family			
	1	SGT	AGT GGT ACA
	2	SGP	AGT GGT CCA
	3	SAQ	AGT GCT CAA
	4	SGA	AGT GGT GCA
	7	SVT	AGT GTT ACA

MAD20 family			
	5	SGG	TCA GGT GGT
	5	SGG	TCA GGT GGC
	6	SVA	TCA GTT GCT
	7	SVT	TCA GTT ACT
	8	SKG	TCA AAG GGT
	9	SSG	TCA AGT GGT

	Allele	Repeat motifs	24 AA family-specific region

K1 family *Group1*	DK1	3 1 1 1 1 1 1 1 1 1 1 1 1 1 1 2 1	*SPSSRSNTLPRSNTSSGASPPADA*
	DK 2	3 1 1 1 1 1 1 1 1 1 1 1 1 1 2 2 1	*SPSSRSNTLPRSNTSSGASPPADA*
	DK 3	3 1 1 1 1 1 1 1 1 1 1 1 2 2 1	*SPSSRSNTLPRSNTSSGASPPADA*
	DK 4	3 1 1 1 1 1 1 1 1 1 1 1 3 1 3 1 2 2 1	*SPSSRSNTLPRSNTSSGASPPADA*
	DK 5	3 1 1 1 1 1 1 1 1 1 1 2 2 1	*SPSSRSNTLPRSNTSSGASPPADA*
	DK 6	3 1 1 1 1 1 1 1 1 1 1 3 1 3 1 3 1 1 1 1 2 1	*SPSSRSNTLPRSNTSSGASPPADA*
	DK 7	3 1 1 1 1 1 1 1 1 1 2 2 1	*SPSSRSNTLPRSNTSSGASPPADA*
	DK 8	3 1 1 1 1 1 1 1 2 2 2 2 1	*SPSSRSNTLPRSNTSSGASPPADA*
	DK 9	3 1 1 1 1 1 1 2 2 1	*SPSSRSNTLPRSNTSSGASPPADA*
	DK 10	3 1 1 1 1 1 1 2 2 2 2 2 2 2 2 2 1	*SPSSRSNTLPRSNTSSGASPPADA*
	DK 11	3 1 1 1 1 3 1 3 1 3 1 1 1 2 1	*SPSSRSNTLPRSNTSSGASPPADA*
	DK 12	3 1 1 1 1 3 1 3 1 3 1 1 2 2 1	*SPSSRSNTLPRSNTSSGASPPADA*
	DK 13	3 1 1 1 1 3 1 3 1 3 1 2 1	*SPSSRSNTLPRSNTSSGASPPADA*
	DK 14	3 1 1 1 2 1 2 1 2 1 1 2 2 1	*SPSSRSNTLPRSNTSSGASPPADA*
	DK 15	3 1 1 1 2 1 2 1 2 1 2 1 2 2 1	*SPSSRSNTLPRSNTSSGASPPADA*
	DK 16	3 1 1 1 3 1 1 1 3 1 1 1 3 1 2 2 1	*SPSSRSNTLPRSNTSSGASPPADA*
	DK 17	3 1 1 1 3 1 1 3 1 1 3 1 1 2 2 1	*SPSSRSNTLPRSNTSSGASPPADA*
	DK 18	3 1 1 2 1 2 1 2 1 2 1 2 1	*SPSSRSNTLPRSNTSSGASPPADA*
	DK 19	3 1 1 2 1 2 1 2 1 2 2 1	*SPSSRSNTLPRSNTSSGASPPADA*
	DK 20	3 1 1 2 1 2 1 2 2 1	*SPSSRSNTLPRSNTSSGASPPADA*
	DK 21	3 1 2 1 2 1 2 1 1 1 1 2 2 2 2 1	*SPSSRSNTLPRSNTSSGASPPADA*
	DK 22	3 1 2 1 2 1 2 1 1 1 2 2 1	*SPSSRSNTLPRSNTSSGASPPADA*
	DK 23	3 1 2 1 2 1 2 1 2 2 1 2 2 1	*SPSSRSNTLPRSNTSSGASPPADA*
	DK 24	3 1 2 1 2 1 2 1 2 2 2 2 1	*SPSSRSNTLPRSNTSSGASPPADA*
	DK 25	3 1 2 1 2 1 2 2 1	*SPSSRSNTLPRSNTSSGASPPADA*
	DK 26	3 1 2 1 2 2 2 2 2 2 2 2 2 2 2 2 2 2 1	*SPSSRSNTLPRSNTSSGASPPADA*
	DK 27	3 1 3 1 3 1 1 1 1 1 1 1 1 1 1 1 1 1 1	*SPSSRSNTLPRSNTSSGASPPADA*
	DK 28	3 3 1 1 1 1 1 1 1 1 1 1 1 2 2 1	*SPSSRSNTLPRSNTSSGASPPADA*

K1 family *Group2*	DK 29	3 4 3 1 1 1 1 1 1	*SPSSRSNTLPRSNTSSGASPPADA*
	DK 30	3 4 3 1 1 1 1 1 1 1	*SPSSRSNTLPRSNTSSGASPPADA*
	DK 31	3 4 3 1 1 1 1 1 1 1 1 1 2 2 1	*SPSSRSNTLPRSNTSSGASPPADA*
	DK 32	3 4 3 1 1 1 1 1 1 1 1 2 2 1	*SPSSRSNTLPRSNTSSGASPPADA*
	DK 33	3 4 3 1 1 1 1 1 1 2 2 1	*SPSSRSNTLPRSNTSSGASPPADA*
	DK 34	3 4 3 1 1 1 1 1 2 2 1	*SPSSRSNTLPRSNTSSGASPPADA*
	DK 35	3 4 3 1 1 1 1 2 2 1	*SPSSRSNTLPRSNTSSGASPPADA*
	DK 36	3 4 3 1 1 1 2 1	*SPSSRSNTLPRSNTSSGASPPADA*
	DK 37	3 4 3 1 1 1 2 2 2 1	*SPSSRSNTLPRSNTSSGASPPADA*
	DK 38	3 4 3 1 2 1 2 1 2 1 2 1 2 2 1	*SPSSRSNTLPRSNTSSGASPPADA*
	DK 39	3 4 3 1 2 2 1	*SPSSRSNTLPRSNTSSGASPPADA*
	DK 40	3 4 3 1 2 2 2 1	*SPSSRSNTLPRSNTSSGASPPADA*
	DK 41	3 4 3 1 3 1 2 2 1 2 2 1	*SPSSRSNTLPRSNTSSGASPPADA*
	DK 42	3 4 3 1 3 1 3 1 1 1	*SPSSRSNTLPRSNTSSGASPPADA*
	DK 43	3 4 3 1 3 1 3 1 1 1 1 1 1 1 1 1 2 1	*SPSSRSNTLPRSNTSSGASPPADA*
	DK 44	3 4 3 1 3 1 3 1 1 1 1 1 1 2 1 1 2 1	*SPSSRSNTLPRSNT LSGASPPADA*
	DK 45	3 4 3 1 3 1 3 1 1 1 1 1 2 2 1	*SPSSRSNTLPRSNTSSGASPPADA*
	DK 46	3 4 3 1 3 1 3 1 1 1 1 2 2 1	*SPSSRSNTLPRSNTSSGASPPADA*
	DK 47	3 4 3 1 3 1 3 1 1 1 2 2 1	*SPSSRSNTLPRSNTSSGASPPADA*
	DK 48	3 4 3 1 3 1 3 1 1 2 1 2 1	*SPSSRSNTLPRSNTSSGASPPADA*
	DK 49	3 4 3 1 3 4 3 4 3 4 3 1 2 2 1	*SPSSRSNTLPRSNTSSGASPPADA*
	DK 50	3 4 3 1 7 2 2 1	*SPSSRSNTLPRSNTSSGASPPADA*
	DK 51	3 4 3 4 3 1 1 1 1	*SPSSRSNTLPRSNTSSGASPPADA*
	DK 52	3 4 3 4 3 1 1 1 1 1	*SPSSRSNTLPRSNTSSGASPPADA*
	DK 53	3 4 3 4 3 1 1 1 1 1 1 1 1 1 1 1	*SPSSRSNTLPRSNTSSGASPPADA*
	DK 54	3 4 3 4 3 1 1 1 2 2 2 1	*SPSSRSNTLPRSNTSSGASPPADA*
	DK 55	3 4 3 4 3 1 1 2 2 1	*SPSSRSNTLPRSNTSSGASPPADA*
	DK 56	3 4 3 4 3 1 1 3 4 3 1 2 2 1	*SPSSRSNTLPRSNTSSGASPPADA*
	DK 57	3 4 3 4 3 1 2 1	*SPSSRSNTLPRSNT LSGASPPADA*
	DK 58	3 4 3 4 3 1 2 1 2 1 2 2 2 1 2 2 2 1 2 2 1	*SPSSRSNTLPRSNTSSGASPPADA*
	DK 59	3 4 3 4 3 1 2 2 1	*SPSSRSNTLPRSNTSSGASPPADA*
	DK 60	3 4 3 4 3 1 2 2 2 1	*SPSSRSNTLPRSNTSSGASPPADA*
	DK 61	3 4 3 4 3 1 3 1 3 1 3 1 3 1	*SPSSRSNTLPRSNTSSGASPPADA*
	DK 62	3 4 3 4 3 4 3 1 1 1 1 1 1	*SPSSRSNTLPRSNTSSGASPPADA*
	DK 63	3 4 3 4 3 4 3 1 1 1 1 1 1 1 1	*SPSSRSNTLPRSNTSSGASPPADA*
	DK 64	3 4 3 4 3 4 3 1 2 1	*SPSSRSNTLPRSNTSSGASPPADA*
	DK 65	3 4 3 4 3 4 3 1 2 2 1	*SPSSRSNTLPRSNTSSGASPPADA*
	DK 66	3 4 3 4 3 4 3 1 2 2 1 1	*SPSSRSNTLPRSNTSSGASPPADA*
	DK 67	3 4 3 4 3 4 3 1 3 1 1 1 1 1 2 1	*SPSSRSNTLPRSNT LSGASPPADASPPADA*
	DK 68	3 4 3 4 3 4 3 4 3 1 1	*SPSSRSNTLPRSNTSSGASPPADA*
	DK 69	3 4 3 4 3 4 3 4 3 1 1 1 1	*SPSSRSNTLPRSNTSSGASPPADA*
	DK 70	3 4 3 4 3 4 3 4 3 1 2 2 1	*SPSSRSNTLPRSNTSSGASPPADA*
	DK 71	3 4 3 4 3 4 3 4 3 1 2 2 2 1	*SPSSRSNTLPRSNTSSGASPPADA*
	DK 72	3 4 3 4 3 4 3 4 3 1 3 1 1 1 1 1	*SPSSRSNTLPRSNTSSGASPPADA*
	DK 73	3 4 3 4 3 4 3 4 3 1 3 1 1 1 2 2 1	*SPSSRSNTLPRSNTSSGASPPADA*
	DK 74	3 4 3 4 3 4 3 4 3 4 3 1 2 2 1	*SPSSRSNTLPRSNTSSGASPPADA*
	DK 75	3 4 3 4 3 4 3 4 3 4 3 1 7 2 2 1	*SPSSRSNTLPRSNTSSGASPPADA*
	DK 76	3 4 3 4 3 4 3 4 3 4 3 4 3 1 2 2 1	*SPSSRSNTLPRSNTSSGASPPADA*
	DK 77	3 4 3 4 3 4 3 4 3 4 3 4 3 4 3 4 3 1 2 2 1	*SPSSRSNTLPRSNTSSGASPPADA*

	Allele	repeat motifs	13 AA family-specific region

MAD20 family *Group1*	DM1	5 6 5 5 6 5 5 6 5 5 6 5 5 6 5 5 6 5	*SG -----------------NS*
	DM 2	5 6 5 5 6 5 5 6 5 5 6 5 5 6 5	*SG -----------------NS*
	DM 3	5 6 5 5 6 5 5 6 5	*SG -----------------NS*
	DM 4	5 6 5 6 5 6 5 6 5	*SGNSRRTNPSDNS*
	DM 5	5 6 5 6 5 6 5	*SGNSRRTNPSDNS*
	DM 6	5 7 5 5 5 6 5 6 5 5 6 5 6 5	*SGNSRRTNPSDNS*
	DM 7	5 7 5 5 6 5 6 5 5 6 5 6 5	*SGNSRRTNPSDNS*
	DM 8	5 7 5 5 5 6 5 6 5 5 6 5	*SGNSRRTNPSDNS*
			
MAD20 family *Group2*	DM 9	8 5	*SGNSRRTNPSDNS*
	DM 10	8 5 6 5 6 5 6 5 6 5 6 5 6 5 6 5 5 6 5	*SGNSRRTNPSDNS*
	DM 11	8 5 6 5 5 6 5 5 6 5 5 6 5 6 5 6 5	*SGNSRRTNPSDNS*
	DM 12	8 5 6 5 5 5 6 5 6 5 5 6 5 6 5	*SGNSRRTNPSDNS*
	DM 13	8 5 6 5 5 6 5 5 6 5 5 6 5 6 5	*SGNSRRTNPSDNS*
	DM 14	8 5 6 6 5 6 5 5 6 5 5 6 5 6 5	*SGNSRRTNPSDNS*
	DM 15	8 5 6 5 5 6 6 5 6 6 5 6 5	*SGNSRRTNPSDNS*
	DM 16	8 6 5 5 6 5 6 5 5 6 5 5 6 5 5 6 5 6 5	*SGNSRRTNPSDNS*
	DM 17	8 6 5 6 5 6 5 5 5 6 5 6 5 6 5 6 5	*SGNSRRTNPSDNS*
	DM 18	8 6 5 6 5 6 5 6 5 5 6 5 5 6 5 6 5	*SGNSRRTNPSDNS*
	DM 19	8 6 5 6 5 5 5 6 5 6 5 6 5 6 5	*SGNSRRTNPSDNS*
	DM 20	8 6 5 6 5 6 5 5 5 6 5 6 5 6 5	*SGNSRRTNPSDNS*
	DM 21	8 6 5 6 5 6 5 5 6 5 5 6 5 6 5	*SGNSRRTNPSDNS*
	DM 22	8 6 5 6 5 5 5 6 5 6 5 6 5	*SGNSRRTNPSDNS*
	DM 23	8 6 5 6 5 5 6 5 5 6 5 5 6 5	*SGNSRRTNPSDNS*
	DM 24	8 6 5 5 6 5 5 6 5 6 5 6 5	*SGNSRRTNPSDNS*
	DM 25	8 6 5 6 5 6 5 5 5 6 5 6 5	*SGNSRRTNPSDNS*
	DM 26	8 6 5 5 5 6 5 6 5 6 5	*SGNSRRTNPSDNS*
	DM 27	8 6 5 5 6 5 5 6 5 6 5	*SGNSRRTNPSDNS*
	DM 28	8 6 5 6 5 5 5 6 5 6 5	*SGNSRRTNPSDNS*
	DM 29	8 6 9 6 5	*SGNSRRTNPSDNS*
	DM 30	8 7 5 5 5 6 5 5 6 5 5 5 6 5 6 5 6 5	*SGNSRRTNPSDNS*
	DM 31	8 7 5 5 5 5 6 5 5 6 5 5 6 5 6 5	*SGNSRRTNPSDNS*
	DM 32	8 7 5 5 5 6 5 5 6 5 5 6 5 6 5	*SGNSRRTNPSDNS*
	DM 33	8 7 5 5 5 6 5 6 5 6 5 6 5 6 5	*SGNSRRTNPSDNS*
	DM 34	8 7 5 5 6 5 5 6 5 5 6 5 6 5	*SGNSRRTNPSDNS*

RO33-family		mutation	sequence

	RD0		KPADAVSTQSAKNPPGATVPSGTASTKGAIRSPGAANPSDDS
	RD1	Q72E	KPADAVSTESAKNPPGATVPSGTASTKGAIRSPGAANPSDDS
	RD2	K90N	KPADAVSTQSAKNPPGATVPSGTASTNGAIRSPGAANPSDDS
	RD3	G91D	KPADAVSTQSAKNPPGATVPSGTASTKDAIRSPGAANPSDDS
	RD4	G97D	KPADAVSTQSAKNPPGATVPSGTASTKGAIRSPDAANPSDDS
	RD5	G97D/D104N	KPADAVSTQSAKNPPGATVPSGTASTKGAIRSPDAANPSDNS

Hybrids	Allele	MAD20 repeat motif	C-terminal specific region

	DMR1	5 7 5 5 4	TVPSGTASTKGAIRSPDAANPSDNS
	DMR 2	8 7 5 4	TVPSGTASTKGAIRSPDAANPSDNS
	DMR 3	8 7 5 5 4	TVPSGTASTKGAIRSPDAANPSDNS
	DMR 4	8 7 5 5 5 4	TVPSGTASTKGAIRSPDAANPSDNS
	DMR 5	8 7 5 5 5 6 4	TVPSGTASTKGAIRSPDAANPSDNS
	DMR 6	8 7 5 5 5 5 4	TVPSGTASTKGAIRSPDAANPSDNS
	DMR 7	8 7 5 5 5 5 5 4	TVPSGTASTKGAIRSPDAANPSDNS
	DMR 8	8 7 5 5 5 5 5 5 4	TVPSGTASTKGAIRSPDAANPSDNS
	DMRK	8 7 5 5 5 5 4	TVPSGTASTKGAIRSPDAASPPADA

Codes for K1- and Mad20-types tripeptide repeats together with the nucleotide sequence are as proposed in [9,12]. Sequence of the K1-type alleles are grouped as mentioned in the text based on the 5' di-motif and the presence of motif 4 and 7. Sequence of the Mad20-type alleles are grouped as mentioned in the text based on the 5' motifs.

**Figure 4:**
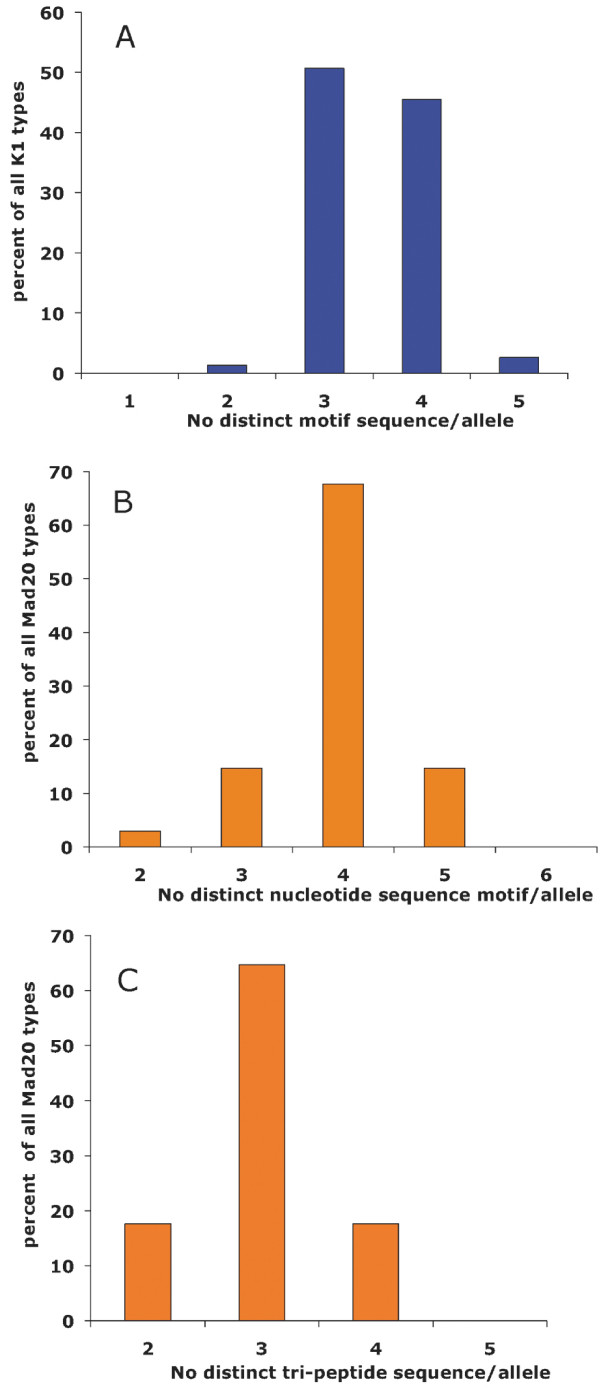
**Frequency distribution of the number of tri-peptide motif usage in the DK and DM alleles**. A: Frequency distribution of K1-type alleles (DK alleles) by number of distinct tripeptides present. B. Frequency distribution of Mad20-types (DM alleles) by number of distinct tripeptide nucleotide sequences present (DMR, DMRK and MK hybrids excluded). C. Frequency distribution of Mad20-types DM alleles (by number of distinct tripeptide protein sequences present (DMR, DMRK and MK hybrids excluded).

The K1- Mad20- and hybrid-types mainly differed in copy number and arrangement of sequence motifs called in the literature "tripeptide motifs", while presenting no or minimal polymorphism of the flanking family-specific sequence. Five distinct tripeptide motifs were observed in the K1 types. In addition to the known SGT, SGP, SAQ and SGA motifs (coded 1-4, respectively according to [9,12]), a novel motif was observed (motif 7 in Table 2), which encodes the SVT tripeptide sequence displayed so far only by Mad20 types. Motif 7 presented a typical K1-type signature, with AGT coding for Ser, as opposed to a TCA/G codon in the Mad20 types. All K1-type alleles contained more than one motif sequence, resulting in eleven di-motif combinations (hexapeptides). Most alleles had three or four different motifs (Figure 4A). Some di-motifs were very frequent and motif 3 1 was present in all alleles [see Additional file 4]. A clear dichotomy could be delineated based on the first 5' di-motif being either 3 1 (group 1, 28 alleles) or 3 4 (group 2, 49 alleles) (with the exception of allele 28 which displayed a 3 3 motif). Limited polymorphism was observed in the 3' family-specific region, with a non-synonymous S to L (tca>tta) mutation, observed in three alleles, and a six amino acid insertion, *SPPADA*, observed in a single allele (Table 2).

Similar findings were observed for the Mad20 types alleles, which differed mainly in the number, arrangement and coding sequence of six tripeptide motifs (coded 5-9). There were two synonymous sequences coding for SGG (5 and 5) such that all Mad20-type alleles contained an SGG-encoding motif [see Additional file 4]. In this family too, all alleles contained more than one motif sequence. The majority had four distinct nucleotide sequence motifs (Figure 4B), encoding three different tripeptide sequences (Figure 4C). Some di-motifs were highly represented, with the SVA SGG motif (6 5 or 6 5) being present in virtually all alleles. There was a dichotomy within the family based on the first 5' motif, being either 5/5 (group 1, 8 alleles) or 8 (group 2, 26 alleles) (Table 2). This group-specific 5' end was followed by a variable copy number and arrangement of six di-motif sequences, which at the protein level translated into variable combinations of the SGG and SVA tripeptides. All Mad20-type block2 repeats except two (DM9 and DM29) terminated with the (5 6 5) sequence. The flanking non repeated region upstream from the tripeptide motifs was identical in all alleles. Downstream from the repeats, a 9 amino acid deletion (*NSRRTNPSD*) was observed in three alleles, but otherwise the family-specific region was monomorphic.

Sequencing showed that 22 fragments assigned to the Mad20 family by semi-nested PCR were indeed Mad20/RO33 (MR) hybrids. We are confident that these alleles are *bona fide *hybrids and not artifactual PCR products, as they have been observed in 14 of 22 samples where a RO33 allele could not be detected using multiple family-specific nested PCR reactions. Moreover, 7 of 22 samples where the MR allele was detected by sequencing were monoinfections (i.e. there were no two partners for template switching). This MR hybrid family was quite diverse as eight alleles were observed. Allele DMR1 had a group1 type Mad20 while alleles DMR 2-8 derived from Mad20 group 2. All DMR alleles carried the same 25-residue long, RO33-type downstream region, which interestingly was a RD5 allelic type with a G97D D104N double mutation (Table 2). A novel hybrid, DMRK, displayed a RO33-K1 hybrid sequence in the family-specific 3' region (the K1 sequence located in 3' is underlined in Table 2) [for further analysis see Additional file 4].

**Figure 5:**
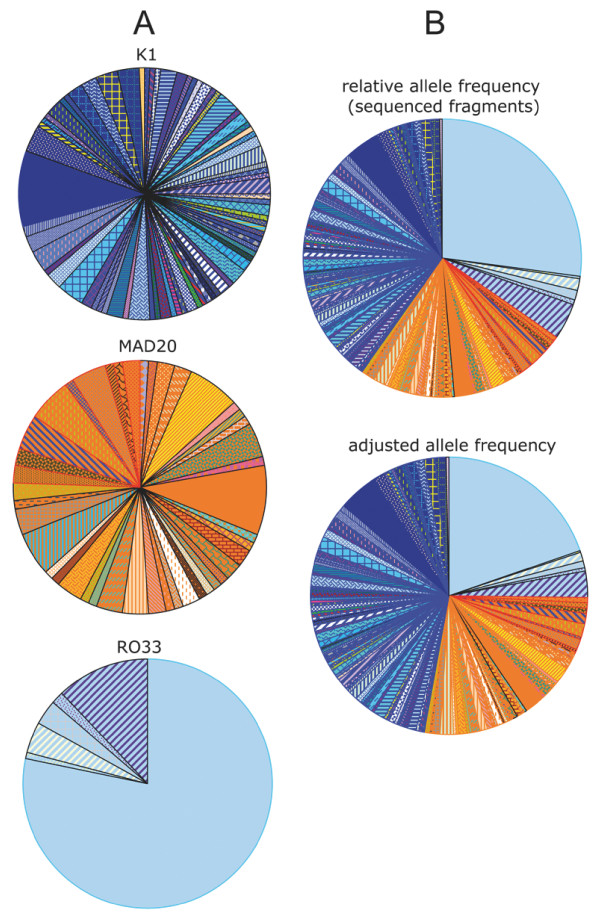
**Distribution of *Pfmsp1 *block2 allele frequency in Dielmo**. A. Distribution by family based on sequenced alleles: K1-types (N sequenced = 144), Mad20-types grouped together with hybrid types (N sequenced = 90) and RO33-types (N sequenced = 124). Each family is depicted separately, with alleles ranked clockwise by allele number coded as shown in Table 2. B. Relative individual allele frequency in the 358 sequenced fragments (top) and adjusted to the overall population based on relative family distribution established by nested PCR on 524 PCR fragments (bottom). Identical colour codes used for A and B, ordered clockwise as follows: RD types (light blue colours), Hybrids (green and orange), DM (orange-yellow) and DK alleles (indigo-dark blue colours), with alleles ranked clockwise by allele number coded as shown in Table 2.

The large local diversity was associated with a large number of low frequency alleles in the K1 and Mad20/MR family types, contrasting with the RO33 family where a dominant RD0 allele was observed in 78% (97 of 124) of the sequenced RO33-types alleles (Figure 5A). At the population level (Figure 5B), RD0 was by far the most frequent allele, accounting for 27% of the sequenced samples (top pie chart) and 19.7% of all alleles within the village when adjusted for relative family frequency estimated by nested PCR genotyping (bottom pie chart). The second most frequent allele after adjusting for family frequency was DK65 (adjusted frequency: 4.6%). Most alleles (107 of 126) presented a less than 1% frequency in the population sample studied here. In terms of frequency, the largest contribution among the top 19 alleles came from the RO33 family.

Sequence comparison of Dielmo alleles with the RO33-, K1-, Mad20-, and MR-types alleles deposited in the database [see Additional file 3], [see Additional file 5], [see Additional file 6], [see Additional file 7], respectively, showed that only 13 of the 126 alleles from Dielmo were identical to reported sequences from other endemic settings, namely K1-types (DK5, -7, -9, -18, -59, -65 -70 and -74), two Mad20-types (DM29 and DM34), two RO-33 types (RD0 corresponding to RO33-Ghana and RD4) and one MR allele (DMR4) [see Additional file 8]. Interestingly, only four of the top 19 alleles have been reported elsewhere in the world, (DK59, DK65, DMR4 and RD0), suggesting local diversification of the parasite population.

### Tests for neutrality

To gain some insight into a possible positive selection on this locus regarding the level (family and/or intra-family) and the type of selection operating, Ewens-Watterson-Slatkin tests for neutrality [38,39] were conducted. At the family level, i.e. grouping alleles by family type considering three families irrespective of size or sequence polymorphism, this showed a significant departure from neutrality on a yearly basis and when grouping all years together, i.e. for a 10 year period (Table 3). Thus, there was evidence for balancing selection at the family level in this setting, the observed homozygosity being lower than expected (Table 3).

**Table 3:** Ewens-Watterson (EW) tests for neutrality for *Pfmsp*1 block2 alleles from Dielmo, Senegal.

Year	Sample size	Observed F	Expected F	p-values				
1990	46	0.3535	0.626	0.0201				
1991	49	0.3536	0.6302	0.021				
1992	43	0.3618	0.6213	0.0317				
1993	63	0.3923	0.6463	0.0584				
1994	54	0.3957	0.6366	0.0662				
1995	51	0.4048	0.633	0.08				
1996	68	0.3387	0.651	0.0051				
1997	46	0.3573	0.626	0.0253				
1998	76	0.3695	0.6575	0.0303				
1999	28	0.398	0.5894	0.099				
All	524	0.3622	0.7429	0.0108				

	Size polymorphism				Size and sequence polymorphism			
Year	N	Observed F	Expected F	p-value	N	Observed F	Expected F	p-value
	
	K1 family							
	
1990	18	0.1728	0.17	0.6612	8	0.1562	0.1562	1
1991	22	0.095	0.099	0.577	11	0.157	0.1542	0.8934
1992	20	0.195	0.1789	0.7793	14	0.0816	0.0816	1
1993	33	0.0964	0.1379	0.0186	20	0.065	0.0607	1
1994	29	0.1249	0.1294	0.551	15	0.0756	0.0756	1
1995	28	0.148	0.1422	0.6971	18	0.1111	0.1113	0.6803
1996	26	0.1775	0.1757	0.6323	18	0.0988	0.1113	0.267
1997	20	0.245	0.1551	0.9901	11	0.0909	0.0909	1
1998	37	0.122	0.1316	0.4808	21	0.1111	0.0962	0.936
1999	14	0.1939	0.2125	0.417	8	0.125	0.125	1
All	247	0.1044	0.0957	0.7197	144	0.0245	0.0214	0.9088

	MAD20 family + Hybrid alleles							
	
1990	18	0.1358	0.1273	0.8024	9	0.1605	0.1683	0.6858
1991	13	0.2071	0.2505	0.2629	8	0.1562	0.1562	1
1992	13	0.1834	0.1698	0.8471	9	0.1111	0.1111	1
1993	18	0.1728	0.1995	0.3267	13	0.1243	0.1208	0.9238
1994	12	0.1667	0.1973	0.2356	9	0.1358	0.1358	1
1995	10	0.32	0.2831	0.9022	9	0.1605	0.1683	0.6858
1996	23	0.2098	0.1906	0.7541	12	0.0972	0.0972	1
1997	16	0.1797	0.1886	0.5808	11	0.1736	0.1885	0.5419
1998	18	0.2037	0.2369	0.3518	10	0.14	0.1455	0.7227
1999	4	0.25	0.25	1	NA	NA	NA	NA
All	145	0.1177	0.1201	0.5816	90	0.0365	0.0407	0.2691

	RO33 family							
	
1990	NA	NA	NA	NA	10	0.66	0.4919	1
1991	NA	NA	NA	NA	13	0.7396	0.7035	0.6469
1992	NA	NA	NA	NA	10	0.68	0.6826	0.6047
1993	NA	NA	NA	NA	12	0.8472	0.6975	1
1994	NA	NA	NA	NA	13	0.3609	0.3976	0.3849
1995	NA	NA	NA	NA	12	0.7222	0.6975	0.6347
1996	NA	NA	NA	NA	18	NA	NA	NA
1997	NA	NA	NA	NA	9	0.358	0.4797	0.1481
1998	NA	NA	NA	NA	20	0.745	0.7331	0.5446
1999	NA	NA	NA	NA	7	0.5102	0.6509	0.2358
All	NA	NA	NA	NA	124	0.6403	0.4419	0.8793

Ewens-Watterson tests by individual year during the 1990-1999 decade and for all years of the decade grouped together (*All*). In the top Table, tests were based on family frequency distribution. Three families were considered: K1, MAD20/Hybrids and RO33. The second part shows the results of the Ewens-Watterson tests within each family with alleles identified by size polymorphism only or both size and sequence polymorphism. For the RO33 family no size polymorphism was observed.

*F*: Homozygosity. *N*: sample size. NA: Not Available.

We then considered the within family diversity of the K1, Mad20/Hybrids (DMR and DMRK) and RO33 alleles separately to look for evidence of selection within each family (Table 3 lower panels). Tests were performed for each year separately or for the 10 year period. Alleles were differentiated by either size polymorphism or both size and sequence polymorphism. Overall, the null hypothesis was not rejected, implying that there was no evidence for significant within-family balancing selection on the *Pfmsp1 *block2 locus. The results of these Ewens-Watterson-Slatkin tests need to be interpreted with caution though. These tests are based on the assumption that no recurrent mutation has occurred at the locus studied. Since the mutation rate is known to be high in minisatellite/repetitive sequences, this assumption may be violated. In other words, one cannot exclude that recurrent mutations may have occurred and in turn have artificially reduced our power to detect balancing selection acting at the intra-family level.

Within the 124 RO33 PCR fragments sampled there was no size polymorphism and six different allele sequences were identified. An alignment of 126 nucleotides for all 124 alleles contained five polymorphic sites, all of which were non-synonymous single nucleotide polymorphisms. This indicates that dN/dS is infinite. Nucleotide diversity (π = average number of differences between any two sequences) was 4.84 × 10^-3^. To examine the possibility of natural selection acting on the RO33 family, Tajima's D and Fu and Li's D* and F* were calculated [40,41]. In view of the high number of segregating sites (N = 5), these tests are expected to show high statistical power for natural selection. No evidence for departure from neutrality was obtained, with non significant Tajima's *D *value, Fu and Li's D* and F* values (Table 4), thus confirming results obtained using the Ewens-Watterson test.

**Table 4:** Neutrality tests for the RO33 family in Dielmo, Senegal

						nucleotide position								amino acid position						
		
allele	mutation				N	197	199	200	214	270	272	290	310	66	67	72	90	91	97	104
RD0	R033				97	C	G	A	C	A	G	G	G	A	D	Q	K	G	G	D
RD1	Q72E				1	.	.	.	G	.	.	.	.	.	.	E	.	.	.	.
RD2	K90N				5	.	.	.	.	T	.	.	.	.	.	.	N	.	.	.
RD3	Q91D				4	.	.	.	.	.	A	.	.	.	.	.	.	D	.	.
RD4	G97D				2	.	.	.	.	.	.	A	.	.	.	.	.	.	D	.
RD5	G97D D104N				15	.	.	.	.	.	.	A	A	.	.	.	.	.	D	N
Year	n	h	S	Ss	(π × 10^-3^)	Tajima's D (P-value)				Fu and Li's D* (P-value)				Fu and Li's F* (P-value)						
		
1990	10	3	2	2	3.17	-1.4009 (>0.1)				-1.5866 (>0.1)				-1.7190 (>0.1)						
1991	13	2	1	0	2.24	- 0.27429 (>0.1)				0.73235 (>0.1)				0.54307 (>0.1)						
1992	10	2	2	0	7.41	1.03299 (>0.1)				1.02623 (>0.1)				1.14601 (>0.1)						
1993	12	2	2	2	2.65	-1.45138 (>0.1)				-1.72038 (>0.1)				1.86451 (>0.1)						
1994	13	4	4	0	8.95	-0.42367 (>0.1)				1.17832 (>0.1)				0.86962 (>0.1)						
1995	12	2	1	0	2.41	-0.19492 (>0.1)				0.75202 (>0.1)				0.58317 (>0.1)						
1996	18	1	0	0	0	-				-				-						
1997	9	3	2	0	8.38	1.49448 (>0.1)				1.06300 (>0.1)				1.28730 (>0.1)						
1998	20	2	2	0	4.26	-0.11187 (>0.1)				0.86615 (>0.1)				0.69109 (>0.1)						
1999	7	2	2	0	9.07	1.64955 (>0.1)				1.17810 (>0.1)				1.37408 (>0.1)						

All	124	6	5	1	4.84	-07033 (>0.1)				-0.0713 (>0.1)				-0.3316 (>0.1)						

Sequence diversity is shown in the upper half of the Table with the nucleotide sequence on the left and the amino acid sequence in single letter code on the right. N: number of isolates.

The lower half of the Table shows the sequence diversity tests by year and all years combined (All)

n: number of samples; h: number of haplotypes; S: number of segregating sites; Ss: number of singleton sites; π: average nucleotide diversity. Tajima's and Fu and Li's tests were implemented by the DnaSP version 4 software, and validated by Fisher's exact tests.

### Anti-MSP1 block2 antibody prevalence and specificity

**Figure 6:**
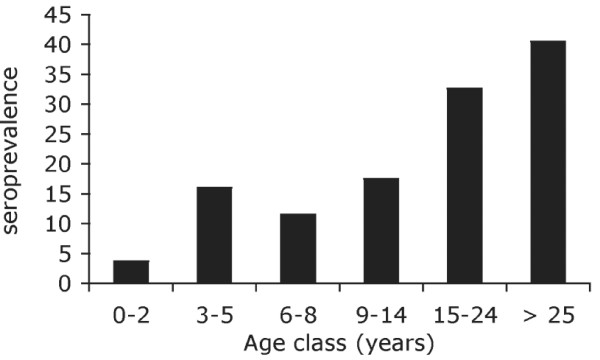
**Prevalence of anti-MSP1-block 2 IgG by age group**. Seroprevalence was determined using sera collected during a cross-sectional survey conducted before the 1998 rainy season (on 2-3 August 1998) when 243 villagers (i.e. 95% of the village population) donated a fingerprick blood sample. The presence of anti-MSP1 block2 specific IgG was assessed by ELISA on 16 pools of biotinylated peptides (sequence and composition of the pools described in Table 5). Plasma reacting with one or more pool was considered seropositive. Number of individuals by age group: 27, 25, 26, 40, 46 and 79 in the 0-2, 3-5, 6-8, 9-14, 15-24, ≥ 25 year group, respectively.

The sequence-specific antibody response was studied by ELISA using biotinylated MSP1 block2-derived peptides bound to streptavidin-coated plates that overall represented a fair coverage of the sequence diversity observed in the village [see Additional file 9]. We recorded as seropositive any individual reacting with one or more peptide. Seroprevalence was analysed at the village level using an archived cross-sectional study conducted at the beginning of the 1998 rainy season, to which 85% of the villagers had contributed. We recorded as seropositive any individual reacting with one or more peptide. Overall, seroprevalence was 25% (62 of 243 sera analysed). Seroprevalence increased with age and reached 40.5% in adults (Figure 6). Confirming previous observations in this setting [26,27], all anti-block2 IgGs were exclusively IgG3 [see Additional file 10]. No anti-block2 IgM was detected.

**Table 5:** Composition of the 16 pools of MSP-1 derived peptides.

**Pool**	**Localisation**	**Peptide sequence**					
P1	Block 1 Mad20	01 TGYSLFQKEKMVLNE	60 TGYGLFQKEKMVLNE	45 TGYSLFHKEKMILNE	45 TGYSLFHKEKMILNE	73 TGYGLFHKEKMLLNE	
P2	Block 3	10 RTNPSDNSSDSDAKS	27 RTNPSDNSSDSNTKT	28 SSDSNTKTYADLKHR	40 GAANPSDDSSDSDAK	72 DASDSDAKSYADLKH	
P3	N-term MAD20	02 KEKMVLNEGTSGTAV	03 EGTSGTAVTTSTPGS	13 VTTSTPGSKGSVASS	04 VTTSTPGSGGSVTSG	29 VTTSTPGSSGSVASG	
P4	N-term MAD20	11 VTTSTPGSSGSVTSG	25 VTTSTPGSKGSVTSG	14 SKGSVASSGSVASGG	05 SGGSVTSGGSGGSVA		
P5	Central MAD20	16 SGGSVTSGGSVTSGG	17 GGSVTSGGSGASVAS	26 SSGSVTSGGSVASVA	30 SSGSVASGGSVASVA	22 GSVTSVASVASVASV	
P6	Central MAD20	15 GGSGGSVASGGSVAS	18 GSGASVASVASVASV	32 SVASGGSVASGGSGN	23 SVASVASVASVASGG	07 ASVASGGSGGSVASG	
P7	C-term MAD20	06 GGSGGSVASVASGGS	31 GGSVASVASGGSGGS	19 SVASVASVASGGSGN	08 SGGSVASGGSGNSRR	12 ASVASGASGGSGNSR	
P8	C-term MAD20	24 VASVASGGSGNSRRT	20 VASGGSGNSRRTNPS	09 GGSGNSRRTNPSDNS	21 NSRRTNPSDNSSDSD		
P9	N-term RO33	33 KEKMVLKDGANTQVV	34 DGANTQVVAKPADAV	41 DGANTQVVAKPVPAV	43 DGANTQVVAKPAGAV	35 VAKPADAVSTQSAKN	
P10	C-term RO33	42 VAKPVPAVSTQSAKN	44 VAKPAGAVSTQSAKN	36 VSTQSAKNPPGATVP	37 NPPGATVPSGTASTK	38 PSGTASTKGAIRSPG	39 KGAIRSPGAANPSDD
P11	N-term K1	46 KEKMILNEEEITTKG	61 KEKMVLNEEEITTKG	74 KEKMLLNEEEITTKG	47 EEEITTKGASAQSGT	76 EEEITTKGASAQGSS	
P12	N-term K1	48 GASAQSGTSGTSGTS	62 GASAQSGASAQSGAS	77 GASAQGSSGPSGTPS	56 TSGTSGTSGTSGTSG	49 TSGTSGTSGPSGPSG	
P13	Central K1	67 TSGTSGPSGPSGTSP	75 TSGTSGPSGTSPSSR	57 GTSGTSAQSGTSGTS	65 GTSAPSGSGTSPSSR	80 GTSGPSGTGPSGTSP	
P14	Central K1	79 SGTSGPSGTSGPSGT	50 SGPSGPSGTSPSSRS	68 SGPSGTSPSSRSNTL	78 SGPSGTPSGTSGPSG	58 SAQSGTSGTSAQSGT	64 SAQSGPSGTSAPSGS
P15	C-term K1	63 QSGASATSAQSGPSG	59 TSGTSGTSGTSPSSR	51 GTSPSSRSNTLPRSN	69 PSSRSNTLPRSNTSS	52 SNTLPRSNTSSGASP	81 LPRSNTSSGASPPAD
P16	C-term K1	70 LPRSNTSSGAIPPAD	66 SNTSSGAPPADASDS	53 NTSSGASPPADASDS	71 SGAIPPADASDSDAK	82 SGASPPADASDSDAK	54 PPADASDSDAKSYAD

The 15-mer peptide sequence is represented in single letter code, and the location of the peptide in the region is indicated. Pools contained equimolar amounts of four to six biotinylated peptides (0.1 nM each).

**Figure 7:**
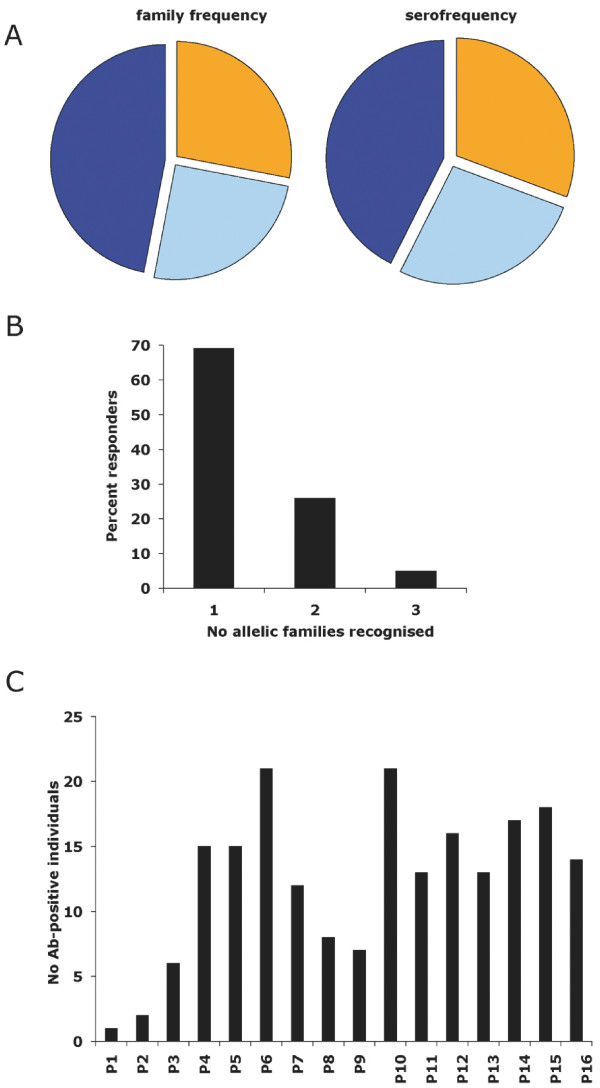
**Seroprevalence and specificity of anti-MSP1-block 2 IgG in Dielmo**. A) Seroprevalence to each family and family distribution within the parasite population. Seroprevalence was determined using sera collected during a cross-sectional survey conducted before the 1998 rainy season (on 2-3 August 1998) when 243 villagers (i.e. 95% of the village population) donated a fingerprick blood sample. The presence of anti-MSP1 block2 specific IgG was assessed by ELISA on 16 pools of biotinylated peptides (sequence and composition of the pools described in Table 5). Plasma reacting with one or more pool was considered seropositive, and grouped by family irrespective of the number of peptides sequences recognised within each of the three family types (i.e. MR alleles were disregarded as such, seropositivity being allocated either to Mad20 or to RO33). The relative distribution of family genotypes was established by nested PCR on 306 samples collected longitudinally during the 1990-9 time period as shown in Table 1. Colour codes K1: dark blue; Mad20: orange, RO33: light blue. B) Frequency of plasma with antibodies reacting with one, two and three allelic families. The number of families recognised is shown irrespective of the actual type recognised (i.e. individuals reacting with only K1-types, only Mad20-types or only RO33-types are placed together in the group reacting with one family). C) Frequency of reaction with each peptide pool.

The frequency of recognition of each allelic family mirrored the frequency distribution of the family types within the parasite population (Figure 7A). The antibody reaction was family-specific and usually restricted to one family, with 73%, 23% and only 4% of the positive plasma reacting with one, two and three allelic families, respectively (Figure 7B), consistent with our previous survey in this village [27]. Figure 7C shows that antibody response to pools 1 and 2, derived from the adjacent block 1 domain and block 3 respectively, was rare. No immunodominant region was identified within block2. Antibodies to the repeats were detected alongside antibodies to the family-specific N- or C-terminus block2 sequences. Interestingly, when scrutinizing the response at the individual peptide level in the context of the allelic sequence diversity within this village, it was clear that antibodies reacted with motifs displayed by the vast majority of the alleles observed in the village, namely either frequent/universal di-motifs (such as motif 31 in the K1 family) or family-specific unique sequences. Indeed, 24 of 26 villagers with antibodies to K1-type peptides reacted with sequences present in 74 or more of the 77 observed K1 alleles. Similarly, 16 of 16 responders to Mad20-type peptides reacted to sequences present in 32 or more of the 34 observed alleles.

In addition to the family-specific antibodies, some villagers had sequence-variant specific antibodies, namely reacted with only one of sibling peptides while others reacted with multiple sibling peptides displaying sequence variants. For example, within the group of sibling peptides derived from the N-terminus of Mad20 block2 (peptides #04, 13, 25, 11 and 29), some villagers reacted with one peptide (#29), whilst others reacted with two (#29 and 04 or 29 or 11), but none reacted with all five peptides. Likewise for the group of sibling peptides derived from the K1 block1/block2 junction (peptides #46, 61 and 74), some villagers reacted with one (#61), two (#61 and 74) or all three peptides. This suggests that sequence variation indeed translates into antigenic polymorphism. Whether antibody reaction with multiple sequence variants reflects serologic cross-reaction or accumulation of distinct antibody specificities is unclear.

### Antibodies to MSP1 block2 and subsequent clinical malaria

To look for a putative association between the presence of anti-MSP1 block2 antibodies and protection against clinical malaria during the subsequent high transmission season, we mined the database for the occurrence of clinical attacks within the 5-month period following the 1998 cross-sectional blood sampling studied above. The 243 individuals experienced a total of 266 clinical malaria attacks (mean = 1.09, 95%CI: 0.88-1.30). The number of clinical malaria attacks experienced per individual varied from 0 (140 individuals) to 7 (1 individual). Recordings of the entomological inoculation rate indicated a mean of 170 infected bites/person during this time period. Twenty-nine percent of the seronegative individuals (with no detected anti-MSP1 block2 antibodies) experienced a clinical attack during that period, compared with 15% of individuals with anti-block2 antibodies. Using a Poisson regression model, the crude estimates of the Incidence Rate Ratio (IRR) of malaria attacks associated with the presence of antibodies to one allelic family or ≥ 2 families (no antibodies as reference group) were 0.55 (95%CI: 0.38-0.80) and 0.21 (95%CI: 0.08-0.58), respectively (P < 0.0001). In a multivariate Poisson regression analysis, this association was independent of haemoglobin type or ethnic group. However, it was confounded by age, *i.e*. within the age groups, there was no significant association between the incidence of clinical malaria attacks and the number of MSP1 block2 allelic families recognized.

### Analysis of the response during a high transmission season

To study the impact of novel infections during the transmission season on the humoral response to MSP1 block2, we investigated the fingerprick blood samples collected from 25 seropositive individuals throughout the high transmission season.

**Figure 8:**
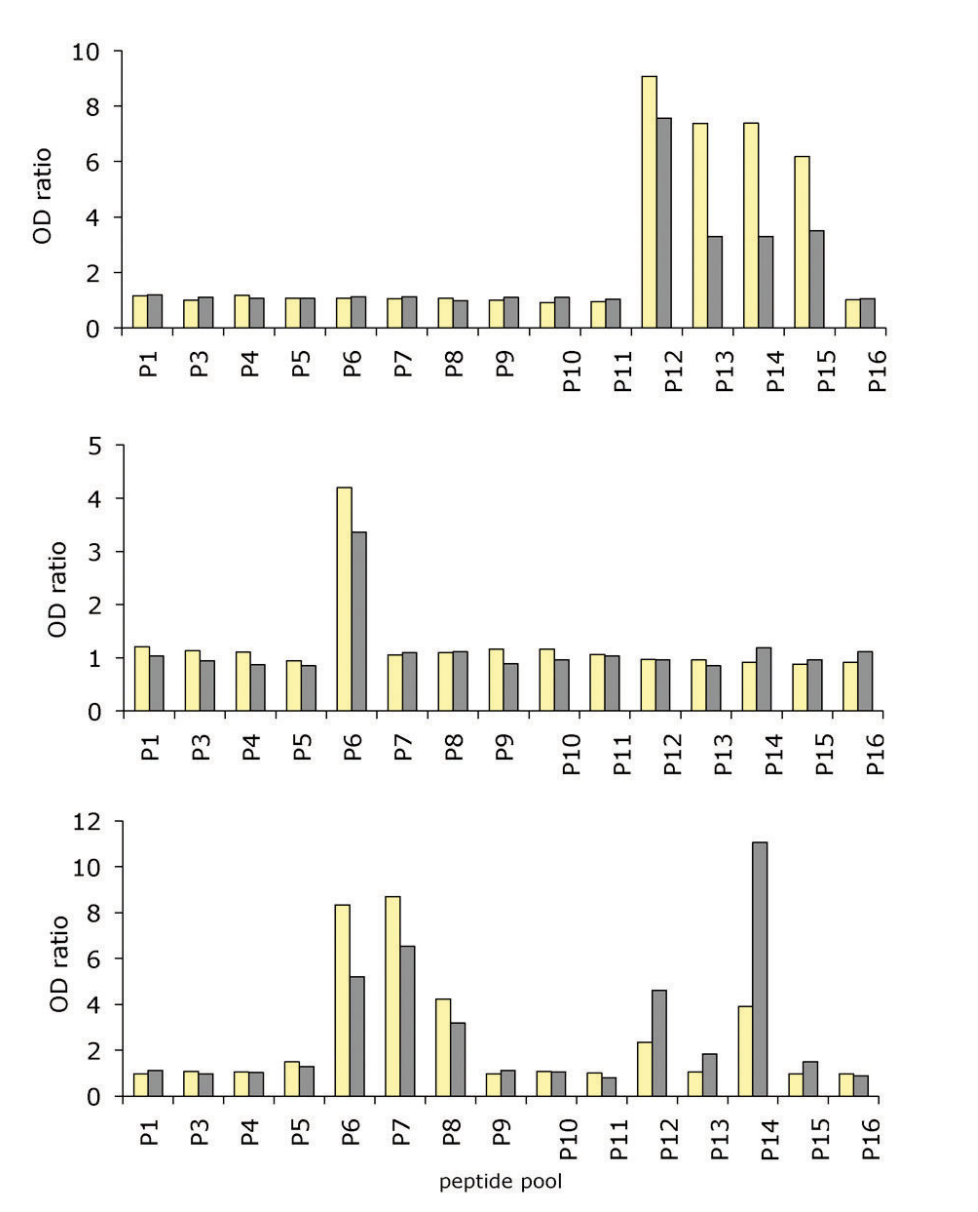
**Typical profiles of the temporal evolution of MSP1 block2- specific IgG before and after the 1998 rainy season**. Antibodies were assayed from 25 individuals in August 1998 (yellow) and December 1998, i.e. after a rainy season when each inhabitant was exposed to a mean of 170 infected bites. Anti-MSP1 block2 specific IgG was assessed by ELISA on 16 pools of biotinylated peptides. The upper, central and lower panels show a representative example of a reduction of specific antibodies, an essentially unchanged profile, and a boosting of pre-existing responses, respectively.

By the end of December 1998, namely five months after the cross-sectional sampling, the anti-MSP1 block2 antibody level was reduced by ≥ 2-fold in 15 subjects (59%), had varied less than 2-fold in 9 individuals (36%) (typical profiles are shown in Figure 8 upper and middle panel, respectively) and was ≥ 2-fold higher in one individual (Figure 8, lower panel). Importantly, when a change was observed, it concerned the intensity of the reaction but not its specificity. In other words, responding individuals usually reacted with the same pool(s) and within the pool(s) with the same individual peptide(s) before and after the transmission season. In none of the studied individuals were novel antibody specificities stably acquired during that time period, despite an elevated infection rate.

**Figure 9:**
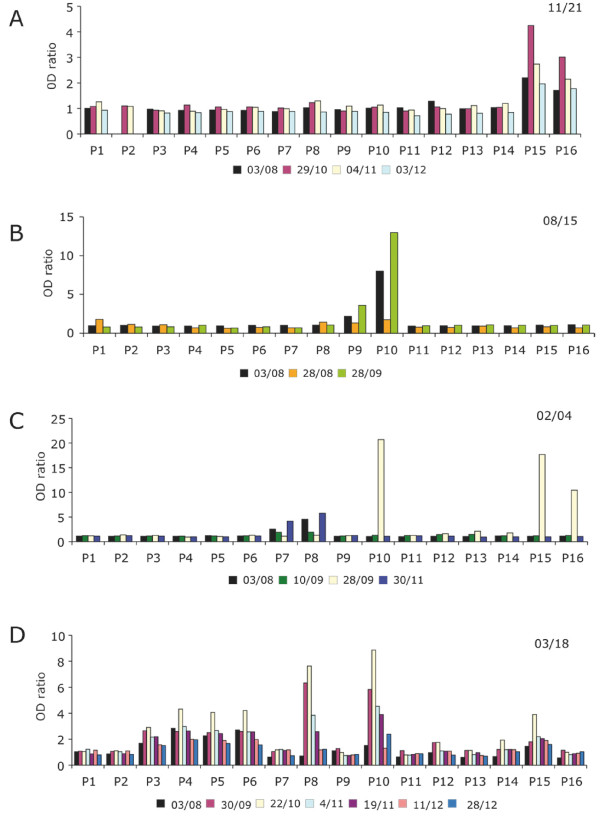
**Temporal fluctuation of MSP1 block2- specific IgG during the 1998 rainy season**. Antibodies were assayed on 16 pools of biotinylated peptides (sequence and composition of the pools described in Table 5). Typical individual patterns are shown, with the dates of blood sampling shown on each graph. A) Transient boosting of a pre-existing response in a 14 y old subject (code 11/21), who had a clinical malaria attack on 29/10/98. B) Transient loss of a pre-existing response in a 5 y old child (code 8/15), who had a clinical malaria attack on 28/08/98. C) Transient acquisition of a novel specificity in a 9.5 y old child (code 02/04), who had a clinical malaria on 10/09/98. D) Transient changes in a 5 y old child (code 03/18), who experienced three successive clinical episodes during that time period on 17/09/98, 22/10/98 and 11/12/98. For each cinical episode, an antimalarial treatment was administered to the patient on the day of diagnosis.

We then carried out a follow up of the antibody responses in villagers who experienced clinical malaria during the 5-month transmission season, using archived fingerprick sera collected monthly, and when available, sera on the day of the clinical malaria episode. Transient fluctuations were observed, with in some cases boosting of a pre-existing response (see a representative example in Figure 9A), in others a decrease in antibodies (*idem *Figure 9B) or evidence of a short-lived response (*idem *Figure 9C). This was also observed in children experiencing multiple clinical episodes during that same time period (*idem *Figure 9D). In nine out of 10 subjects in whom peripheral blood parasites collected at diagnosis of the clinical malaria episode were genotyped, the three allelic families were detected, and one individual harboured only 2 allelic families. In all 10 cases, infection with an allele against which there was no evidenced pre-existing response did not elicit any long lasting novel antibody specificity.

### Long term temporal analysis of the response to MSP1-block2

**Figure 10:**
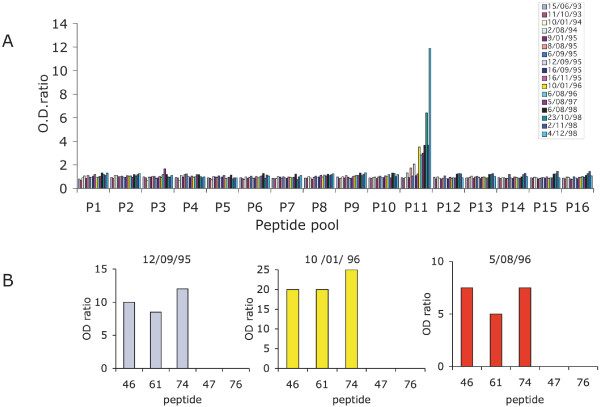
**Serological longitudinal follow up of child 01/13 from 6 months to 6 years of age**. Antibodies were assayed on 16 pools of biotinylated peptides (A) and to each individual peptide from positive pool 11 (B). The peptide sequence and composition of the pools are described in Table 5. The dates of blood sampling are shown to the right of the graph. A. reactivity on the peptide pool. B. reactivity of three representative blood samples on individual peptides from pool 11.

To analyse antibody patterns over several years, we used archived systematic blood samples collected during the longitudinal survey. Confirming a previous study in this village [27], once acquired, the response to MSP1-block2 was essentially fixed over time. A typical example is shown in Figure 10, where a 6-year follow-up was carried out on child 01/13, starting at 6 months of age. The child had been exposed to a mean of 200 infected bites each year over the six years. A single peptide pool was recognised by this child from the age of 2.5 years onwards (Figure 10A). The intensity of the signal fluctuated subsequently, including a drop during malaria attacks [e.g. the 2/11/98 blood sample, which was collected on the day a malaria episode was diagnosed presented a lower signal than the preceding (23/10/98) and following (4/12/98) samples], but nevertheless there was a progressive increase with cumulated exposure. Analysis of fine specificity on the individual constituents of peptide pool 11 showed the same pattern for all positive samples collected from this child with recognition of peptides # 46, 61 and 74, namely of the K1-specific block1-block2 junction (Figure 10B). The occurrence of clinical malaria episodes in this child resulted in temporarily reduced signals (hence antibody levels), but was not associated with stable acquisition of any novel specificity.

## Discussion

This first detailed longitudinal survey of *Pfmsp1 *block2 sequence polymorphism along with the assessment of the specific humoral response within a single endemic setting provides novel insights on the locus at the population level and on the possible selective forces underpinning such a polymorphism. A very large local polymorphism was detected, mainly due to microsatellite type variation, resulting in a very large number of low frequency alleles. Numerous novel alleles were identified here, including novel MR alleles, illustrating the value of in depth analysis of local polymorphism. The humoral response of the villagers, as deduced from the reaction with a series of 15-mer peptides, displayed features that illuminate its possible role in selection for diversity. The relative distribution of the family-specific antibody responses mirrored the relative distribution of the family types at the parasite population level. Seroprevalence was moderate. Responses were usually limited to a single family and frequently directed to family-specific sequences present in most of the alleles from that family circulating in the village. This is consistent with a frequency-dependent selection operating at the family level. However, the serological analysis did not outline frequent occurrence of immune responses possibly selecting for sequence variants within that family. It confirmed and expanded on previous observations in this setting [27] of an essentially fixed antibody specificity, despite intense exposure to a very large number of allelic types. Overall, the data point to a possibly antibody-driven diversifying selection maintaining balanced family types within the population, as proposed by other groups [3,12,23,24,28,33] but do not support the commonly accepted notion that the families accumulate mutations that allow the parasite to circumvent the host's capacity to build up an efficient immune response selecting for sequence variants.

The study design used here differs from previous studies in combining assessment of actual sequence polymorphism with analysis of sequence-specific immune response rather than combining PCR fragment size polymorphism with assessment of antibody responses using recombinant antigens [3,23,25,28,31-33]. No significant temporal fluctuations of the relative distribution of the allelic families was found over the 10-year period investigated, consistent with longitudinal studies in The Gambia using monoclonal antibody serotyping [42], and in Vietnam using PCR-based genotying [20], differing in this regard from studies conducted in Brazil [28,43]. The family distribution obtained here for symptomatic, high density infections was superimposable with the distribution observed in previous cross-sectional surveys of asymptomatic infections [44] [see Additional file 11]. Sequencing showed a very large number of low frequency genetic variants, along with one dominant allele (RD0) and few intermediate frequency alleles (DK65, RD5, DM11). Only 29 out of 126 alleles were detected at a frequency above 1%. The level of polymorphism of the non repeated R033 family was similar to the level observed in the same setting for *Pfmsp4*, in however a much smaller (30-fold lower) sample size [45]. Tests for neutrality did not show a significant departure from neutrality, for the repeated domains of the K1-, Mad20- and MR- types and for the repeatless RO33 family. The Tajima's test for RO33 is consistent with selectively neutral mutations [46]. Testing the repetitive sequences for selection is difficult, since the mutational and evolutionary processes underlying their diversification are not clearly understood. The Ewens-Watterson (E-W) [38] test is based on the idea that, under neutrality, the observed number of alleles should be consistent with the observed gene diversity. Because of their particular mutation patterns and rates, neutral microsatellites tend to show naturally more alleles than expected from their observed gene diversity [47]. This phenomenon could artificially reduce the effect of balancing selection on allele distribution and as such reduce our ability to detect it. However, the effect of repeated mutations on the distribution of alleles is most of the time rather small and occurs mainly when the observed gene diversity is low which is not the case for MSP1 repeat domains [47]. Hence, if a strong balancing selection is acting on the MSP1 repetitive sequences, we should still be able to detect it. Furthermore, the reported evidence for diversifying selection on the *Pfmsp1 *block2 locus [3] included the analysis of such repeat-related polymorphisms. When considering fragment size polymorphism, there was no evidence of departure from neutrality either, contrasting with a recent report from Kenya [16], where a different parasite population sampling strategy was used. The 306 samples successfully genotyped here originated from 229 different villagers (approx. 85% of the village inhabitants, with all age groups included) over a decade, whereas the 362 samples analysed in Kenya were collected by repeated sampling from 45 infants during a 4y period [16]. Such repeated sampling from the same sub-group may have biased the analysis of population polymorphism, in particular as successive clinical malaria attacks experienced by a child are each caused by "novel" parasite genotypes [48].

To assess the consequence of sequence diversity on antigenicity, and in the search for evidence of antibody-driven diversifying selection, we opted here for the use of synthetic peptides encompassing a large number of sequence variants, rather than using recombinant proteins expressing an entire MSP1 block2 domain, which exposes multiple antigenic determinants. Whereas recombinant proteins allow to study family cross-reactivity, recognition at the single epitope level is best monitored using synthetic peptides. Individual MSP1 epitopes are displayed by short peptidic sequences, which are recognised by monoclonal antibodies [15] and human sera [15,26,27]. Use of synthetic peptides may result in underrepresenting certain epitopes, including conformational epitopes, and hence in underestimating the overall seroprevalence to the locus. However, interestingly this assessment using synthetic peptides outlined a strikingly similar relative distribution of family genotypes and family-specific antibodies in Dielmo, consistent with observations in other settings monitoring immune responses using recombinant proteins [3,23,25,28,29,31-33,36].

The humoral response of the Dielmo villagers suggested a family-specific selection pressure rather than an antibody-mediated selection for sequence variants. Seroprevalence increased with age, but the number of peptides recognised was unrelated to age. Most individuals had antibodies to one family only, and within that family, polymorphic sites as well as common repeat motifs and the more conserved family-specific sequences were recognised. Importantly, antibody specificity remained essentially fixed over time. Confirming previous observations in this setting [27], the long term longitudinal follow up showed that cumulated exposure to an increasing number of *Pfmsp1 *block2 alleles was usually not associated with stable acquisition of antibody specificities to additional sequence variants. Analysis of anti-MSP1 block2 responses during a transmission season showed that some individuals experiencing a high density clinical episode had their pre-existing responses boosted, while antibodies were transiently undetectable in other patients. In some cases, novel specificities were acquired only transiently, since they were rarely detected a few weeks after the episode and undetected in subsequent longitudinal samplings, where a steady state, essentially stable specificity profile was consistently observed. The response pattern to MSP1 block2 markedly differs from the progressively enlarging antibody repertoire to erythrocyte surface variant antigens (see [49] and references therein). This rather stable steady state specificity profile is highly reminiscent of clonal imprinting. It may reflect particular constraints on the response or stimulation by chronic asymptomatic carriage and/or novel infections, quite frequent in such a holoendemic setting. Clonal imprinting of responses to another *P. falciparum *merozoite surface antigen displaying variable repeats, namely MSP2 has been suggested in some studies [50,51], but was not supported by studies on PfMSP1block2 responses in a hypoendemic Sudanese setting [25]. The best evidence in favour of clonal imprinting in malaria parasites stems from studies on cellular responses to peptide variants of the CS protein [52].

Studies conducted in other African settings, using recombinant proteins, have outlined several features that are consistent with the observations we made in Dielmo: i) a moderate seroprevalence to MSP1 block2 that increases with age [3,24], ii) recognition of a single family by a large proportion of responders [3,25,30], iii) family-specific and sub-type specific responses [3,23-25] along with recognition of conserved family-specific flanking domains [23,24]; iv) transient acquisition antibody specificity or loss of pre-existing response during a malaria attack [24,25]. Thus in other African settings as well, the MSP1 block2-specific humoral response is unlikely to exert a significant selection favouring the outgrowth of parasites presenting mutant epitopes. This does not rule out a selection by cellular immune effectors, which has not been assessed here. This deserves a detailed study, since sequence variation of the block1-block2 junction has been shown to influence cellular responses [53].

Confirming studies in other areas [3,23,24], the antibodies to one or more MSP1 block2 allelic families were prospectively associated with protection against subsequent clinical attacks. However, multivariate analysis showed this association to be confounded by age, and as such difficult to distinguish from concomitant acquisition by Dielmo villagers of other responses involved in protection. Protection against clinical malaria has been indeed associated with an array of antigens in various endemic settings, including the antigenic variant PfEMP1 exposed onto the infected red blood cell surface [54,55], msp1-19 [56], R23 [57], msp3 [58].

Apart from the RO33 types, the large sequence polymorphism observed in Dielmo was essentially of microsatellite type. Variations within the K1, Mad20 and MR families mainly focused on the second and third codon of the tripeptide repeats, involving, furthermore, a restricted set of amino acid residues. As noted by others [16], fragment length did not adequately describe the local genetic diversity. Based on size polymorphism, 55 alleles were identified, but 126 alleles were identified by sequence analysis. All six RO33 alleles had the same size. Some size bins used to group alleles from other families turned out to group a large number of distinct sequences (up to 11 for K1-types and up to 9 for Mad20 types) [see Additional file 12].

Sequence analysis identified numerous novel alleles and specific motif arrangements, with 113 of the 126 *Pfmsp1 *block2 allele sequences observed in Dielmo being novel. The RO33 types displayed novel point mutation polymorphisms. Compared to the reported sequences, the K1 alleles from Dielmo were more diverse (higher number of distinct motifs), with more frequent usage of motifs 3 and 4, and with a novel K1-type motif encoding the SVT tripeptide (7). The Mad20 types were longer (more repeats per allele), used a restricted set of codons and particular motifs, with a higher occurrence of SGG-encoding motifs, more frequent use of motif 8 and fewer motifs 7 and 4. The MR family accounted for up to 13.3% of all *Pfmsp1 *block2 alleles from Dielmo, a lower frequency than the 28-29% observed in a Kenyan holoendemic setting [11,16]. We could not identify any epidemiological parameter associated with the presence of MR alleles: there was no association with age, gender, ABO or Rhesus blood group. Interestingly, like the other three families, MR alleles from Dielmo presented specific characteristics. All harboured a RD5-type RO33 moiety, differing from most MR alleles with a worldwide distribution [11,16]. Furthermore, DMR1 displayed a novel MR subtype with a 5 7 5 motif (Mad20 sub-group 1c) instead of a 8 7 5 motif (Mad20 sub-group 2c). In addition, a novel hybrid with a 3' RO33/K1 hybrid sequence was observed. Whether this DMRK allele was generated by insertion of a SPPADA-encoding DNA segment within a MR allele (possibly MR6), or whether this element was inserted within RD5 before recombination with a Mad20 allele is unclear. Insertion of the SPPADA-encoding segment within any allele of the RO33 family has never been reported, but was observed within the K1-type in this study (allele DK67) and in other settings [9]. Observation of a single RO33 progenitor together with a single Mad20 progenitor led Takala et al [16] to propose that the MR family arose from a single recombination event. The present data rather suggest that several separate recombination events involving distinct RO33-types and Mad20-types progenitors have contributed to the generation of this hybrid family.

The characteristic of the *Pfmsp1 *block2 allelic repertoire in Dielmo is in line with the epidemiological conditions prevailing in the village. Unlike the surroundings where transmission is moderate and highly seasonal, transmission in Dielmo is perennial and intense [59]. Therefore, local transmission largely dominates over the import of alleles from the neighbouring area during the 9-10 months of the dry season. As such, Dielmo constitutes a transmission area where a high level of genetic diversity can be maintained. Detailed analysis of parasite population structuring and expansion in this setting awaits study of additional genetic loci. Transmission in the village occurs throughout the year, albeit with marked seasonal fluctuation in entomological inoculation rates and vector species [59]. The seasonal pattern of family distribution may reflect different fitness/survival rates associated with different allelic families under different transmission conditions and/or for different *Anopheline *vector species. Additional studies are needed to explore this hypothesis further.

Previous studies have surveyed sequence polymorphism across large geographic areas or with a small sample size in a single setting, and as such did not capture the micro-geographic features observed here in a single setting. Better understanding at micro-geographic level is essential to analyse immune responses in the context of the parasite population to which people are exposed. This is critical importance to interpret selective forces on parasite population, and to design rationale control measures accordingly.

## Conclusion

The *Pfmsp1 *block2 locus presents a population sequence diversity larger than we could anticipate from published studies. A very large local polymorphism was detected, mainly of microsatellite type. The humoral response observed here using synthetic peptides was consistent with a frequency-dependent selection operating at the family level. However, there was no evidence for major humoral selection for sequence variants. In contrast, antibody specificity remained fixed over time, despite exposure to novel allelic forms. Such a lack of stable acquisition of novel antibody specificities in response to novel infecting types is reminiscent of clonal imprinting. The locus appears under antibody-mediated diversifying selection in a variable environment that maintains a balance between the various family types without selecting for sequence variant allelic forms. At the family level, intra-family sequence diversity is consistent with a neutral evolution and with the observed characteristics of the antibody response. Finally, the data reported here do not confirm the association of the acquired humoral response to MSP1 block 2 with protection against subsequent clinical P. *falciparum *malaria attacks.

## Methods

### Study site and patient recruitment

Dielmo, located in Sine Saloum, Senegal, is a village of approximately 250 inhabitants, where malaria is holoendemic. In 1990, the entire village population was enrolled in a longitudinal prospective study described in detail elsewhere [60]. The main vectors in the village are *Anopheles gambiae s.s*. and *An. funestus *[59]. Informed consent was obtained from each adult participant and from parents or legal guardians of each child at the beginning of the study and was renewed on a yearly basis. Individuals could withdraw from the study at any time. Each year the project was reviewed and approved by the Joint Ministry of Health and Pasteur Institute Surveillance Committee. The retrospective analysis has received ethical clearance from the National Ethical Committee of the Republic of Senegal.

### Parasite samples

We studied here 336 samples collected from mild malaria episodes selected from the existing collection of frozen blood samples and analysed for drug resistance markers [61]. The sampling strategy was as follows: From a list of approx 3,400 samples collected longitudinally during a malaria episode, samples were chosen for molecular analysis so as to survey the largest possible panel of villagers. Since in this hyperendemic setting the heaviest clinical malaria burden is in the <10 y olds and since some children are more susceptible than others [62], we needed to avoid iteration bias due to the increased susceptibility of some individuals. This reduced the risk not only of over-representing certain genotypes to which some individuals might be more susceptible than others, but also of overestimating polymorphism, because each of the successive clinical malaria attacks experienced by one person is caused by "novel" parasites [48]. We therefore set an interval of >3 years between two samples from the same individual, with the further restriction that no person could contribute with more than three samples in all. The number of samples studied each year is indicated in Table 1. Of the 336 samples selected, 306 were genotyped for *Pfmsp1 *block2. They originated from 229 villagers, with 159, 63 and 7 villagers contributing once, twice and three times, respectively, to the panel of *Pfmsp1 *block2-positive samples studied here. This included 120 males and 109 females [159 and 147 males and females, respectively, among the panel of samples studied]. The mean age ± standard deviation at the time of blood sampling was 11.5 ± 13.36 years (minimum = 0.3 y, maximum = 89.7 y, median = 7.3 y, Q25-Q75 = 3-13.9 y). [Mean age of males 12.08 y median age 6.9 y; mean age of females 10.82 y; median 7.9 y]. There were 275, 5, 29 and 1 samples from villagers with an AA, AC, AS and SS haemoglobin type (six of unknown type). The samples were from 31 out of the 34 village compounds.

### *Pfmsp1 *block2 genotyping by nested PCR

Frozen blood samples were thawed and extracted with phenol-chloroform [48] and stored at -20°C until use. *Pfmsp*1 block2 was amplified by semi-nested PCR in a 50 μL reaction volume containing 5 μL DNA, 50 mM KCl, 1.5 mM MgCl_2_, 10 mM Tris-HCl pH 9.0, 200 μM dNTP, 5 U Taq Polymerase (Amersham Pharmacia), 1 μM of each primer. For the first PCR, the conserved primers used were Fmsp1uf 5'GAAGATGCAGTATTGACAGG and Fmsp1ur 5'CATTAATTTCTTCATATCCATC. A first denaturation step at 96°C for 5 min was used, followed by 25 cycles of denaturation at 96°C for 1 min; hybridization at 64°C for 90 sec, extension at 72°C for 45 sec with a final extension at 72°C for 7 min. The semi-nested PCR was carried out using a forward family specific primer and the reverse conserved primer Fmsp1ur under the same conditions for 25 cycles except that the annealing was done at 68°C. The sequence of the forward family-specific primers was k1ff 5'ATGAAGAAGAAATTACTACAAAA, mad20ff 5'GAAGGAACAAGTGGAACAGC and ro33ff 5'TACTCAAGTTGTTGCAAAGC. PCR products were analysed on 1.5% Nusieve:agarose gels (1:3). The size of the bands was evaluated using a 100 bp DNA ladder (Bio-Rad) as size markers. Alleles were classified in 10 bp bins. A *Pfmsp1 *block2 genotype could be generated for 306 of the 336 samples. Of the 30 negative samples, one had a poor DNA quality (negative PCR for five loci tested), but the other 29 generated PCR products for other loci (*Pfcrt*, *Pfdhfr-ts *and microsatellite loci). Whether the failure to amplify *Pfmsp1 *block2 was due to polymorphism within the primer sequence or a lower sensitivity of the reaction as compared to the other loci is unknown. These DNAs were excluded from the analysis.

In the case of mixed infections where different alleles belonging to the same family were detected by size polymorphism, the bands of different size were excised from the agarose gel, re-amplified with specific primers to recheck the allele type.

### Sequencing

PCR products obtained by semi-nested PCR using family specific forward primers were directly sequenced. All *Pfmsp1 *block2-derived PCR products were purified using polyacrylamide P-100 gel (Bio-Gel, Bio-Rad, 150-4174) on 96 well plates equipped with a 0.45 μm filter (96 well format, Millipore,1887, ref MAHVN4550). The purified product was quantitated by comparing it with DNA quantitation standards (Abgene^® ^QSK-101) after electrophoresis on 1.2% agarose gel. The sequencing reaction contained 2 μl of PCR product (≥ 20 ng), 1.25 μL 5× Buffer, 1.5 μL BigDye v3.1, 2 μL of 2 μM primer in a 10 μL final volume. Amplification was performed in a GeneAmp9700 (Applied Biosystem) [1 min at 94°C followed by 35 cycles of (10 sec at 96°C, 5 sec at 50°C and 4 min at 60°C), and held at 4°C. The products were then precipitated and sequenced on both strands using an ABI^® ^prism 3100 DNA analyzer as described [61]. There were a few cases where sequencing of the excised band proved not possible because of ambiguity in base calling, probably reflecting mixture of alleles with similar size. These samples were discarded from the analysis. We retained in the analysis only sequences where base calling was non ambiguous and the signal accounted for more than 95% of the signal for each individual base.

False recombinant alleles can be generated during PCR as a result of template switching, when long amplicons are generated, namely *Pfmsp1 *blocks 2-6, with cross-over sites identified in the distal part of block 3 and in block 5 [63]. To reduce the risk of this potential pitfall, short regions were amplified (i.e. upstream from the identified cross-over sites), with PCR anchored in conserved regions but relatively close to the junction with polymorphic sequences. Second, we verified absence of undesired amplification from deliberate mixture of reference alleles using the semi-nested PCR strategy The PCR fragments were sequenced and all were perfect match with the reported sequence.

Accession numbers are as follows: [Genbank: EU032016-EU032159, EU032160-EU032227, EU032227-EU032246, EU037095, EU032250-EU032276 and EU032248] for the DK, DM, RD0, RD1-5, DMR and DMRK sequences, respectively.

### Sequence analysis

*Pfmsp1 *block2 alleles deposited in Genbank were retrieved by repeated blasting using each individual 9-mer nucleotide sequence observed in K1-type or Mad20-type alleles and the full length RO33-type block2 sequence. In addition, K1 alleles reported by Tetteh et al [15] originating from Zambia were included. The curation indicated by Miller et al [8] was included when needed. The various alleles were aligned using ClustalW and curated manually. Redundant alleles were discarded. This resulted in overall 59 distinct K1-type [see Additional file 5], 52 Mad20-type [see Additional file 6], four RO33-type [see Additional file 3] and nine MR-type alleles [see Additional file 7]. The alleles from Dielmo were compared to the reported alleles for the structure of the microsatellites: frequency of the individual tripeptide motifs, overall number of repeats, numbers of each individual tripeptide and combinations thereof (dimers, trimers and tetramers).

### Neutrality tests

Allele distribution was analysed using the Ewens-Watterson-Slatkin (EWS) tests [38,39]. The test was applied considering a family as a single allele (i.e. grouping all alleles from that family together) or by considering individual alleles within each family independently. Individual alleles were then classified 1) by size and nucleotide sequence polymorphism or 2) by size polymorphism alone. Ewens-Watterson tests were performed using the software *Pypop *[64]. Nucleotide diversity within the RO33 family was analysed using Tajima's *D *test [40] and Fu and Li's test [41] from DnaSP version 4.0 software developed by Rozas et al [65].

### Serological analysis

Archived sera, collected throughout the longitudinal follow up were used. Seroprevalence was studied using 243 plasma (i.e. 95% of the village population) collected during a cross-sectional survey conducted on 2-3 August 1998 at the beginning of the rainy season (27, 25, 26, 40 46 and 79 in the 0-2 y, 3-5, 6-8, 9-14, 15-24 and ≥25 y age groups, respectively). A subset of 25 sera collected in December 1998 from individuals whose August 1998 scored positive for antibodies to one or more MSP1-block2 derived peptides was analysed. A follow up of ten individuals during the 1998 rainy season was carried out using the monthly fingerprick blood samples collected on a systematic basis together with a fingerprick sample collected on diagnosis of clinical malaria when available. The entomological inoculation rate during the August-December 1998 period, assessed as described [59], was 170 infected bites/person. In addition, archived sera from children, collected longitudinally during the survey were used to follow the acquisition of antibodies over a period of several years.

A set of 82 15-mers derived from MSP1 block2 tripeptide repeats and the family-specific flanking region was synthesized by Chiron Mimotopes Pty. Ltd. (Clayton, Victoria, 3168, Australia). There were 34, 31, and 12 K1- Mad20- and RO33-specific sequences. In addition, 5 peptides derived from the junction with block1 were used. The peptide sequences are described in Table 5. The peptides represented the tripeptide combinations observed in Dielmo for the K1 and Mad20 families [see Additional file 9]. These peptides were synthesized with an N-terminal biotin group separated from the peptide sequence by a SGSG spacer and with an amidated C-terminus. All peptides were soluble. A similar set of peptides was used to explore the humoral response in Dielmo villagers in previous studies [26,27]. Based on these results, which showed a restricted specificity, and in view of the limited volume available for several sera, we first screened individual sera using 16 peptide pools (4-6 peptides per pool as described in Table 5) and in a second step analysed the reactivity of the positive sera on individual peptides from each positive pool. ELISA was performed on streptavidin-coated plates with either pools of 0.1 nM each biotinylated peptides or 0.5 nM biotinylated peptide adsorbed in each well as described [27]. We checked with control mouse sera and individual human positive controls that peptide dilution within the pool of peptides did not modify the outcome of specificity analysis. Human plasma was tested in duplicate at a 1:500 dilution and bound IgG or IgM was measured using horseradish peroxidase-conjugated goat F(ab')2 to human IgG Fc (γ) or to human IgM Fc (μ) (Cappel, Organon-Technica, Turnhout, Belgium). Optical density (OD) was measured on an Emax reader (Molecular Device) at 450 nm. Control wells without peptide were used to check for potential anti-streptavidin antibodies. The wells that gave a signal twice the OD value of the wells without peptide were considered positive. IgG subclass analysis was performed as described [27].

### Association with protection

This was done based on the data gathered during the longitudinal survey protocol and available in the database. Daily clinical surveillance was carried out over the August-December 1998 follow-up period, as described [60,66]. Each villager was visited at home for clinical surveillance and blood films were made in case of fever. The protocol included the notification of all febrile episodes to the medical staff and the controlled use of anti-malarial drugs. A malaria attack was defined as an association of symptoms suggesting malaria with parasitaemia above an age-specific threshold as described [66,67]. An anti-malarial drug cure was administered by the medical staff in all cases of malaria attacks. Procedures to estimate association with protection have been described [56,57,68]. In brief, the number of clinical malaria attacks experienced during the follow-up was analyzed as the dependent variable in a Poisson regression model, using the number of days of presence in the village as exposure variable. The association between the incidence of clinical malaria attacks and independent variables, i.e. presence of antibodies to allelic families, age, haemoglobin type or ethnic group, was tested.

### Statistical analysis

Yearly distribution of the 524 PCR fragments by allelic family was analysed by Pearson Chi2 with the assumption that the alleles co-infecting the same individual were independent. Allelic family distribution by gender, age, Hb type, ABO group, Rhesus group and by month was analysed by Fisher's exact test. The allelic family infection rate (percentage of infected individuals harbouring one or more alleles from that family) by gender, β-globin type, ABO or Rhesus blood group, by age (0-1 y, 2-5 y, 6-9 y, 10-19 y and ≥20 y) and by season in the year was analysed by Fisher's exact test. For the analysis of seasonality, the year was divided into three periods based on the rains, the vectors present and the entomological inoculation rate. The mean entomological inoculation rate was 32, 140 and 39 infected bites/person/year in February-May (dry season), June-October (rainy season), and November-January, respectively.

The estimated multiplicity of infection was first analysed using a zero-truncated Poisson regression model, with the assumption of a constant probability to detect an additional allele in a homogeneous carrier population. The mean predicted estimated moi was 1.193 allele/infected individual. The predicted distribution was calculated, grouping the classes with estimated moi ≥ 4 and did not differ from the observed one (51.6% vs. 51.9%, 29.4% vs. 31%, 15.0% vs. 12.3%, 3.9% vs. 3.7% for observed vs. predicted estimated moi 1, 2, 3 and ≥4, respectively (Chi2 test, 3 df ≥ 2.53, p = 0.47). Estimated moi distribution by age group (0-1 y, 2-5 y, 6-9 y, 10-19 y and ≥20 y), gender, Hb type, ABO group, Rhesus blood group, year, month of the year and season was analysed by non parametric Kruskal-Wallis test.

## List of abbreviations used

MSP1: merozoite surface protein 1.

## Authors' contributions

OMP designed the study. NN and JP established the experimental conditions for *Pfmsp1 *block2 amplification and sequencing. NN carried out sequencing with the help of MTE and CB. OMP and NN conducted the genotyping analysis, database mining and curation/analysis. HJ carried out the serological assessment. AT, LM CS, JFT and CR conducted the epidemiological and clinical work and the sample collection. OMP, NN, HJ and CR analysed the data. FP and JO analysed the population structure and diversity, CR conducted the statistical analysis. OMP wrote the manuscript with input from NN, FP, HJ and CR. All authors read and approved the final manuscript.

## Supplementary Material

Additional file 1 **Distribution frequency of *Pfmsp1 *block2 fragment size in Dielmo, Senegal**. This file shows the frequency of alleles by fragment size for the K1 and MAD20 types (the RO33 alleles did not display fragment size polymorphism). The locus was amplified by semi-nested PCR and PCR products were analysed on 1.5% Nusieve:agarose gels (1:3) and visualised by ethidium bromide staining. The size of the bands was evaluated using a 100 bp DNA ladder (BioRad) as size markers. Alleles were classified in 10 bp bins. Click here for file

Additional file 2 **Temporal distribution of *Pfmsp1 *block2 allelic families as assessed by nested PCR and sequencing**. This file shows the relative distribution of the various allelic families by year as assessed either by PCR genotyping or gene sequencing. The number of samples genotyped and the number of sequences generated for each calendar year are indicated in Table 1. Sequences were determined from single PCR bands generated by family-specific nested PCR. Each sample was tested in three parallel PCR reactions triggered by one forward family specific primer and a reverse universal primer. Only the reactions generating a single band (estimated by size on agarose gels) were processed for sequencing. Click here for file

Additional file 3 ***Pfmsp1 *block2 RO33-types deposited in the Genbank database**. This file lists the Genbank accession number of the deposited RO33-type alleles, along with the country of origin of the samples, and the sequence in single amino acid code. For references see the main text. Click here for file

Additional file 4 **Sequence analysis of the Dielmo alleles and comparison with the alleles reported in the literature and in the databases**. This file provides a detailed analysis of the molecular variation of the repeat motifs (number, sequence and arrangement) and of the point mutations observed in the various alleles from Dielmo and a comparative analysis with the alleles deposited in Genbank. Click here for file

Additional file 5 ***Pfmsp1 *block2 K1-types deposited in the Genbank database or published in the literature**. This file lists the Genbank accession number of the deposited K1-type alleles, along with the repeat motifs coded as indicated. 59 distinct alleles were identified, numbered 1-59. Several alleles have been observed in multiple settings and/or on multiple occasions. The geographic origin is shown, when indicated in the deposited sequence or in the corresponding publication. The codes used for the tripeptide repeats are shown below the table. Click here for file

Additional file 6 ***Pfmsp1 *block2 Mad 20-types deposited in the Genbank database**. This file lists the Genbank accession number of the deposited Mad20-type alleles, along with the repeat motifs coded as indicated. 52 alleles were identified, numbered 1-52. Note that several alleles have been observed in multiple settings and/or on multiple occasions. The geographic origin is shown, when indicated in the deposited sequence or in the corresponding publication. Click here for file

Additional file 7 ***Pfmsp1 *block2 MR-type alleles deposited in the Genbank database**. This file lists the Genbank accession number of the deposited Mad20/RO33-hybrid alleles, along with the repeat motifs coded as indicated. The geographic origin is shown, when indicated in the deposited sequence or in the corresponding publication. Click here for file

Additional file 8 **Published *Pfmsp1 *block2 alleles observed in Dielmo, Senegal**. This file lists the previously described alleles that have been detected in Dielmo in this study. The name, Genbank accession number and geographic origin of the alleles deposited are indicated alongside the Dielmo alleles. Click here for file

Additional file 9 **Tripeptide combinations (tri- and di-motif combinations) displayed by the synthetic 15-mer peptide set used to monitor the anti-MSP1 block2 antibody response in Dielmo villagers**. This file shows the non overlapping tri- and di-motifs combinations observed in the deduced protein sequence of the K1- and Mad20 tripeptide repeats. Arbitrary colour codes were used to highlight the various tri- and di-motifs. Motifs are coded as indicated in Table 2. Click here for file

Additional file 10 **IgG subclass distribution for a representative set of samples from Dielmo, Senegal**. This file describes the IgG subclass distribution of 16 sera from Dielmo reacting with one or more specific *Pfmsp1 *block2-derived peptide. The ELISA plates included a positive control for each of the four sub-classes, to ascertain that absence of reactivity was not due to failure of detection of the subclass. Click here for file

Additional file 11 **Distribution of allelic families in samples collected in Dielmo during the years 1992 and 1994 from clinical malaria episodes (this work) and samples collected from asymptomatic parasites carriers (Konate L et al, *Trans R Soc Trop Med Hyg *1999, 93 Suppl 1:21-28)**. This file shows a comparison of the frequency of K1, Mad20 and RO33 families of *Pfmsp1 *block2 estimated by nested PCR genotyping in parasites collected in Dielmo from clinical malaria cases and from asymptomatic carriers in the same years. Number of samples studied: 30 and 35 samples from clinical malaria episodes (29 and 34 *Pfmsp1 *block2 PCR-positive samples) in 1992 and 1994, respectively; 77 and 144 samples from asymptomatic parasites carriers (67 and 136 *Pfmsp1 *block2 PCR-positive individuals) in 1992 and 1994, respectively. Size polymorphism was estimated by agarose gel electrophoresis. Alleles were classified in 10 bp bins. Click here for file

Additional file 12 **Number of distinct *Pfmsp1 *block2 nucleotide sequences of K1- and Mad20-types displaying identical size in the set of alleles sequenced from Dielmo, Senegal**. This file shows the number of alleles displaying distinct nucleotide sequence but classified by size polymorphism (migration in agarose gel) as having the same size (in the same 10 bp bin). Click here for file
