# Supplementary figures and images for: Population diversity and antibody selective pressure to Plasmodium falciparum MSP1 block2 locus in an African malaria-endemic setting
Source: BMC Microbiol. 2009 Oct 15;9:219. doi: 10.1186/1471-2180-9-219 (PMC2770483; doi:10.1186/1471-2180-9-219)

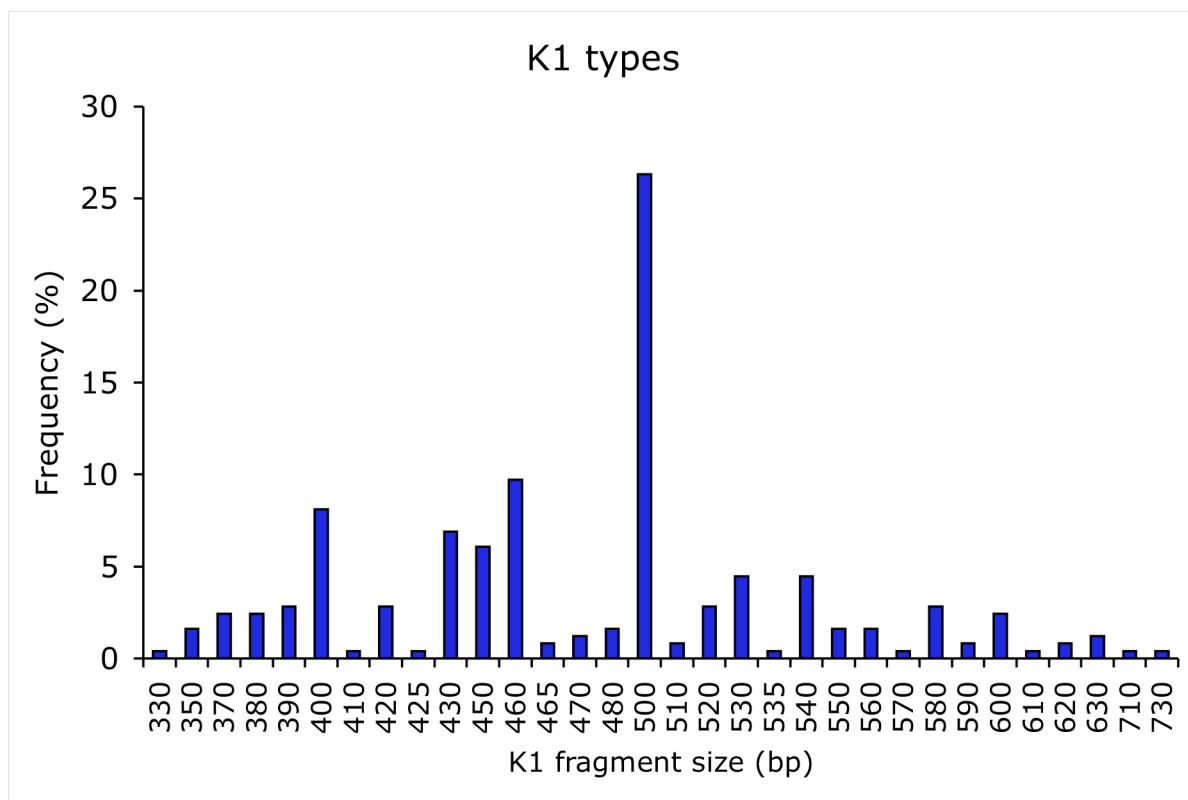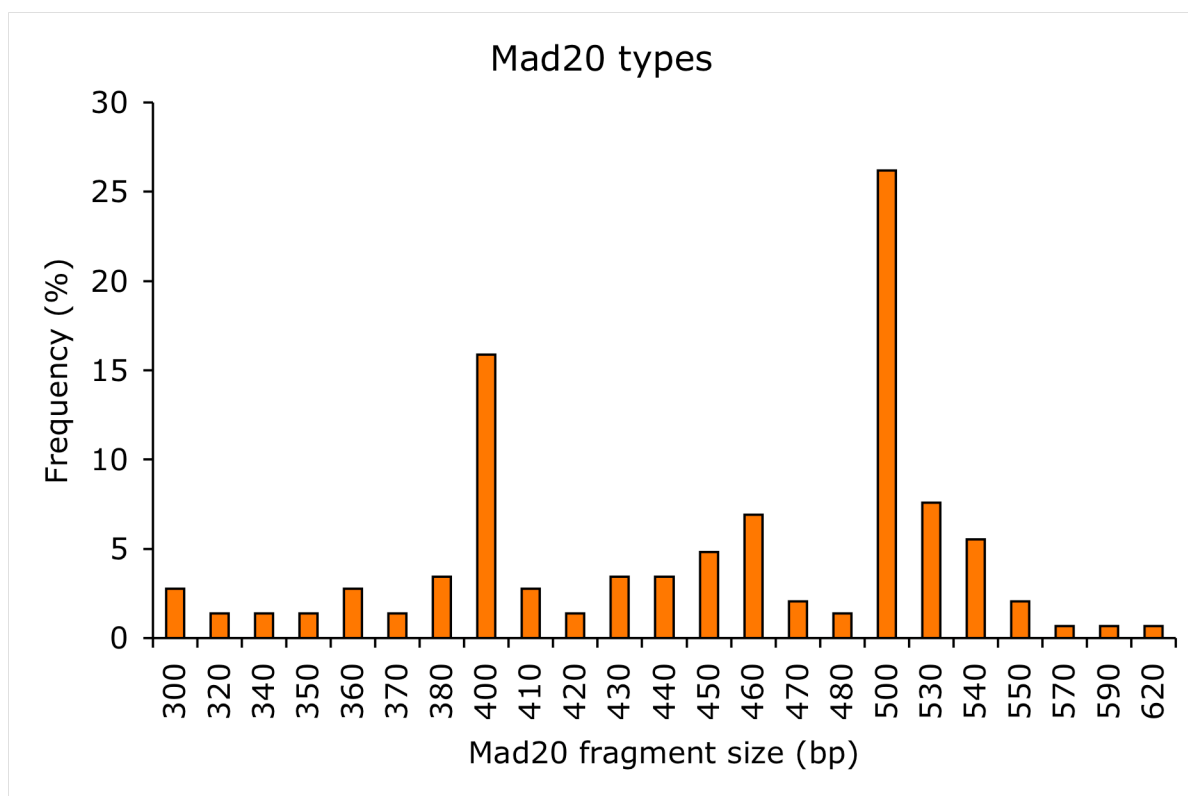

Supplement: Additional file 1 — Distribution frequency of Pfmsp1 block2 fragment size in Dielmo, Senegal. This file shows the frequency of alleles by fragment size for the K1 and MAD20 types (the RO33 alleles did not display fragment size polymorphism). The locus was amplified by semi-nested PCR and PCR products were analysed on 1.5% Nusieve:agarose gels (1:3) and visualised by ethidium bromide staining. The size of the bands was evaluated using a 100 bp DNA ladder (BioRad) as size markers. Alleles were classified in 10 bp bins. [file 1471-2180-9-219-S1.PDF]

## PCR genotyping

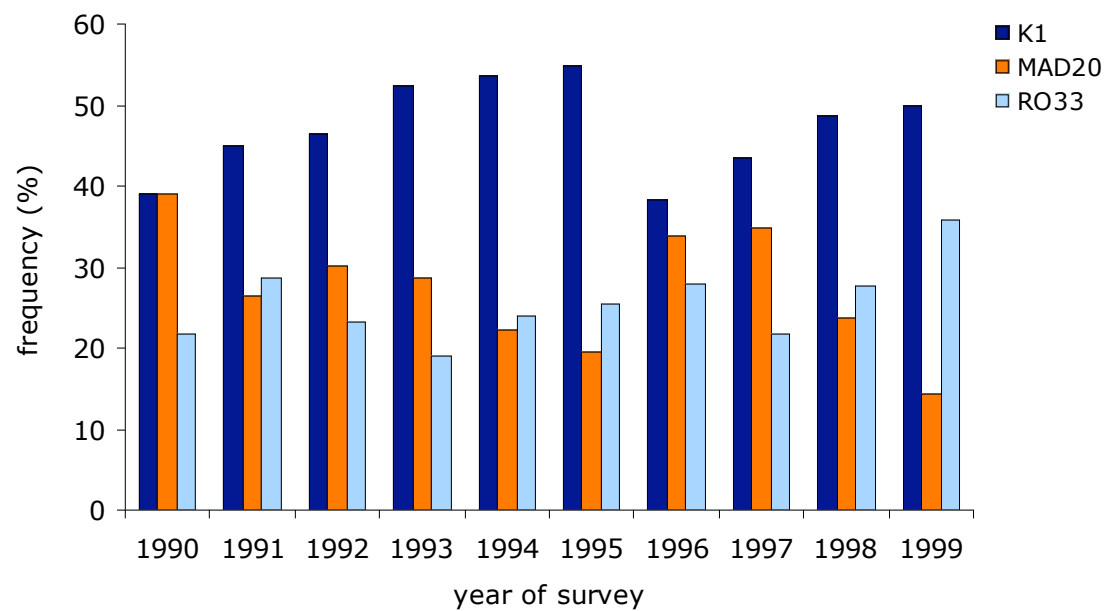

## Sequencing

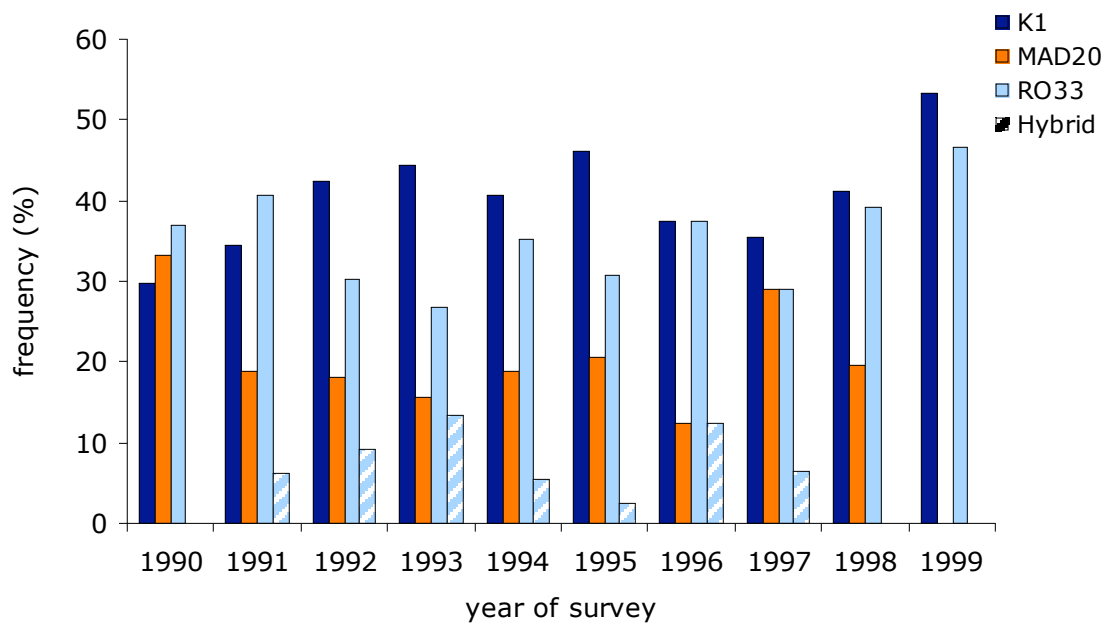

Supplement: Additional file 2 — Temporal distribution of Pfmsp1 block2 allelic families as assessed by nested PCR and sequencing. This file shows the relative distribution of the various allelic families by year as assessed either by PCR genotyping or gene sequencing. The number of samples genotyped and the number of sequences generated for each calendar year are indicated in Table 1. Sequences were determined from single PCR bands generated by family-specific nested PCR. Each sample was tested in three parallel PCR reactions triggered by one forward family specific primer and a reverse universal primer. Only the reactions generating a single band (estimated by size on agarose gels) were processed for sequencing. [file 1471-2180-9-219-S2.PDF]

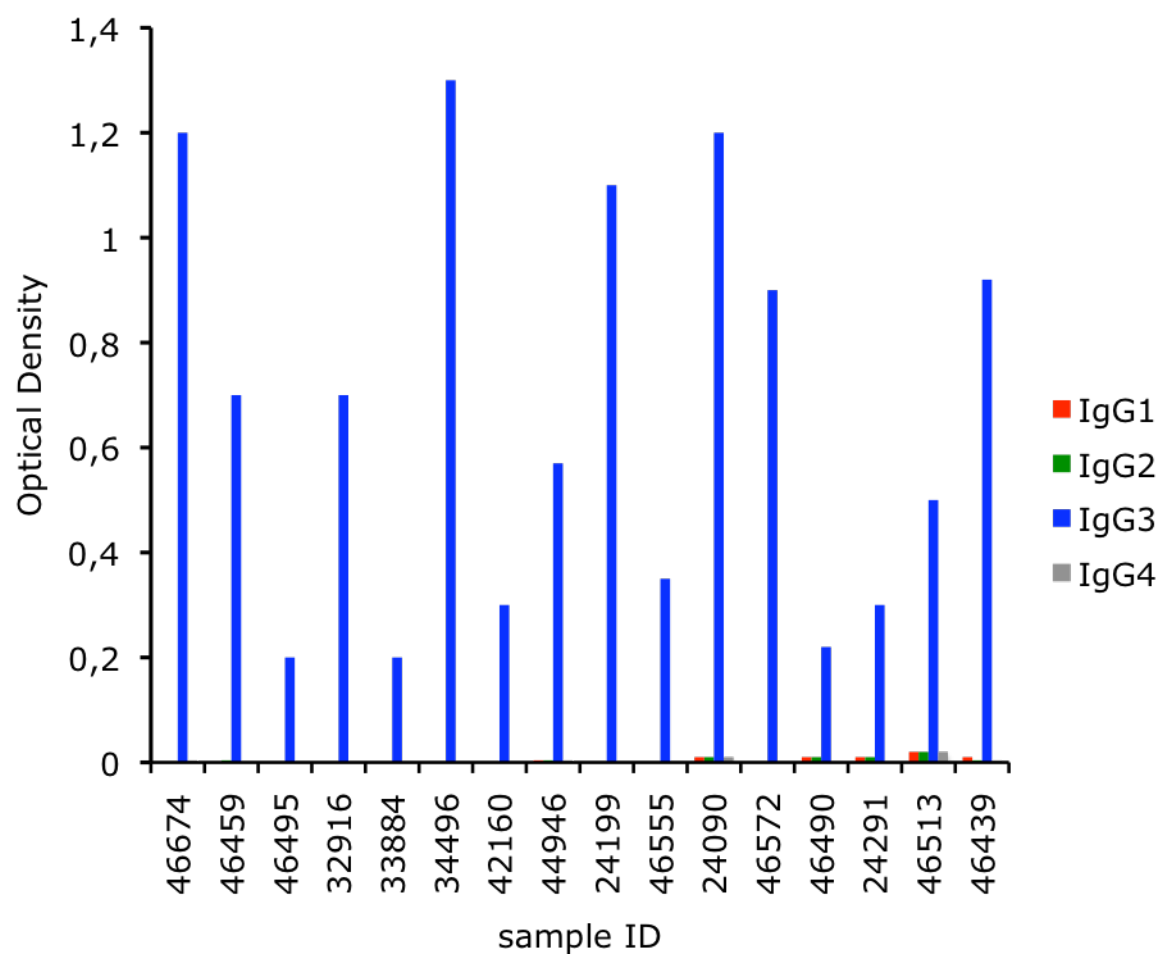

Supplement: Additional file 10 — IgG subclass distribution for a representative set of samples from Dielmo, Senegal. This file describes the IgG subclass distribution of 16 sera from Dielmo reacting with one or more specific Pfmsp1 block2-derived peptide. The ELISA plates included a positive control for each of the four sub-classes, to ascertain that absence of reactivity was not due to failure of detection of the subclass. [file 1471-2180-9-219-S10.PDF]

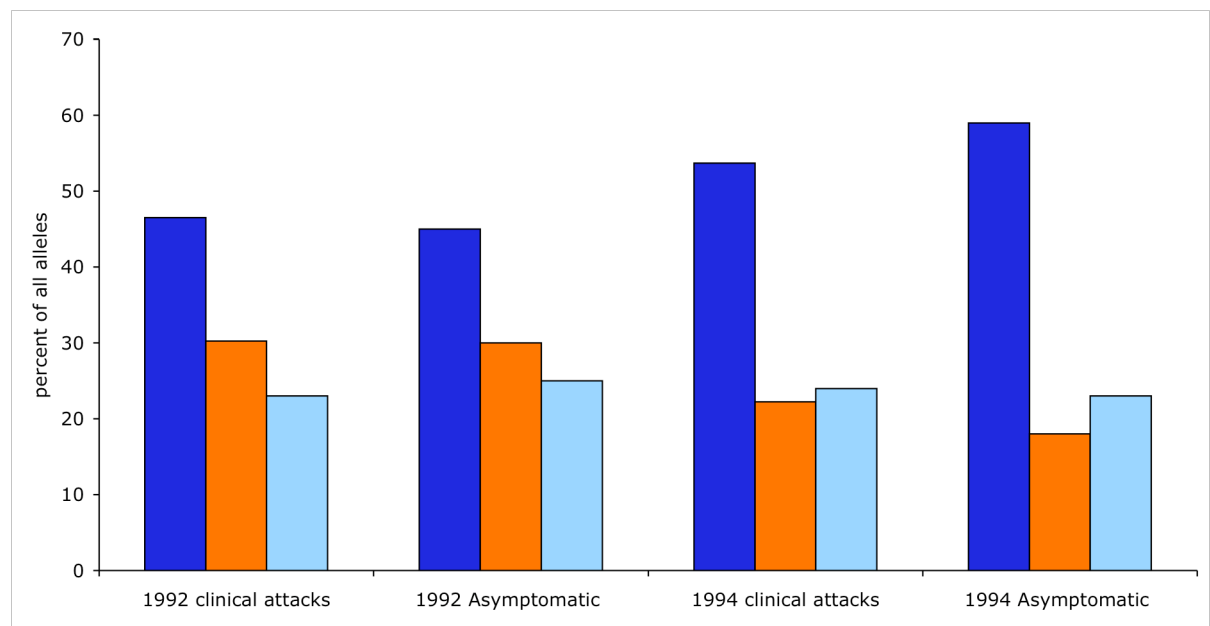

Supplement: Additional file 11 — Distribution of allelic families in samples collected in Dielmo during the years 1992 and 1994 from clinical malaria episodes (this work) and samples collected from asymptomatic parasites carriers (Konate L et al, Trans R Soc Trop Med Hyg 1999, 93 Suppl 1:21-28). This file shows a comparison of the frequency of K1, Mad20 and RO33 families of Pfmsp1 block2 estimated by nested PCR genotyping in parasites collected in Dielmo from clinical malaria cases and from asymptomatic carriers in the same years. Number of samples studied: 30 and 35 samples from clinical malaria episodes (29 and 34 Pfmsp1 block2 PCR-positive samples) in 1992 and 1994, respectively; 77 and 144 samples from asymptomatic parasites carriers (67 and 136 Pfmsp1 block2 PCR-positive individuals) in 1992 and 1994, respectively. Size polymorphism was estimated by agarose gel electrophoresis. Alleles were classified in 10 bp bins. [file 1471-2180-9-219-S11.PDF]

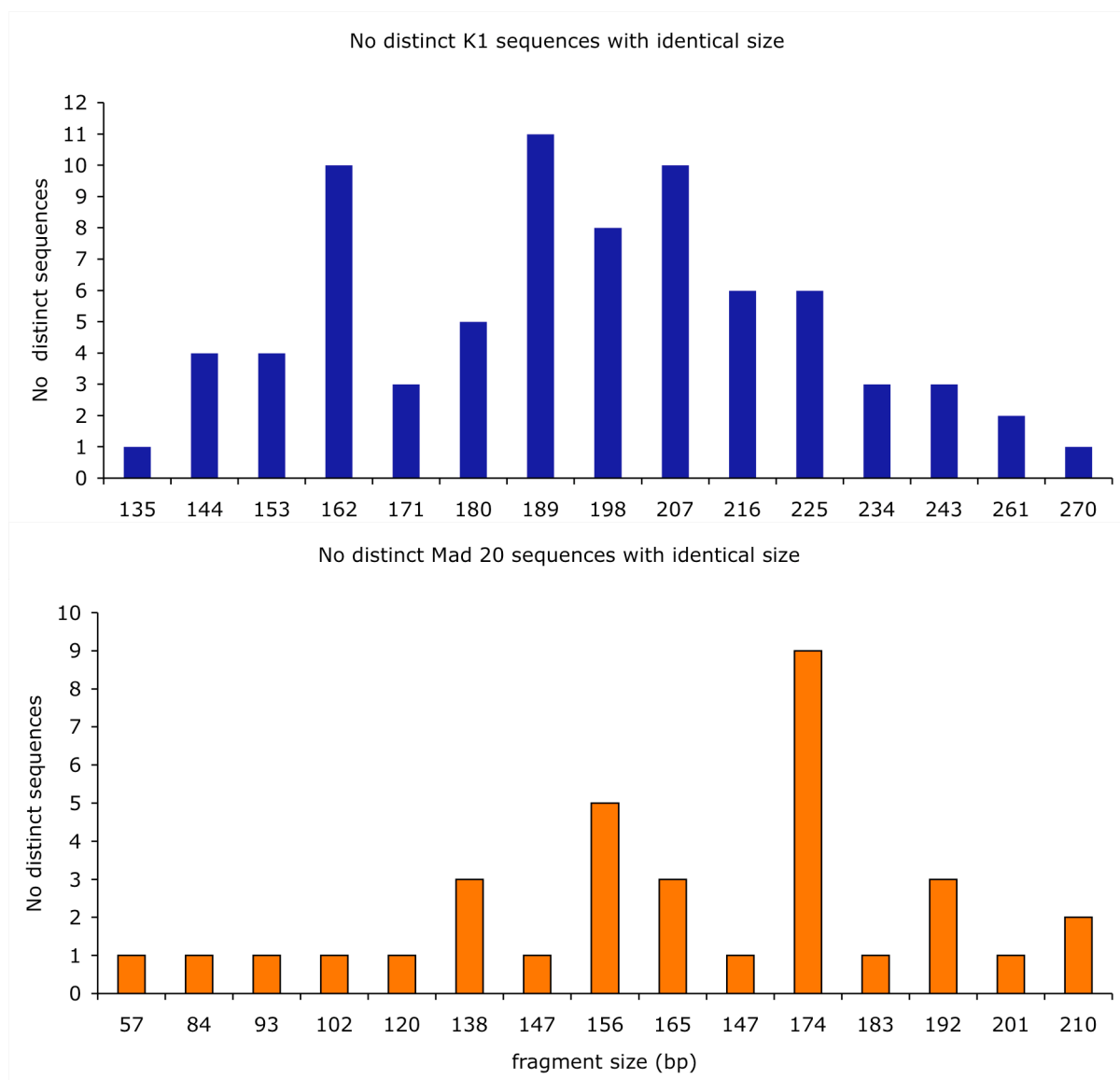

Supplement: Additional file 12 — Number of distinct Pfmsp1 block2 nucleotide sequences of K1- and Mad20-types displaying identical size in the set of alleles sequenced from Dielmo, Senegal. This file shows the number of alleles displaying distinct nucleotide sequence but classified by size polymorphism (migration in agarose gel) as having the same size (in the same 10 bp bin). [file 1471-2180-9-219-S12.PDF]
