# Supplementary material for: Population diversity and antibody selective pressure to Plasmodium falciparum MSP1 block2 locus in an African malaria-endemic setting
Source: BMC Microbiol. 2009 Oct 15;9:219. doi: 10.1186/1471-2180-9-219 (PMC2770483; doi:10.1186/1471-2180-9-219)
Supplement: Additional file 3 — Pfmsp1 block2 RO33-types deposited in the Genbank database. This file lists the Genbank accession number of the deposited RO33-type alleles, along with the country of origin of the samples, and the sequence in single amino acid code. For references see the main text. [file 1471-2180-9-219-S3.PDF]

allele	Isolate	Accession number	Origin	Author	Peptide sequence	
1	RO-33	Y00087	Ghana	Certa (1987)	MVLKDGANTQVVAKPADAVSTQSAKNPPGATVPSGTASTKGAIRSPGAANPSDDS	SDSDAKSYADLKHRVQNYLFTIKELKYPELFDLTNHMLTLCDNIHGFKYLIDG
	RO-33 Ghana	M35727	Ghana	Certa (1987)	MVLKDGANTQVVAKPADAVSTQSAKNPPGATVPSGTASTKGAIRSPGAANPSDDS	SDSDAKSYADLKHRVQNYLFTIKELKYPELFDLTNHMLTLCDNIHGFKYLIDG
	clone 841D	L10380	Thailand	Jongwutiwes (1992)	MVLKDGANTQVVAKPADAVSTQSAKNPPGATVPSGTASTKGAIRSPGAANPSDDS	SDSDAKSYADLNIEFKITCSLLKNSNIPNSLI
	isolate IFA18	AF061151	Tanzania	Jiang (2000)	MVLKDGANTQVVAKPADAVSTQSAKNPPGATVPSGTASTKGAIRSPGAANPSDDS	SDSDAKSYADLKHRVQNYLFTIKELKYPELFDLTNHMLTLCDNIHGFKYLID
	isolate IFA5.13	AF061148	Tanzania	Jiang (2000)	MVLKDGANTQVVAKPADAVSTQSAKNPPGATVPSGTASTKGAIRSPGAANPSDDS	SDSDAKSYADLKHRVQNYLFTIKELKYPELFDLTNHMLTLCDNIHGFKY
	Senegal S-10	M55001	Senegal	Scherf (1991)	MVLKDGANTQVVAKPADAVSTQSAKNPPGATVPSGTASTKGAIRSPGAANPSDDS	SDSDAKSYADLKHRVQNYLFTIKELKYPELFDLTNHMLTLCDNIHGFKYLIDGS
	isolate IFA17.29	AF061150	Tanzania	Jiang (2000)	MVLKDGANTQVVAKPADAVSTQSAKNPPGATVPSGTASTKGAIRSPGAANPSDDS	SDSDAKSYADLKHRVQNYLFTIKELKYPELFDLTNHMLTLCDNIHGFKYLIDGY
	clone 814C	M77737	Thailand	Jongwutiwes (1992)	MVLKDGANTQVVAKPADAVSTQSAKNPPGATVPSGTASTKGAIRSPGAANPSDDS	SDSDAKSYADLKHRVQNYLFTIKELKYPELFDLTNHMLTLCDNIHGFKYLIDGY
	clone 814B	M77735	Thailand	Jongwutiwes (1992)	MVLKDGANTQVVAKPADAVSTQSAKNPPGATVPSGTASTKGAIRSPGAANPSDDS	SDSDAKSYADLKHRVQNYLFTIKELKYPELFDLTNHMLTLCDNIHGFKYLIDGY
	isolate CH09	AY138507	Iran	Zakeri (2002)	MVLKDGANTQVVAKPADAVSTQSAKNPPGATVPSGTASTKGAIRSPGAANPSDDS	SDSDAKSYADLKHRVQNYLFTIKELKYPELFDLTNHMLTLCDNIHGFKYLIDGY
2	isolate IFA7	AF061149	Tanzania	Jiang (2000)	MVLKDGANTQVVAKPADAVSTQSAKNPPGATVPSGTASTKGAIRSPDAANPSDDS	SDSDAKSYADLKHRVQNYLFTIKELKYPELFDLTNHMLTLCDNIHGFKYLIDGY
3	strain B411	M32115	Brazil	Kimura (1990)	MVLKDGANTQVVAKPAGAVSTQSAKNPPGATVPSGTASTKGAIRSPGAANPSDDS	SDSDAKSYADLKHRVQNYLFTIKELKYPELFDLTNHMLTLSKNVDGFKYLIDG
	isolate:97B224-63	AB116599	Vanuatu	Tanabe (2004)	MVLKDGANTQVVAKPAGAVSTQSAKNPPGATVPSGTASTKGAIRSPGAANPSDDS	SDSDAKSYADLKHRVQNYLFTIKELKYPELFDLTNHMLTLSKNVDGFKYLIDGY
	isolate:96M320-50	AB116600	Vanuatu	Tanabe (2004)	MVLKDGANTQVVAKPAGAVSTQSAKNPPGATVPSGTASTKGAIRSPGAANPSDDS	SDSDAKSYADLKHRVQNYLFTIKELKYPELFDLTNHMLTLSKNVDGFKYLIDGY
	isolate:97B317-40	AB116601	Vanuatu	Tanabe (2004)	MVLKDGANTQVVAKPAGAVSTQSAKNPPGATVPSGTASTKGAIRSPGAANPSDDS	SDSDAKSYADLKHRVQNYLFTIKELKYPELFDLTNHMLTLSKNVDGFKYLIDGY
	clone 838	M77736	Thailand	Jongwutiwes (1992)	MVLKDGANTQVVAKPAGAVSTQSAKNPPGATVPSGTASTKGAIRSPGAANPSDDS	SDSDAKSYADLKHRVQNYLFTIKELKYPELFDLTNHMLTLCDNIHGFKYLIDGY
	isolate CH10	AY138508	Iran	Zakeri (2002)	MVLKDGANTQVVAKPAGAVSTQSAKNPPGATVPSGTASTKGAIRSPGAANPSDDS	SDSDAKSYADLKHRVQNYLFTIKELKYPELFDLTNHMLTLSKNVDGFKYLIDGY
	strain B342	M32112		Kimura (1990)	MVLKDGANTQVVAKPAGAVSTQSAKNPPGATVPSGTASTKGAIRSPGAANPSDDS	SDSDAKSYADLKHRAQNYLFTIKELKYPELFDLTNHMLTLPKNVDGFKYLIDG
	isolate:97S304-57	AB116598		Tanabe (2004)	MVLKDGANTQVVAKPAGAVSTQSAKNPPGATVPSGTASTKGAIRSPGAANPSDDS	SDSNTKTYADLKHRVQNYLFTIKELKYPELFDLTNHMLTLSKNVDGFKYLIDGY
	isolate 4/M1	AF191064		Eisen (1999)	MVLKDGANTQVVAKPAGAVSTQSAKNPPGATVPSGTASTKGAIRSPGAANPSDDS	SDSNTKTYADLKHRVQNYLFTIKEL
4	strain B440	M32114		Kimura (1990)	MVLKDGANTQVVAKPVPAVSTQSAKNPPGATVPSGTASTKGAIRSPGAANPSDDS	SDSDAKSYADLKHRVQNYLFAIKELKYPELFDLTNHMLTLSKNVDGFKYLIDG
					block 2	block3
