# Supplementary material for: Population diversity and antibody selective pressure to Plasmodium falciparum MSP1 block2 locus in an African malaria-endemic setting
Source: BMC Microbiol. 2009 Oct 15;9:219. doi: 10.1186/1471-2180-9-219 (PMC2770483; doi:10.1186/1471-2180-9-219)
Supplement: Additional file 4 — Sequence analysis of the Dielmo alleles and comparison with the alleles reported in the literature and in the databases. This file provides a detailed analysis of the molecular variation of the repeat motifs (number, sequence and arrangement) and of the point mutations observed in the various alleles from Dielmo and a comparative analysis with the alleles deposited in Genbank. [file 1471-2180-9-219-S4.RTF]

Pfmsp1 block2 sequence analysis and comparison with published sequences
K1-types
There were 77 distinct K1 alleles, named DK1-77 (Table 2), mainly differing in tripeptide copy number and sequence arrangement together with limited sequence polymorphism in the 3' family-specific unrepeated sequence. A non-synonymous S to L (tca>tta) mutation in codon 17 of the 24-residue family specific region located at the end of block2 was observed in three alleles. Allele DK67 contained moreover a six amino acid insertion, SPPADA, in the same family specific domain. A novel repeat motif encoding the SVT tripeptide reported so far in the Mad20-types (motif 7) was observed. This motif was assigned to K1 because of the typical AGT codon for Ser as opposed to a TCA/G codon in the Mad20 types and a third codon (ACA) differing from the ACT codon in Mad20 motif 7.
All K1-type alleles contained ≥ 1 copy of motifs 1 and 3 (see panel B below), while motifs 2 and 4 were observed in 83% and 64% of the alleles, respectively, and motif 7 in two alleles only. The mean number of tripeptide motifs per allele was 13.56. Motif 1 had the highest copy number, with a mean of 6.36 per allele (see panel C below). Motifs 2, 3 and 4 were present with a mean of 2.53, 3.09 and 1.48 copies per allele, respectively. Eleven di-motif combinations (hexapeptides) were observed (panel B), the most abundant being 1 1, detected with a mean of 3.44 copies/allele (panel C). The di-motifs 3 1, 1 2, 2 1 and 2 2 were present with a mean of more than one copy per allele. Importantly, motif arrangement was not random, with some quite frequent motif combinations.
A clear dichotomy could be delineated based on presence/absence of motifs 4 and 7 and on the first 5' di-motif being either 3 1 (group 1, 28 alleles) or 3 4 (group 2, 49 alleles). In both groups, there was evidence for an ordered arrangement at the 5' end. The repeat region of group 2 started with 3 4 [(3 4)n (3 1)1-3], with n = 0-7 depending on the allele, followed by a tripeptide combination of variable length and type. The repeat region of group 1 was more flexible, with the 5' 3 1 di-motif followed by (1 1)1–6 (17 alleles), (1 2)3–5 (3 alleles), (2 1)1–3 (6 alleles) and (3 1)2 (1 allele).
Mad20 types
Overall there were 34 distinct Mad20-type alleles, named DM1-34 (Table 2). The non-repeated region upstream from the tripeptide motifs was identical in all alleles. A 9 amino acid deletion (NSRRTNPSD) was observed in three alleles, but otherwise the family-specific region downstream from the repeats was monomorphic. Polymorphism was mainly due to variations in the number and arrangement of six tripeptide motifs (coded 5-9). There were two synonymous sequences coding for SGG, shown as 5 and 5 in Table 2.
All Mad20-type alleles contained the SGG-peptide sequence motif (panel E below), 97% harboured motif 6, while motifs 7 and 8 were observed in 24% and 77% of the alleles, respectively. Motif 9 was rare. The mean number of tripeptide nucleotide sequence motifs was 13.53 per allele. At the protein level there was a mean of 7.7 SGG motifs /allele and an average of 4.8 copies of SVA (motif 6) per allele (panel F). In contrast, motifs 7, 8 and 9 were always single copy. At the nucleotide level, 100% and 94% alleles contained motif 5 and 5, respectively (panel G). Only three nucleotide sequence motifs were present in multiple copies in each allele, namely motifs 5, 5 and 6 (panel H). Fifteen and 11 di-motif combinations were observed at the nucleotide and predicted protein levels, respectively (panel G and E, respectively). Di-motifs SVA SGG and SGG SVA were the most frequent (with a mean of >4 copies per allele) (panel F).
The DM alleles also presented an ordered arrangement, with a group-specific 5' end followed by a variable copy number and arrangement of six di-motifs (5 5, 5 5, 6 5, 6 5, 5 6, and 5 6), which at the protein level translated into variable combinations of the SGG and SVA tripeptide sequences (Table 2). There was a dichotomy within the family, based on the first 5' motif, being either 5/5 (group 1, 8 alleles) or 8 (group 2, 26 alleles). There were three sub-types within group1: 1a) [5 6 5]3-6 (alleles DM1-3, which presented furthermore a nine residue deletion in the family-specific region); 1b) [(5 6)3-4 5] (alleles DM4-5); and 1c) [(5 7 5) (5)0-1 5 6 5 6 5 5 (6 5) 0-1 6 5] (alleles DM6-8). There were also three sub-types within group 2: 2a) [8 5 6], 2b) [8 6 5] or 2c) [8 7 5], followed by varying combinations of 5, 5 and 6 motifs (alleles DM10-15, DM16-28, DM30-34, respectively). All Mad20-type block2 sequences except two (DM9 and DM29) terminated with the (5 6 5) sequence.
RO33 types
Unlike the other two family types, the RO33-types displayed no length variation. Six alleles were observed that differed by point mutations (Table 2). In addition to the previously reported G97D polymorphism [9] (Supporting file S3), four novel positions were dimorphic (Q72E, K90N, G91D and D104N). Allele RD5 was a G97D D104N double mutant.
Hybrid families 
Sequencing showed that 22 fragments assigned to the Mad20 family by semi-nested PCR were indeed Mad20/RO33 (MR) hybrids. Eight MR alleles were observed, named DMR1-8. Allele DMR1 derived from sub-type 1c, while alleles DMR 2-8 derived from sub-type 2c. All DMR alleles carried the same 28-residue long, RO33-type downstream region, which interestingly was a RD5 allelic type with a G97D D104N double mutation (Table 2).
A novel hybrid, that was called DMRK, was detected. Its 5' non-repeat sequence and the repeat motifs were of sub-type 2c, but the family-specific 3' region was a RO33-K1 hybrid (the K1 sequence located in 3' is underlined in Table 2).

Comparison with published msp1 block2 sequences
Comparison of sequence variations in all four family types as well as the arrangement and copy number of tripeptide repeats suggested local structuring of the alleles in Dielmo.
When compared to the reported K1 alleles (see Supporting file S5), the K1-types from Dielmo presented several specific features: i) the presence of motif 7, not observed previously, ii) a more diverse set of building blocks (i.e. more distinct tripeptide motifs per allele) (Mann-Whitney, p<0.0001) (panel A below); iii) an approx. 5-fold increase in the frequency of motif 4 (Pearson Chi2 = 32, 1df, p<0.0001) (panel B) and consequently a more frequent occurrence of the 3 4 and 4 3 di-motifs (Pearson Chi2 = 32, 1df, p<0.0001 for each); iv) a lower occurrence of the di-motifs 1 1 and 1 2 (Pearson Chi2 = 6.3 and 4.5, 1df, p=0.012 and 0.034, respectively) (panel B); v) a higher mean copy number per allele for motifs 3 and 4 (Mann-Whitney, p = 0.002 and p<0.0001, respectively) and of the 3 4 and 4 3 di-motifs (Mann-Whitney, p<0.0001 for both) (panel C). Otherwise, the architecture of the two sub-groups identified in Dielmo and delineated above was also identified in the deposited alleles (archetypical group1 and group2 alleles from laboratory lines are K1 and 3D7, respectively). The mean number of 13.56 tripeptide motifs per K1-type allele in Dielmo was no different from the average of 12.47 tripeptide motifs/allele in the published haplotypes (2-tailed t-test, p = 0.096).
Mad20-type polymorphism in Dielmo was more restricted than in the 52 distinct haplotypes identified within the 126 Mad20 sequences retrieved from the database (see Supporting file S6). Motif 4 (present in 11.5% of the Mad20 types deposited in the database) and the rare 1, 5, 5*, 6, 6* and 10-12 were not observed, and consequently, the mean number of nucleotide sequence motifs/allele was lower (2 tailed t-test, p= 0.036) (panel D, upper figure). 
Some motifs were more frequent in Dielmo such as motifs 5 and 8 (panel G) (Pearson Chi2 = 7.69, 1 df, p= 0.006, and Chi2 = 10.8, 1 df, p= 0.001, respectively), while motif 7 was less common (Pearson Chi2 = 13.14, 1 df, p< 0.001). Interestingly, the Mad20-types from Dielmo were larger than the reported alleles (mean number of tripeptide motifs/allele 13.53 vs. 11.25; Mann-Whitney, p<0.001), with a higher copy number of SGG-, SVA- and SKG-encoding motifs per allele (Mann Whitney, p<0.001, p<0.001and p= 0.001, respectively), but a lower average copy number of SVT-encoding motifs (Mann Whitney, p<0.001) (panel F). Only 15 di-motif combinations were observed at the nucleotide level in Dielmo compared to 42 in the deposited alleles. At the predicted protein level, 11 di-motif combinations were observed in Dielmo, compared to 27 in the deposited alleles (panel E). The use of di-motifs was different, with the di-motifs SGG SGG (Mann Whitney, p=0.008), SGG SVA and SVA SGG (Mann Whitney p<0.0001 each) and SKG SVA (t-test, p=0.012) being more abundant and the di-motifs SGG SVT and SVT SGG (Mann Whitney, p<0.001 and t-test, p<0.001, respectively) and SVA SVA (Mann Whitney, p<0.001) being less frequent. Furthermore, the 5/5 vs. 8 dichotomy observed for the first tripeptide repeat in Dielmo was less pronounced in the database, with 21, 4, 21 and 6 alleles commencing with motif 5, 6, 8 and 9, respectively. No deposited allele presented the characteristics described above for sub-types 1a and 1b, but 17 had a sub-type 1c signature (e.g. the laboratory line FCC1). Three, seven and eight deposited alleles had sub-type 2a, 2b and 2c signature, respectively. In contrast, additional sub-types observed in some deposited alleles (8 4 5, 8 5 5, 9 6 5 and 9 7 5) were not observed in Dielmo. Furthermore, the C-terminal block2 sequences of Mad20-types from Dielmo were less diverse than the reported alleles. 
All DMR alleles from Dielmo differed from the MR alleles from Kenya Thailand, Venezuela and India [11, 16] that contained a wild type RO33-Ghana (called here RD0) rather than a RD5 sequence (Supporting file S7). The only reported MR allele with a RD5 sequence is accession number DQ447647, of unreported geographic origin. But, interestingly all DMR alleles and the DMRK allele presented the same Mad20/RO33 boundary as the alleles with a RO33-Ghana progenitor [11, 16].

Allele distribution and frequency
For the K1 family, only 29 alleles were observed with an intra-family frequency >1%, the highest reaching 9.7% (allele DK65). The other 48 K1-types were rare. As for the Mad20 family, 15 of the 34 alleles were observed more than once, the most frequent one (allele DM11) having a frequency of 8.9% within the family. Interestingly, six of the nine DMR alleles were observed more than once; the most frequent one being DMR4 (observed in 5 isolates). Allele distribution within the RO33 family was markedly different, with a dominant RD0 allele observed in 78% (97 of 124) sequenced RO33-types alleles. The second most frequent allele was RD5 (12% frequency within the family).
At the population level, most alleles were rare (i.e. presented an intra population frequency below 1%). There were only 19 alleles present at a >1% frequency in the population sample studied here, namely DK12, DK52, DK59, DK62, DK65, DK66, DK75, DK76, DM5, DM9, DM11, DM31, DMR3, DMR4, DMR6, RD0, RD2, RD3 and RD5. In terms of frequency, the largest contribution among these top 19 alleles comes from the RO33 family. Interestingly, only four of the top 19 alleles have been reported elsewhere in the world, (DK59, DK65, DMR4 and RD0), suggesting local diversification of the parasite population. 
Temporal clustering was observed for certain alleles. For example, DK73, DM28 and DK66 were observed only during years 1991, 1997 and 1998, respectively, DM9 only during 1990-2, DK54 and DK62 only during 1995-6.


A

B

C


D


E


F


G


H
