# Supplementary material for: Population diversity and antibody selective pressure to Plasmodium falciparum MSP1 block2 locus in an African malaria-endemic setting
Source: BMC Microbiol. 2009 Oct 15;9:219. doi: 10.1186/1471-2180-9-219 (PMC2770483; doi:10.1186/1471-2180-9-219)
Supplement: Additional file 5 — Pfmsp1 block2 K1-types deposited in the Genbank database or published in the literature. This file lists the Genbank accession number of the deposited K1-type alleles, along with the repeat motifs coded as indicated. 59 distinct alleles were identified, numbered 1-59. Several alleles have been observed in multiple settings and/or on multiple occasions. The geographic origin is shown, when indicated in the deposited sequence or in the corresponding publication. The codes used for the tripeptide repeats are shown below the table. [file 1471-2180-9-219-S5.PDF]

allele	Accession number	Isolate name	Origin	Repeat sequence
1	AF061121	IFA4	Kilombero,Tanzania	3 1 1 2 1
	AF061127	IFA5.15	Kilombero,Tanzania	3 1 1 2 1
	AF509640	MSP1Ar5	not indicated	3 1 1 2 1
	AF509666	MSP1R66	Brazil	3 1 1 2 1
	AF509707	PFMSP120	not indicated	3 1 1 2 1
	AF509717	PFMSP149b	Brazil?	3 1 1 2 1
	AF509719	PFMSP1Am08	Brazil?	3 1 1 2 1
	not available*	Z31	Zambia	3 1 1 2 1
2	M77733	844	Thailand?	3 1 1 2 2 1
3	AF061123	IFA92	Kilombero,Tanzania	3 1 1 1 1 2 1
	not available*	Z26	Zambia	3 1 1 1 1 2 1
	not available*	Z26	Zambia	3 1 1 1 1 2 1
4	AF061134	IFA5.5	Kilombero,Tanzania	3 1 1 1 2 2 1
	AF061135	IFA9.16	Kilombero,Tanzania	3 1 1 1 2 2 1
	AF509651	MSP1Bl44	Viet Nam	3 1 1 1 2 2 1
	M77730	834B	Thailand?	3 1 1 1 2 2 1
	X03371	K1	Thailand	3 1 1 1 2 2 1
	not available*	Z28	Zambia	3 1 1 1 2 2 1
5	AF034792	Sudan 41	Sudan	3 1 1 2 1 2 1
	AF061131	IFA9.26	Kilombero,Tanzania	3 1 1 2 1 2 1
	AF061132	IFA17.3	Kilombero,Tanzania	3 1 1 2 1 2 1
	not available*	Z32	Zambia	3 1 1 2 1 2 1
	not available*	Z32	Zambia	3 1 1 2 1 2 1
	not available*	Z32	Zambia	3 1 1 2 1 2 1
	not available*	Z32	Zambia	3 1 1 2 1 2 1
	not available*	Z32	Zambia	3 1 1 2 1 2 1
6	AF509672	MSP1V154	Viet Nam	3 1 1 1 1 2 2 1
	AF509701	MSP1V62	Viet Nam	3 1 1 1 1 2 2 1
7	AF191061	1/M1	Indonesia: Irian Jaya province	3 1 1 1 2 2 2 1
	not available*	Z29	Zambia	3 1 1 1 2 2 2 1
8	AF509630	MSP1209	not indicated	3 1 1 1 1 1 2 2 1
	AF509675	MSP1V20	Viet Nam	3 1 1 1 1 1 2 2 1
	AF509688	MSP1V368	Viet Nam	3 1 1 1 1 1 2 2 1
	AY943292	T9/102	Thailand	3 1 1 1 1 1 2 2 1
9	DQ485424	Ke7	India: Keonjhar, Orissa	3 1 1 1 2 1 2 2 1
10	AF061133	IFA19	Kilombero,Tanzania	3 1 1 2 1 2 1 2 1
	AY695441	HP-39	India: Himachal Pradesh	3 1 1 2 1 2 1 2 1
11	AB116596	KK isolate:97S325	not indicated	3 1 1 1 1 1 1 2 2 1
	AF509648	MSP1Bl216	Viet Nam	3 1 1 1 1 1 1 2 2 1
	AF509680	MSP1V327	Viet Nam	3 1 1 1 1 1 1 2 2 1
	DQ377137	704AB	Kenya	3 1 1 1 1 1 1 2 2 1
	DQ485421	Su26	India: Sundergarh, Orissa	3 1 1 1 1 1 1 2 2 1
	M77729	814A	Thailand?	3 1 1 1 1 1 1 2 2 1
	M77731	841A	Thailand?	3 1 1 1 1 1 1 2 2 1
	M77732	841B	Thailand?	3 1 1 1 1 1 1 2 2 1
	M77734	946	Thailand?	3 1 1 1 1 1 1 2 2 1
	not available*	Z22	Zambia	3 1 1 1 1 1 1 2 2 1
12	DQ377136	S599	Kenya	3 1 3 1 3 1 1 2 2 1
13	DQ404192	Haiti 135	Haiti	3 1 1 1 3 1 1 1 2 1
14	AF509633	MSP1170	not indicated	3 1 1 1 1 1 1 1 1 1 2 2 1
	X61930	RO-71	Ivory Coast	3 1 1 1 1 1 1 1 1 1 2 2 1
15	AF509655	MSP1C	not indicated	3 1 1 2 1 2 1 2 1 2 1 2 1
	AF509657	MSP1G	not indicated	3 1 1 2 1 2 1 2 1 2 1 2 1
	AF509658	MSP1H	not indicated	3 1 1 2 1 2 1 2 1 2 1 2 1
	AF509662	MSP1R36	not indicated	3 1 1 2 1 2 1 2 1 2 1 2 1
	AF509663	MSP1R41	not indicated	3 1 1 2 1 2 1 2 1 2 1 2 1
	AF509665	MSP1R56	not indicated	3 1 1 2 1 2 1 2 1 2 1 2 1
	AF509710	PFMSP127	not indicated	3 1 1 2 1 2 1 2 1 2 1 2 1
	AF509711	PFMSP131OC	not indicated	3 1 1 2 1 2 1 2 1 2 1 2 1
	AF509712	PFMSP132a	Brazil?	3 1 1 2 1 2 1 2 1 2 1 2 1
	AF509714	PFMSP14	Brazil?	3 1 1 2 1 2 1 2 1 2 1 2 1
	AF509718	PFMSP162	Brazil?	3 1 1 2 1 2 1 2 1 2 1 2 1
16	AF061129	IFA78	Kilombero,Tanzania	3 1 1 2 1 2 1 2 1 2 2 2 1
	AF061130	IFA93	Kilombero,Tanzania	3 1 1 2 1 2 1 2 1 2 2 2 1
17	AY714586	404	Brazil?	3 1 1 1 1 2 1 1 1 1 1 2 2 1
18	AF061126	IFA16	Kilombero,Tanzania	3 1 1 1 1 1 1 3 1 1 1 1 1 2 1
19	AF034636	Sudan 12	Sudan	3 1 1 2 1 2 1 2 1 2 1 2 1 2 1 2 1 1
20	U91651	V306	Gambia	3 1 1 1 1 1 1 1 1 1 1 1 1 3 1 3 1 2 2 1
21	AF509705	PFMSP111OC	not indicated	3 1 1 1 1 1 1 1 1 1 1 1 2 2 1
	AF509639	MSP1AM97	not indicated	3 1 1 1 1 1 1 1 1 1 1 1 2 2 1

	AF509637	MSP1AM82	not indicated	3 1 1 1 1 1 1 1 1 1 1 2 2 1
	AF509632	PFMSP1AM159	not indicated	3 1 1 1 1 1 1 1 1 1 1 2 2 1
22	AF061122	wild isolate	Kilombero,Tanzania	3 1 1 1 1 1 1 1 1 2 2 2 2 1
23	AF061124	IFA17.25	Kilombero,Tanzania	3 1 1 1 1 1 1 1 1 1 1 2 2 2 1
	not available*	Z11	Zambia	3 1 1 1 1 1 1 1 1 1 1 2 2 2 1
24	X03831	CAMP	(Malaysia)	3 1 1 1 1 1 1 1 3 1 1 3 1 1 3 1 1 1 1
	M37213	Uganda-Palo Alto	Uganda	3 1 1 1 1 1 1 1 3 1 1 3 1 1 3 1 1 1 1
25	AF061128	IFA17.26	Kilombero,Tanzania	3 1 1 1 1 1 1 1 1 1 1 1 1 1 1 3 1 1 3 1 1 1 1 1 1
26	AF061119	IFA2.12	not indicated	3 4 3 1 3 1 2 2 1
27	M19144	NF7	West Africa	3 4 3 4 3 4 3 1 2 2 1
28	Z35327	3D7A=NF54	unknown	3 4 3 4 3 4 3 4 3 4 3 1 2 2 1
	AL929358	3D7genome	unknown	3 4 3 4 3 4 3 4 3 4 3 1 2 2 1
	X52963	3D7A	unknown	3 4 3 4 3 4 3 4 3 4 3 1 2 2 1
	AF191063	3/M1	Indonesia: Irian Jaya province	3 4 3 4 3 4 3 4 3 4 3 1 2 2 1
29	not available*	Z1	Zambia	3 1 1 1 1 3 1 1 1 1 3 1 3 1 1 1 1 1 1 2 2 2 2 1
30	not available*	Z2	Zambia	3 1 1 1 1 1 1 3 1 3 1 1 1 1 1 1 1 2 1
31	not available*	Z3	Zambia	3 1 1 3 1 1 3 1 1 3 1 3 1 1 1 1 2 2 1
32	not available*	Z4	Zambia	3 1 1 1 3 1 3 1 3 1 3 1 1 1 1 1 2 1
	not available*	Z4	Zambia	3 1 1 1 3 1 3 1 3 1 3 1 1 1 1 1 2 1
	not available*	Z4	Zambia	3 1 1 1 3 1 3 1 3 1 3 1 1 1 1 1 2 1
33	not available*	Z5	Zambia	3 1 1 3 1 3 1 3 1 3 1 3 1 1 1 2 1
34	not available*	Z6	Zambia	3 1 1 1 1 1 1 1 1 1 1 1 1 1 2 2 1
35	not available*	Z7	Zambia	3 1 1 1 1 3 1 1 1 1 1 1 1 2 1 1
36	not available*	Z8	Zambia	3 1 1 1 1 1 3 1 1 1 1 1 1 2 1
37	not available*	Z9	Zambia	3 1 1 1 1 3 1 1 3 1 1 1 2 1 1
38	not available*	Z10	Zambia	3 1 1 1 1 1 3 1 3 1 3 1 2 1 2 1
39	not available*	Z12	Zambia	3 1 1 1 3 1 1 3 1 1
40	not available*	Z13	Zambia	3 1 1 1 1 1 1 1 1 1 1 2 1
	not available*	Z13	Zambia	3 1 1 1 1 1 1 1 1 1 1 2 1
41	not available*	Z14	Zambia	3 1 1 1 1 1 1 1 1 1 2 2 2 1
42	not available*	Z15	Zambia	3 1 3 1 3 1 3 1 1 2 2 2
43	not available*	Z16	Zambia	3 1 1 1 1 1 1 1 1 2 2 2
44	not available*	Z17	Zambia	3 1 1 1 3 1 1 1 2 2 2 2
45	not available*	Z18	Zambia	3 1 1 1 1 1 1 1 1 2 2
	not available*	Z18	Zambia	3 1 1 1 1 1 1 1 1 2 2
46	not available*	Z19	Zambia	3 1 1 1 1 1 1 1 1 2 2 2
47	not available*	Z20	Zambia	3 4 3 1 3 1 3 1 1 1 2 1
48	not available*	Z21	Zambia	3 1 1 1 1 1 1 1 2 1
49	not available*	Z23	Zambia	3 1 1 1 3 1 2 2 2 1
50	not available*	Z24	Zambia	3 1 1 1 1 1 2 2 2 1
51	not available*	Z25	Zambia	3 4 3 1 3 1 3 1 2 1 2 2 1
52	not available*	Z27	Zambia	3 1 1 1 2 2 2 2 2 2 2 2 1
53	not available*	Z30	Zambia	3 4 3 4 3 1 1 1
	not available*	Z30	Zambia	3 4 3 4 3 1 1 1
54	not available*	Z33	Zambia	3 1 1 2 2 2 2 2 2 1
55	not available*	Z34	Zambia	3 1 1 2 1 2 1 2 1 2 1 2 1 2 2 2 1
56	AF061125	IFA6		3 4 3 4 3 1 2 2 1
	not available*	Z35	Zambia	3 4 3 4 3 1 2 2 1
57	not available*	Z36	Zambia	3 4 3 4 3 4 3 1 2 2 1
	not available*	Z36	Zambia	3 4 3 4 3 4 3 1 2 2 1
58	not available*	Z37	Zambia	3 4 3 4 3 4 3 1 2 2 2 2 2 2 1
59	not available*	Z38	Zambia	3 4 3 4 3 4 3 4 3 1 2 2 1

* peptide sequence see Tetteh et al 2005 (Infect Immun 73, 5928-35)

code for K1 types	peptide sequence	nucleotide sequence
1	SGT	AGT GGT ACA
2	SGP	AGT GGT CCA
3	SAQ	AGT GCT CAA
4	SGA	AGT GGT GCA
7#	SVT	AGT GTT ACA

observed in Dielmo Senegal, not reported in the databases
