# Supplementary material for: Population diversity and antibody selective pressure to Plasmodium falciparum MSP1 block2 locus in an African malaria-endemic setting
Source: BMC Microbiol. 2009 Oct 15;9:219. doi: 10.1186/1471-2180-9-219 (PMC2770483; doi:10.1186/1471-2180-9-219)
Supplement: Additional file 6 — Pfmsp1 block2 Mad 20-types deposited in the Genbank database. This file lists the Genbank accession number of the deposited Mad20-type alleles, along with the repeat motifs coded as indicated. 52 alleles were identified, numbered 1-52. Note that several alleles have been observed in multiple settings and/or on multiple occasions. The geographic origin is shown, when indicated in the deposited sequence or in the corresponding publication. [file 1471-2180-9-219-S6.PDF]

allele	accession number	Isolate	Origin	Repeat sequence
1	AF509636.1	MSP1AM78	not indicated	5 1 5 5 5 5 4 5 4 5 5 4 5 6 5
2	DQ485429.1	Su15	India: Sundergarh, Orissa	5 5 6 5 6 5 5 6 6 5 6 5
3	AF509642.1	MSP1BL146	Viet Nam	5 7 5 1 5 5 6 6 6 5
	AF509645.1	MSP1Bl185	Viet Nam	5 7 5 1 5 5 6 6 6 5
	AF509646.1	MSP1193	Viet Nam	5 7 5 1 5 5 6 6 6 5
	AF509647.1	MSP1Bl199	Viet Nam	5 7 5 1 5 5 6 6 6 5
	AF509650.1	MSP1Bl37	Viet Nam	5 7 5 1 5 5 6 6 6 5
	AF509670.1	MSP1V12	Viet Nam	5 7 5 1 5 5 6 6 6 5
	AF509685.1	MSP1V354	Viet Nam	5 7 5 1 5 5 6 6 6 5
	AF509687.1	MSP1V365	Viet Nam	5 7 5 1 5 5 6 6 6 5
	AF509694.1	MSP1V43	Viet Nam	5 7 5 1 5 5 6 6 6 5
	AF509702.1	MSP1V72	Viet Nam	5 7 5 1 5 5 6 6 6 5
4	DQ485432.1	Ke1	India: Keonjhar, Orissa	5 7 5 5 12 5 6 5
5	AF509638.1	MSP1AM89	not indicated	5 7 5 5 5 5 4 5 6 5 5 6 5 6 5
	AF509667.1	MSP1R92	Brazil	5 7 5 5 5 5 4 5 6 5 5 6 5 6 5
	AF509709.1	PFMSP125	not indicated	5 7 5 5 5 5 4 5 6 5 5 6 5 6 5
6	AF176817.1	94/98	Brazil: Maracaja, Para	5 7 5 5 5 5 6 5 6 5 5 6 5 6 5
	AF509656.1	MSP1D98	not indicated	5 7 5 5 5 5 6 5 6 5 5 6 5 6 5
	AF509659.1	MSP1lm2624	not indicated	5 7 5 5 5 5 6 5 6 5 5 6 5 6 5
	AF509660.1	MSP1lm7	not indicated	5 7 5 5 5 5 6 5 6 5 5 6 5 6 5
	AF509661.1	MSP1R129	not indicated	5 7 5 5 5 5 6 5 6 5 5 6 5 6 5
	AF509664.1	MSP1R53	Brazil	5 7 5 5 5 5 6 5 6 5 5 6 5 6 5
	AF509704.1	PFMSP110OC	not indicated	5 7 5 5 5 5 6 5 6 5 5 6 5 6 5
	AF509708.1	PFMSP122OC	not indicated	5 7 5 5 5 5 6 5 6 5 5 6 5 6 5
	AF509713.1	PFMSP134	not indicated	5 7 5 5 5 5 6 5 6 5 5 6 5 6 5
	AF509715.1	PFMSP142	not indicated	5 7 5 5 5 5 6 5 6 5 5 6 5 6 5
	AF509716.1	PFMSP145	not indicated	5 7 5 5 5 5 6 5 6 5 5 6 5 6 5
7	AY714585.1	57	Brazil	5 7 5 5 5 5 6 5 6 5 5 6 5 6 5
8	AF034635	SUDAN7	Sudan	5 7 5 5 6 5 5 6 10 5 6 5
9	AF061138	IFA10	Tanzania	5 7 5 5 6 5 5 6 5 5 6 5 6 5
10	AF061137	IFA9.10	Tanzania	5 7 5 5 6 5 9 6 5 5 6 5 6 5
11	AF061142	HN1	Tanzania	5 7 5 5 6 6 5 5 6 5
	AF062348	HN1	China	5 7 5 5 6 6 5 5 6 5
	AF218248	FCC1/HN	China	5 7 5 5 6 6 5 5 6 5
	AF251345	FCC1/HN	China	5 7 5 5 6 6 5 5 6 5
	AY695438.1	TR-9	India: Tripura	5 7 5 5 6 6 5 5 6 5
	DQ377135.1	620B	Kenya	5 7 5 5 6 6 5 5 6 5
	M77722.2	clone 835A	Thailand	5 7 5 5 6 6 5 5 6 5
	X02406.1	pMC31-1	not indicated	5 7 5 5 6 6 5 5 6 5
12	M77726.2	clone 842	Thailand	5 7 5 5 6 6 5 5 6 5
13	AF061147	HN6.27	Tanzania	5 7 5 5 6 6 6 5
14	DQ485428.1	Su5	India: Sundergarh, Orissa	5 7 5 6* 11 5 6 5
15	DQ485431.1	Su18	India: Sundergarh, Orissa	5 7 5 7 5 1 6 6 6 6 5
16	AF509652.1	MSP1Bl52	Viet Nam	5 7 5 7 5 5 6 5
	AF509653.1	MSP1BI53	Viet Nam	5 7 5 7 5 5 6 5
17	DQ377133.1	687H	Kenya	5 7 5 7 5 5 6 6 6 (GCT) 5
18	AF061146	HN6.24	Tanzania	5 7 5 7 5 5 6 6 6 5
	AF509671.1	MSP1V13	Viet Nam	5 7 5 7 5 5 6 6 6 5
	AF509673.1	MSP1V16	Viet Nam	5 7 5 7 5 5 6 6 6 5
	AF509683.1	MSP1V338	Viet Nam	5 7 5 7 5 5 6 6 6 5
	AF509697.1	MSP1V50	Viet Nam	5 7 5 7 5 5 6 6 6 5
	DQ377134.1	706B	Kenya	5 7 5 7 5 5 6 6 6 5
19	AF509631.1	MSP1101	not indicated	5 7 5 7 5 5 6 6 6 6 5
	AF509635.1	MSP1176	Viet Nam	5 7 5 7 5 5 6 6 6 6 5
	AF509644.1	MSP1BL182	Viet Nam	5 7 5 7 5 5 6 6 6 6 5
	AF509674.1	MSP1V19	Viet Nam	5 7 5 7 5 5 6 6 6 6 5
	AF509678.1	MSP1V303	Viet Nam	5 7 5 7 5 5 6 6 6 6 5
	AF509689.1	MSP1V369	Viet Nam	5 7 5 7 5 5 6 6 6 6 5
	AF509690.1	MSP1V377	Viet Nam	5 7 5 7 5 5 6 6 6 6 5
	AF509698.1	MSP151 V51	Viet Nam	5 7 5 7 5 5 6 6 6 6 5
	AF509700.1	MSP161 V61	Viet Nam	5 7 5 7 5 5 6 6 6 6 5
	DQ404191.1	Indochina I/CDC	Viet Nam	5 7 5 7 5 5 6 6 6 6 5
	M77721.2	clone 834A	Thailand	5 7 5 7 5 5 6 6 6 6 5
20	AF509649.1	MSP1Bl33	Viet Nam	5 7 5 7 5 7 5 5 6 6 6 6 5
21	DQ485427.1	So3	India: Sonapur, Assam	5 7 5 7 5 7 6 6 6 6 5
	DQ485430.1	Su17	India: Sundergarh, Orissa	5 7 5 7 5 7 6 6 6 6 5
	M77728.2	clone 947	Thailand	5 7 5 7 5 7 6 6 6 6 5
22	M77714.2	clone 807	Thailand	5 7 5 7 5 7 6 6 6 6 6 5
	M77716.2	clone 815	Thailand	5 7 5 7 5 7 6 6 6 6 6 5
	M77723.2	clone 835B	Thailand	5 7 5 7 5 7 6 6 6 6 6 5
	M77724.2	clone 836	Thailand	5 7 5 7 5 7 6 6 6 6 6 5
23	D43960.1	Pf83	not indicated	6 5 5 6 5 5 6 5 6 5
24	AF061141	IFA125	Tanzania	6 5 6 5 5 6 5 5 6 5 6 5

	DQ485425.1	isolate WB5	India: Darjeeling, West Bengal	6 5 6 5 5 6 5 5 6 5 6 5
25	AF061140	IFA12	Tanzania	6 5 6 5 5 6 5 5 6 5 6 5
26	X13784.1	Palo Alto	not indicated	8 4 5 5 6 5 6 5 6 5
27	AF061144	HN3	Tanzania	8 5 5 5 6 6 5 6 6 5
	AF061145	HN5	Tanzania	8 5 5 5 6 6 5 6 6 5
28	AF061143	HN2	Tanzania	8 5 6 5 5 5 6 6 5 6 6 5
	AF062349	strain HN2	China	8 5 6 5 5 5 6 6 5 6 6 5
29	AF061139	IFA11	Tanzania	8 5 6 5 5 6 5 5 6 5 6 5 6 5
30	AF509691.1	MSP1V378	Viet Nam	8 5 6 5 5 6 6 5 6 6 5
31	AY695440.1	UP-87	India: Utter Pradesh	8 6 5
32	AF509681.1	MSP1V331	Viet Nam	8 6 5 5 6 5 5 6 5 6 4 6 5
	AF509693.1	MSP1V42	Viet Nam	8 6 5 5 6 5 5 6 5 6 4 6 5
33	AF509634.1	MSP1172 AM172	Viet Nam	8 6 5 5 6 5 6 5
	AJ635200.1	Amiri7	Honduras	8 6 5 5 6 5 6 5
	M14632.1	Honduras I/CDC	Honduras	8 6 5 5 6 5 6 5
	X52962.1	HB3A	Honduras	8 6 5 5 6 5 6 5
34	AF480451.1	FCB-1	Colombia	8 6 5 5 6 5 6 5 6 5 6 5
	M77713.2	clone 806	Thailand	8 6 5 5 6 5 6 5 6 5 6 5
	M77715.2	clone 808	Thailand	8 6 5 5 6 5 6 5 6 5 6 5
	M77718.2	clone 822B	Thailand	8 6 5 5 6 5 6 5 6 5 6 5
	M77719.2	clone 827	Thailand	8 6 5 5 6 5 6 5 6 5 6 5
	M77725.2	clone 837	Thailand	8 6 5 5 6 5 6 5 6 5 6 5
	X02919.1	P195	not indicated	8 6 5 5 6 5 6 5 6 5 6 5
	X15063.1	Palo Alto PLF-3/B11	not indicated	8 6 5 5 6 5 6 5 6 5 6 5
	X63185.1	FVO	Viet Nam	8 6 5 5 6 5 6 5 6 5 6 5
35	X13782.1	Senegalese BANDIA	Senegal	8 6 5 5 6 5* 5 6 5 6 5 6 5
36	AF509696.1	MSP1V49	Viet Nam	8 6 5 5 6 6 5 6 5
37	DQ485426.1	So11	India: Sonapur, Assam	8 6 5 6 5 6 5 6 5 5 6 6 5
38	M77727.2	clone 843	Thailand	8 6 9 6 5
39	AJ635201.1	Amiri7	Honduras	8 7 5 5 5 5 6 5
40	AF509684.1	MSP1V341	Viet Nam	8 7 5 5 6 5 4 6 5 5 6 5 6 5
41	AF509641.1	MSP1BL128	Viet Nam	8 7 5 5 6 5 5 6 5 5 6 5
	AF509643.1	MSP1BL177	Viet Nam	8 7 5 5 6 5 5 6 5 5 6 5
	AF509676.1	MSP1V207	Viet Nam	8 7 5 5 6 5 5 6 5 5 6 5
	AF509692.1	MSP1V379	Viet Nam	8 7 5 5 6 5 5 6 5 5 6 5
	AF509695.1	MSP1V46	Viet Nam	8 7 5 5 6 5 5 6 5 5 6 5
42	AF509669.1	MSp1V10	Viet Nam	8 7 5 5 6 5 5 6 5 5 6 5 6 5
	AF509677.1	MSP1V209	Viet Nam	8 7 5 5 6 5 5 6 5 5 6 5 6 5
	AF509679.1	MSP1V314	Viet Nam	8 7 5 5 6 5 5 6 5 5 6 5 6 5
	AF509686.1	MSP1V360	Viet Nam	8 7 5 5 6 5 5 6 5 5 6 5 6 5
	AF509699.1	MSP157 V60	Viet Nam	8 7 5 5 6 5 5 6 5 5 6 5 6 5
	AF509703.1	MSP1W2	Viet Nam	8 7 5 5 6 5 5 6 5 5 6 5 6 5
43	AF509682.1	MSP1V334	Viet Nam	8 7 5 5 6 5 5 6 5 5 6 5 6 5 6 5
44	AF061136	IFA9.2	Tanzania	8 7 5 5 6 5 5 6 5 6 5 6 5
45	AY947642.1	G1	not indicated	8 7 5 5 6 5 5 6 5 6 5 6 5 6 5 6 5
46	M77720.2	clone 828	Thailand	8 7 5 7 5 7 6 6 6 6 6 5
47	M32116.1	strain B439	not indicated	9 6 5 6 6 5 5 6 5 6 5
48	AB116597.1	97S304-19	not indicated	9 7 5 5 6 5 5 6 5 5 6 5
49	M77717.2	clone 822A	Thailand	9 7 5 5 6 6 4 5
50	AF509654.1	MSP1Bl61	Viet Nam	9 7 5 5 6 6 5 5
51	AF191062.1	2/M1	Indonesia: Irian Jaya province	9 7 5 5 6 6 6 5 5 6 5
52	M19143.1	FC27	Papua New Guinea	9 7 5 6 6 6 5 5 6 5
	X05624.2	MAD20	Papua New Guinea	9 7 5 6 6 6 5 5 6 5

code	peptide sequence	nucleotide sequence
5	SGG	TCA GGT GGT
5	SGG	TCA GGT GGC
5	SGG	TCA GGC GGT
5*	SGG	TCG GGT GGT
4	SGA	TCA GGT GCT
1	SGT	TCA GGT ACT
10	LGG	TTA GGT GGT
6	SVA	TCA GTT GCT
6	SVA	TCA GTG GCT
6*	SVA	TCG GTG GCT
11	SVG	TCA GTT GGT
7	SVT	TCA GTT ACT
8	SKG	TCA AAG GGT
9	SSG	TCA AGT GGT
12	FSC	TTC AGT TGT
