# Supplementary material for: Population diversity and antibody selective pressure to Plasmodium falciparum MSP1 block2 locus in an African malaria-endemic setting
Source: BMC Microbiol. 2009 Oct 15;9:219. doi: 10.1186/1471-2180-9-219 (PMC2770483; doi:10.1186/1471-2180-9-219)
Supplement: Additional file 7 — Pfmsp1 block2 MR-type alleles deposited in the Genbank database. This file lists the Genbank accession number of the deposited Mad20/RO33-hybrid alleles, along with the repeat motifs coded as indicated. The geographic origin is shown, when indicated in the deposited sequence or in the corresponding publication. [file 1471-2180-9-219-S7.PDF]

allele	Accession no.	Isolate	Origin	repeat sequence
1	AF462449	S10-9	Kenya	8 7 5 5 **4**
2	AF462450	S4-5	Kenya	8 7 5 5 5 **4**
3	AY826429	S12-17	Thailand	8 7 5 5 5 5 **4**
4	AY826430	S12-19	Thailand	8 7 5 5 5 5 **4**
5	AF462452	S8-7	Kenya	8 7 5 5 5 5 **4**
6	AF462453	S10-7	Kenya	8 7 5 5 5 5 5 **4**
7	AF462454	S10-1	Kenya	8 7 5 5 5 5 5 5 **4**
8	AF462455	S5-5	Kenya	8 7 5 5 5 5 5 5 5 5 **4**
9	AY826431	S11-3	Venezuela	8 7 5 5 5 5 5 5 5 5 5 5 5 **4**
10	DQ447647		unreported	8 7 5 5 5 **4**

code	peptide sequence	nucleotide sequence
5	SGG	TCA GGT GGT
5	SGG	TCA GGT GGC
**4**	SGA	TCA GGT GCT
7	SVT	TCA GTT ACT
8	SKG	TCA AAG GGT
8	SKG	TCG AAG GGT
