# Supplementary material for: Population diversity and antibody selective pressure to Plasmodium falciparum MSP1 block2 locus in an African malaria-endemic setting
Source: BMC Microbiol. 2009 Oct 15;9:219. doi: 10.1186/1471-2180-9-219 (PMC2770483; doi:10.1186/1471-2180-9-219)
Supplement: Additional file 8 — Published Pfmsp1 block2 alleles observed in Dielmo, Senegal. This file lists the previously described alleles that have been detected in Dielmo in this study. The name, Genbank accession number and geographic origin of the alleles deposited are indicated alongside the Dielmo alleles. [file 1471-2180-9-219-S8.PDF]

	Dielmo	database		
family	code	name	Acc No	Geogr. Origin
K1	DK5	PFMSP1110c	AF509705	Brazil
		MSP1AM97	AF509639.1	
		MSP1AM82	AF509637.1	
		PFMSP1AM15[illegible]	AF509632.1	
	DK7	RO-71	X61930	Ivory Coast
		MSP1170	AF509633.1	
	DK9	Su26	DQ485421.1	India
		704AB	DQ377137.1	Kenya
		814A	M77729.2	Thailand
		946	M77734.2	Thailand
		841B	M77732.2	Thailand
		841A	M77731.2	Thailand
		MSP1V327	AF5096801.	Viet Nam
		MSP1BI216	AF509648.1	Viet Nam
		97S325-14	AB116596.1	?
		Z22	-	Zambia
	DK18	PFMSP162	AF509718.1	Brazil
		PFMSP14	AF509714.1	
		PFMSP132a	AF509712.1	
		PFMSP1310C	AF509711.1	
		PFMSP127	AF509710.1	
		MSP1R56	AF509665.1	
		MSP1R41	AF509663.1	
		MSP1R36	AF509662.1	
		MSP1H	AF509658.1	Brazil
		MSP1G	AF509657.1	
		MSP1C	AF509655.1	
	DK59	IFA6	AF061125.1	Tanzania
		Z35	-	Zambia
	DK65	NF7	M19144	Ghana
		Z36	-	Zambia
	DK70	Z38	-	Zambia
	DK74	NF54	Z35327	unknown
		3D7 genome	AL929358.1	
		3/M1	AF191063.1	Irian Jaya
MAD20	DM 29	843	M77727	Thailand
	DM 34	MSp1V10	AF509669.1	VietNam
		MSP1V209	AF509677.1	VietNam
		MSP1V314	AF509679.1	VietNam
		MSP157 V60	AF509699.1	VietNam
		MSP1W2	AF509703.1	VietNam
RO33	RD0	RO33	Y00087	Ghana
		RO33 cDNA	M35727	Ghana
	RD4	IFA7	AF061149	Tanzania
MR	DMR4		DQ447647	unreported
