# Supplementary material for: Population diversity and antibody selective pressure to Plasmodium falciparum MSP1 block2 locus in an African malaria-endemic setting
Source: BMC Microbiol. 2009 Oct 15;9:219. doi: 10.1186/1471-2180-9-219 (PMC2770483; doi:10.1186/1471-2180-9-219)
Supplement: Additional file 9 — Tripeptide combinations (tri- and di-motif combinations) displayed by the synthetic 15-mer peptide set used to monitor the anti-MSP1 block2 antibody response in Dielmo villagers. This file shows the non overlapping tri- and di-motifs combinations observed in the deduced protein sequence of the K1- and Mad20 tripeptide repeats. Arbitrary colour codes were used to highlight the various tri- and di-motifs. Motifs are coded as indicated in Table 2. [file 1471-2180-9-219-S9.PDF]

K1 types triad peptides

DK1	3	1	1	1	1	1	1	1	1	1	1	1	1	1	1	2	1	SPS	SRS						
DK2	3	1	1	1	1	1	1	1	1	1	1	1	1	1	2	2	1	SPS	SRS						
DK3	3	1	1	1	1	1	1	1	1	1	1	1	2	2	1	SPS	SRS								
DK4	3	1	1	1	1	1	1	1	1	1	1	1	3	1	3	1	2	2	1	SPS	SRS				
DK5	3	1	1	1	1	1	1	1	1	1	1	2	2	1	SPS	SRS									
DK6	3	1	1	1	1	1	1	1	1	1	1	3	1	3	1	3	1	1	1	1	2	1	SPS	SPS	SRS
DK7	3	1	1	1	1	1	1	1	1	1	2	2	1	SPS	SRS										
DK8	3	1	1	1	1	1	1	1	2	2	2	2	1	SPS	SRS										
DK9	3	1	1	1	1	1	1	2	2	1	SPS	SRS													
DK10	3	1	1	1	1	1	1	2	2	2	2	2	2	2	2	2	1	SPS	SRS						
DK11	3	1	1	1	1	3	1	3	1	3	1	1	1	2	1	SPS	SRS								
DK12	3	1	1	1	1	3	1	3	1	3	1	1	2	2	1	SPS	SRS								
DK13	3	1	1	1	1	3	1	3	1	3	1	2	1	SPS	SRS										
DK14	3	1	1	1	1	2	1	2	1	2	1	1	2	2	1	SPS	SRS								
DK15	3	1	1	1	2	1	2	1	2	1	2	2	2	1	SPS	SRS									
DK16	3	1	1	1	3	1	1	1	3	1	1	1	3	1	2	2	1	SPS	SRS						
DK17	3	1	1	1	3	1	1	3	1	1	3	1	1	2	2	1	SPS	SRS							
DK18	3	1	1	2	1	2	1	2	1	2	1	2	1	SPS	SRS										
DK19	3	1	1	2	1	2	1	2	1	2	2	1	SPS	SRS											
DK20	3	1	1	2	1	2	1	2	2	1	SPS	SRS													
DK21	3	1	2	1	2	1	2	1	1	1	1	2	2	2	2	1	SPS	SRS							
DK22	3	1	2	1	2	1	2	1	1	1	2	2	1	SPS	SRS										
DK23	3	1	2	1	2	1	2	1	2	2	1	2	2	1	SPS	SRS									
DK24	3	1	2	1	2	1	2	1	2	2	2	2	1	SPS	SRS										
DK25	3	1	2	1	2	1	2	2	1	SPS	SRS														
DK26	3	1	2	1	2	2	2	2	2	2	2	2	2	2	2	2	2	2	1	SPS	SRS				
DK27	3	1	3	1	3	1	1	1	1	1	1	1	1	1	1	1	1	1	1	SPS	SRS				
DK28	3	3	1	1	1	1	1	1	1	1	1	1	1	2	2	1	SPS	SRS							
DK29	3	4	3	1	1	1	1	1	1	SPS	SRS														
DK30	3	4	3	1	1	1	1	1	1	1	SPS	SRS													
DK31	3	4	3	1	1	1	1	1	1	1	1	1	2	2	1	SPS	SRS								
DK32	3	4	3	1	1	1	1	1	1	1	1	2	2	1	SPS	SRS									
DK33	3	4	3	1	1	1	1	1	1	2	2	1	SPS	SRS											
DK34	3	4	3	1	1	1	1	1	2	2	1	SPS	SRS												
DK35	3	4	3	1	1	1	1	2	2	1	SPS	SRS													
DK36	3	4	3	1	1	1	2	1	SPS	SRS															
DK37	3	4	3	1	1	1	2	2	2	1	SPS	SRS													
DK38	3	4	3	1	2	1	2	1	2	1	2	2	2	1	SPS	SRS									
DK39	3	4	3	2	2	1	SPS	SRS																	
DK40	3	4	3	1	2	2	2	1	SPS	SRS															
DK41	3	4	3	1	3	1	2	2	1	2	2	1	SPS	SRS											
DK42	3	4	3	1	3	1	3	1	1	1	SPS	SRS													
DK43	3	4	3	1	3	1	3	1	1	1	1	1	1	1	1	1	2		1	SPS	SRS				
DK44	3	4	3	1	3	1	3	1	1	1	1	1	1	2	1	1	2		1	SPS	SRS				
DK45	3	4	3	1	3	1	3	1	1	1	1	1	2	2	1	SPS	SRS								
DK46	3	4	3	1	3	1	3	1	1	1	1	2	2	1		SPS	SRS								
DK47	3	4	3	1	3	1	3	1	1	1	2	2	1	SPS	SRS										
DK48	3	4	3	1	3	1	3	1	1	2	1	2	1	SPS	SRS										
DK49	3	4	3	1	3	4	3	4	3	4	3	2	2	1	SPS	SRS									
DK50	3	4	3	1	7	2	2	1	SPS	SRS															
DK51	3	4	3	4	3	1	1	1	1	SPS	SRS														
DK52	3	4	3	4	3	1	1	1	1	1	SPS	SRS													
DK53	3	4	3	4	3	1	1	1	1	1	1	1	1	1	1	1	SPS	SRS							
DK54	3	4	3	4	3	1	1	1	2	2	2	1	SPS	SRS											
DK55	3	4	3	4	3	1	1	2	2	1	SPS	SRS													
DK56	3	4	3	4	3	1	1	3	4	3	2	2	1	SPS	SRS										
DK57	3	4	3	4	3	1	2	1	SPS	SRS															
DK58	3	4	3	4	3	1	2	1	2	1	2	2	2	1	2	2	2	1	2	2	1	SPS	SRS		
DK59	3	4	3	4	3	1	2	2	1	SPS	SRS														
DK60	3	4	3	4	3	1	2	2	2	1	SPS	SRS													
DK61	3	4	3	4	3	1	3	1	3	1	3	1	3	1	SPS	SRS									
DK62	3	4	3	4	3	4	3	1	1	1	1	1	1	SPS	SRS										
DK63	3	4	3	4	3	4	3	1	1	1	1	1	1	1	1	SPS	SRS								
DK64	3	4	3	4	3	4	3	1	2	1	SPS	SRS													
DK65	3	4	3	4	3	4	3	1	2	2	1	SPS	SRS												
DK66	3	4	3	4	3	4	3	1	2	2	1	1	SPS	SRS											
DK67	3	4	3	4	3	4	3	1	3	1	1	1	1	1	2	1	SPS	SRS							
DK68	3	4	3	4	3	4	3	4	3	1	1	SPS	SRS												
DK69	3	4	3	4	3	4	3	4	3	1	1	1	1	SPS	SRS										
DK70	3	4	3	4	3	4	3	4	3	1	2	2	1	SPS	SRS										
DK71	3	4	3	4	3	4	3	4	3	1	2	2	2	1	SPS	SRS									
DK72	3	4	3	4	3	4	3	4	3	1	3	1	1	1	1	1	SPS	SRS							
DK73	3	4	3	4	3	4	3	4	3	1	3	1	1	1	2	2	1	SPS	SRS						
DK74	3	4	3	4	3	4	3	4	3	4	3	1	2	2	1	SPS	SRS								
DK75	3	4	3	4	3	4	3	4	3	4	3	1	7	2	2	1	SPS	SRS							
DK76	3	4	3	4	3	4	3	4	3	4	3	4	3	1	2	2	1	SPS	SRS						
DK77	3	4	3	4	3	4	3	4	3	4	3	4	3	4	3	4	3	1	2	2	1	SPS	SRS		

K1 di-motif peptides

DK1	3	1	1	1	1	1	1	1	1	1	1	1	1	1	1	2	1	SPS	SRS					
DK2	3	1	1	1	1	1	1	1	1	1	1	1	1	1	2	2	1	SPS	SRS					
DK3	3	1	1	1	1	1	1	1	1	1	1	1	2	2	1	SPS	SRS							
DK4	3	1	1	1	1	1	1	1	1	1	1	1	3	1	3	1	2	2	1	SPS	SRS			
DK5	3	1	1	1	1	1	1	1	1	1	1	2	2	1	SPS	SRS								
DK6	3	1	1	1	1	1	1	1	1	1	1	3	1	3	1	3	1	1	1	1	2	1	SPS	SRS
DK7	3	1	1	1	1	1	1	1	1	1	2	2	1	SPS	SRS									
DK8	3	1	1	1	1	1	1	1	2	2	2	2	1	SPS	SRS									
DK9	3	1	1	1	1	1	1	2	2	1	SPS	SRS												
DK10	3	1	1	1	1	1	1	2	2	2	2	2	2	2	2	2	1	SPS	SRS					
DK11	3	1	1	1	1	3	1	3	1	3	1	1	1	2	1	SPS	SRS							
DK12	3	1	1	1	1	3	1	3	1	3	1	1	2	2	1	SPS	SRS							
DK13	3	1	1	1	1	3	1	3	1	3	1	2	1	SPS	SRS									
DK14	3	1	1	1	1	2	1	2	1	2	1	1	2	2	1	SPS	SRS							
DK15	3	1	1	1	2	1	2	1	2	1	2	2	2	1	SPS	SRS								
DK16	3	1	1	1	3	1	1	1	3	1	1	1	3	1	2	2	1	SPS	SRS					
DK17	3	1	1	1	3	1	1	3	1	1	3	1	1	2	2	1	SPS	SRS						
DK18	3	1	1	2	1	2	1	2	1	2	1	2	1	SPS	SRS									
DK19	3	1	1	2	1	2	1	2	1	2	2	1	SPS	SRS										
DK20	3	1	1	2	1	2	1	2	2	1	SPS	SRS												
DK21	3	1	2	1	2	1	2	1	1	1	1	2	2	2	2	1	SPS	SRS						
DK22	3	1	2	1	2	1	2	1	1	1	2	2	1	SPS	SRS									
DK23	3	1	2	1	2	1	2	1	2	2	1	2	2	1	SPS	SRS								
DK24	3	1	2	1	2	1	2	1	2	2	2	2	1	SPS	SRS									
DK25	3	1	2	1	2	1	2	2	1	SPS	SRS													
DK26	3	1	2	1	2	2	2	2	2	2	2	2	2	2	2	2	2	2	1	SPS	SRS			
DK27	3	1	3	1	3	1	1	1	1	1	1	1	1	1	1	1	1	1	1	SPS	SRS			
DK28	3	3	1	1	1	1	1	1	1	1	1	1	1	2	2	1	SPS	SRS						
DK29	3	4	3	1	1	1	1	1	1	SPS	SRS													
DK30	3	4	3	1	1	1	1	1	1	1	SPS	SRS												
DK31	3	4	3	1	1	1	1	1	1	1	1	1	2	2	1	SPS	SRS							
DK32	3	4	3	1	1	1	1	1	1	1	1	2	2	1	SPS	SRS								
DK33	3	4	3	1	1	1	1	1	1	2	2	1	SPS	SRS										
DK34	3	4	3	1	1	1	1	1	2	2	1	SPS	SRS											
DK35	3	4	3	1	1	1	1	2	2	1	SPS	SRS												
DK36	3	4	3	1	1	1	2	1	SPS	SRS														
DK37	3	4	3	1	1	1	2	2	2	1	SPS	SRS												
DK38	3	4	3	1	2	1	2	1	2	1	2	2	2	1	SPS	SRS								
DK39	3	4	3	2	2	1	SPS	SRS																
DK40	3	4	3	1	2	2	2	1	SPS	SRS														
DK41	3	4	3	1	3	1	2	2	1	2	2	1	SPS	SRS										
DK42	3	4	3	1	3	1	3	1	1	1	SPS	SRS												
DK43	3	4	3	1	3	1	3	1	1	1	1	1	1	1	1	1	2	1	SPS	SRS				
DK44	3	4	3	1	3	1	3	1	1	1	1	1	1	2	1	1	2	1	SPS	SRS				
DK45	3	4	3	1	3	1	3	1	1	1	1	1	2	2	1	SPS	SRS							
DK46	3	4	3	1	3	1	3	1	1	1	1	2	2	1	SPS	SRS								
DK47	3	4	3	1	3	1	3	1	1	1	2	2	1	SPS	SRS									
DK48	3	4	3	1	3	1	3	1	1	2	1	2	1	SPS	SRS									
DK49	3	4	3	1	3	4	3	4	3	4	3	2	2	1	SPS	SRS								
DK50	3	4	3	1	7	2	2	1	SPS	SRS														
DK51	3	4	3	4	3	1	1	1	1	SPS	SRS													
DK52	3	4	3	4	3	1	1	1	1	1	SPS	SRS												
DK53	3	4	3	4	3	1	1	1	1	1	1	1	1	1	1	1	SPS	SRS						
DK54	3	4	3	4	3	1	1	1	2	2	2	1	SPS	SRS										
DK55	3	4	3	4	3	1	1	2	2	1	SPS	SRS												
DK56	3	4	3	4	3	1	1	3	4	3	2	2	1	SPS	SRS									
DK57	3	4	3	4	3	1	2	1	SPS	SRS														
DK58	3	4	3	4	3	1	2	1	2	1	2	2	2	1	2	2	2	1	2	2	1	SPS	SRS	
DK59	3	4	3	4	3	1	2	2	1	SPS	SRS													
DK60	3	4	3	4	3	1	2	2	2	1	SPS	SRS												
DK61	3	4	3	4	3	1	3	1	3	1	3	1	3	1	SPS	SRS								
DK62	3	4	3	4	3	4	3	1	1	1	1	1	1	SPS	SRS									
DK63	3	4	3	4	3	4	3	1	1	1	1	1	1	1	1	SPS	SRS							
DK64	3	4	3	4	3	4	3	1	2	1	SPS	SRS												
DK65	3	4	3	4	3	4	3	1	2	2	1	SPS	SRS											
DK66	3	4	3	4	3	4	3	1	2	2	1	1	SPS	SRS										
DK67	3	4	3	4	3	4	3	1	3	1	1	1	1	1	2	1	SPS	SRS						
DK68	3	4	3	4	3	4	3	4	3	1	1	SPS	SRS											
DK69	3	4	3	4	3	4	3	4	3	1	1	1	1	SPS	SRS									
DK70	3	4	3	4	3	4	3	4	3	1	2	2	1	SPS	SRS									
DK71	3	4	3	4	3	4	3	4	3	1	2	2	2	1	SPS	SRS								
DK72	3	4	3	4	3	4	3	4	3	1	3	1	1	1	1	1	SPS	SRS						
DK73	3	4	3	4	3	4	3	4	3	1	3	1	1	1	2	2	1	SPS	SRS					
DK74	3	4	3	4	3	4	3	4	3	4	3	1	2	2	1	SPS	SRS							
DK75	3	4	3	4	3	4	3	4	3	4	3	1	7	2	2	1	SPS	SRS						
DK76	3	4	3	4	3	4	3	4	3	4	3	4	3	1	2	2	1	SPS	SRS					
DK77	3	4	3	4	3	4	3	4	3	4	3	4	3	4	3	4	3	1	2	2	1	SPS	SRS	

Mad20 types triad peptides

DM1	5	6	5	5	6	5	5	6	5	5	6	5	5	6	5	5	6	5	
DM2	5	6	5	5	6	5	5	6	5	5	6	5	5	6	5				
DM3	5	6	5	5	6	5	5	6	5										
DM4	5	6	5	6	5	6	5	6	5										
DM5	5	6	5	6	5	6	5												
DM6	5	7	5	5	5	6	5	6	5	5	6	5	6	5					
DM7	5	7	5	5	6	5	6	5	5	6	5	6	5						
DM8	5	7	5	5	5	6	5	6	5	5	6	5							
DM9	8	5																	
DM10	8	5	6	5	6	5	6	5	6	5	6	5	6	5	6	5	5	6	5
DM11	8	5	6	5	5	6	5	5	6	5	5	6	5	6	5	6	5		
DM12	8	5	6	5	5	5	6	5	6	5	5	6	5	6	5				
DM13	8	5	6	5	5	6	5	5	6	5	5	6	5	6	5				
DM14	8	5	6	6	5	6	5	5	6	5	5	6	5	6	5				
DM15	8	5	6	5	5	6	6	5	6	6	5	6	5						
DM16	8	6	5	5	6	5	6	5	5	6	5	5	6	5	5	6	5	6	5
DM17	8	6	5	6	5	6	5	5	5	6	5	6	5	6	5	6	5		
DM18	8	6	5	6	5	6	5	6	5	5	6	5	5	6	5	6	5		
DM19	8	6	5	6	5	5	5	6	5	6	5	6	5	6	5				
DM20	8	6	5	6	5	6	5	5	5	6	5	6	5	6	5				
DM21	8	6	5	6	5	6	5	5	6	5	5	6	5	6	5				
DM22	8	6	5	6	5	5	5	6	5	6	5	6	5						
DM23	8	6	5	6	5	5	6	5	5	6	5	5	6	5					
DM24	8	6	5	5	6	5	5	6	5	6	5	6	5						
DM25	8	6	5	6	5	6	5	5	5	6	5	6	5						
DM26	8	6	5	5	5	6	5	6	5	6	5								
DM27	8	6	5	5	6	5	5	6	5	6	5								
DM28	8	6	5	6	5	5	5	6	5	6	5								
DM29	8	6	9	6	5														
DM30	8	7	5	5	5	6	5	5	6	5	5	5	6	5	6	5	6	5	
DM31	8	7	5	5	5	5	6	5	5	6	5	5	6	5	6	5			
DM32	8	7	5	5	5	6	5	5	6	5	5	6	5	6	5				
DM33	8	7	5	5	5	6	5	6	5	6	5	6	5	6	5				
DM34	8	7	5	5	6	5	5	6	5	5	6	5	6	5					

Mad20 types di-motif peptides

**DM1**	5	6	5	5	6	5	5	6	5	5	6	5	5	6	5	5	6	5	
**DM2**	5	6	5	5	6	5	5	6	5	5	6	5	5	6	5				
**DM3**	5	6	5	5	6	5	5	6	5										
**DM4**	5	6	5	6	5	6	5	6	5										
**DM5**	5	6	5	6	5	6	5												
**DM6**	5	7	5	5	5	6	5	6	5	5	6	5	6	5					
**DM7**	5	7	5	5	6	5	6	5	5	6	5	6	5						
**DM8**	5	7	5	5	5	6	5	6	5	5	6	5							
**DM9**	8	5																	
**DM10**	8	5	6	5	6	5	6	5	6	5	6	5	6	5	6	5	5	6	5
**DM11**	8	5	6	5	5	6	5	5	6	5	5	6	5	6	5	6	5		
**DM12**	8	5	6	5	5	5	6	5	6	5	5	6	5	6	5				
**DM13**	8	5	6	5	5	6	5	5	6	5	5	6	5	6	5				
**DM14**	8	5	6	6	5	6	5	5	6	5	5	6	5	6	5				
**DM15**	8	5	6	5	5	6	6	5	6	6	5	6	5						
**DM16**	8	6	5	5	6	5	6	5	5	6	5	5	6	5	5	6	5	6	5
**DM17**	8	6	5	6	5	6	5	5	5	6	5	6	5	6	5	6	5		
**DM18**	8	6	5	6	5	6	5	6	5	5	6	5	5	6	5	6	5		
**DM19**	8	6	5	6	5	5	5	6	5	6	5	6	5	6	5				
**DM20**	8	6	5	6	5	6	5	5	5	6	5	6	5	6	5				
**DM21**	8	6	5	6	5	6	5	5	6	5	5	6	5	6	5				
**DM22**	8	6	5	6	5	5	5	6	5	6	5	6	5						
**DM23**	8	6	5	6	5	5	6	5	5	6	5	5	6	5					
**DM24**	8	6	5	5	6	5	5	6	5	6	5	6	5						
**DM25**	8	6	5	6	5	6	5	5	5	6	5	6	5						
**DM26**	8	6	5	5	5	6	5	6	5	6	5								
**DM27**	8	6	5	5	6	5	5	6	5	6	5								
**DM28**	8	6	5	6	5	5	5	6	5	6	5								
**DM29**	8	6	9	6	5														
**DM30**	8	7	5	5	5	6	5	5	6	5	5	5	6	5	6	5	6	5	
**DM31**	8	7	5	5	5	5	6	5	5	6	5	5	6	5	6	5			
**DM32**	8	7	5	5	5	6	5	5	6	5	5	6	5	6	5				
**DM33**	8	7	5	5	5	6	5	6	5	6	5	6	5	6	5				
**DM34**	8	7	5	5	6	5	5	6	5	5	6	5	6	5					
